# Gigaxonin Suppresses Epithelial-to-Mesenchymal Transition of Human Cancer Through Downregulation of Snail

**DOI:** 10.1158/2767-9764.CRC-23-0331

**Published:** 2024-03-08

**Authors:** Mysore S. Veena, Jungmo J. Gahng, Mustafa Alani, Albert Y. Ko, Saroj K. Basak, Isabelle Y. Liu, Kimberly J. Hwang, Jenna R. Chatoff, Natarajan Venkatesan, Marco Morselli, Weihong Yan, Ibraheem Ali, Karolina Elżbieta Kaczor-Urbanowicz, Bhavani Shankara Gowda, Patrick Frost, Matteo Pellegrini, Neda A. Moatamed, Sharon P. Wilczynski, Pascale Bomont, Marilene B. Wang, Daniel Sanghoon Shin, Eri S. Srivatsan

**Affiliations:** 1 Department of Surgery, VAGLAHS/David Geffen School of Medicine at UCLA, Los Angeles, California.; 2 Department of Molecular, Cellular and Developmental Biology, UCLA, Los Angeles, California.; 3 Department of Chemistry and Biochemistry and the Institute for Quantitative and Computational Biology, UCLA, Los Angeles, California.; 4 Department of Louise M. Darling Biomedical Library and The Institute for Quantitative and Computational Biology, UCLA, Los Angeles, California.; 5 Department of Oral Biology and Medicine, Center for Oral and Head/Neck Oncology Research, School of Dentistry, UCLA, Los Angeles, California.; 6 The Institute for Quantitative and Computational Biosciences, UCLA, Los Angeles, California.; 7 Department of Medicine, VAGLAHS/David Geffen School of Medicine at UCLA, Los Angeles, California.; 8 Department of Pathology and Laboratory Medicine, David Geffen School of Medicine at UCLA, Los Angeles, California.; 9 Department of Pathology, City of Hope National Medical Center, Duarte, California.; 10 ERC team, INMG, UCBL Lyon1 – CNRS UMR5261 – INSERM U1315, Université Lyon 1, Université de Lyon, Lyon, France.; 11 Department of Surgery, VAGLAHS and Department of Head and Neck Surgery, David Geffen School of Medicine at UCLA, Los Angeles, California.

## Abstract

Gigaxonin is an E3 ubiquitin ligase that plays a role in cytoskeletal stability. Its role in cancer is not yet clearly understood. Our previous studies of head and neck cancer had identified gigaxonin interacting with p16 for NFκB ubiquitination. To explore its role in cancer cell growth suppression, we analyzed normal and tumor DNA from cervical and head and neck cancers. There was a higher frequency of exon 8 SNP (c.1293 C>T, rs2608555) in the tumor (46% vs. 25% normal, *P* = 0.011) pointing to a relationship to cancer. Comparison of primary tumor with recurrence and metastasis did not reveal a statistical significance. Two cervical cancer cell lines, ME180 and HT3 harboring exon 8 SNP and showing T allele expression correlated with higher gigaxonin expression, reduced *in vitro* cell growth and enhanced cisplatin sensitivity in comparison with C allele expressing cancer cell lines. Loss of gigaxonin expression in ME180 cells through CRISPR-Cas9 or siRNA led to aggressive cancer cell growth including increased migration and Matrigel invasion. The *in vitro* cell growth phenotypes were reversed with re-expression of gigaxonin. Suppression of cell growth correlated with reduced Snail and increased e-cadherin expression. Mouse tail vein injection studies showed increased lung metastasis of cells with low gigaxonin expression and reduced metastasis with reexpression of gigaxonin. We have found an association between C allele expression and RNA instability and absence of multimeric protein formation. From our results, we conclude that gigaxonin expression is associated with suppression of epithelial–mesenchymal transition through inhibition of Snail.

**Significance:**

Our results suggest that *GAN* gene exon 8 SNP T allele expression correlates with higher gigaxonin expression and suppression of aggressive cancer cell growth. There is downregulation of Snail and upregulation of e-cadherin through NFκB ubiquitination. We hypothesize that exon 8 T allele and gigaxonin expression could serve as diagnostic markers of suppression of aggressive growth of head and neck cancer.

## Introduction

Giant axonal neuropathy (*GAN*) is a rare but severe neurodegenerative disorder caused by mutations of the *GAN* gene (1). This gene encodes the protein gigaxonin, which possesses E3 ubiquitin ligase properties and is involved in intermediate filament (IF) processing and degradation (1–3). Gigaxonin mutations cause accumulation of unprocessed IF which in neurons are visualized in larger (giant) appearing neurons on cytopathology (4). Among the identified partners of gigaxonin, class III and IV IFs such as peripherin, vimentin, and neurofilament proteins, as well as the autophagy protein ATG16L1 and Ptch receptor of Sonic hedgehock signaling molecule (Shh) signaling have been shown to be regulated by gigaxonin in *GAN* models (5–8). Degradation of IF is regulated through glycosylation of gigaxonin (9). Patients with *GAN* initially display peripheral motor and sensory disease symptoms at infancy, which progressively affects the central nervous system causing loss of mental function and seizures. Prognosis is poor as life expectancy is 30 years (10). Currently GAN gene therapy clinical trial is being conducted for the treatment of GAN in collaboration between National Institute of Neurological Disorders and Stroke (NINDS) and Taysha Gene Therapies, Inc. (Intrathecal Administration of scAAV9/JeT-GAN for the Treatment of Giant Axonal Neuropathy, ClinicalTrials.gov Identifier: NCT02362438).

As a distant relative of the BTB/kelch superfamily, gigaxonin is composed of a single BTB domain for interacting with Cul3 ubiquitin ligases and six kelch domains for interacting with proteins targeted for degradation (1). In patients with GAN, genomic mutations have no regional preference (6). Although GAN gene mutations are generally known for their manifestation in neural cells, they are found in primary human cancers at a low frequency. In the Catalogue Of Somatic Mutations in Cancer (COSMIC) database, there are 522 (1.4%) recorded cases of GAN gene mutations in different cancer types out of 38,170 cancer samples studied (11). Analysis of two other databases, Driver-DB and International Cancer Genome Consortium (ICGC) Data Portal, has shown cancer specific GAN gene mutations mostly in liver cancer (12–14).

The 1000 Genome Project has recorded exon 8 SNP, rs2608555 (c.1293C>T, p.Y431Y) in 27.4% of the world population (8.1%–45.6%) with the highest frequency (45.6%) in the European population (14–16). SNPs are often assumed to be non-functional, but there are examples of functional SNPs in the literature. Cystic fibrosis patients with the rs397508419 (c.2679G>T, p.G893G) polymorphism in exon 15 of the *CFTR* gene exhibit aberrant mRNA splicing, and SNPs in the *MDR1* gene affect mRNA folding that prevents cytoplasmic translocation and translation (17, 18). In patients with Duchenne muscular dystrophy, a combination of SNPs in the *SPP1* and *LTBP4* genes are associated with age at loss of ambulation (19, 20). In patients with Alzheimer's disease, SNPs are associated with variations in amyloid *β* deposition (21). Although the clinical phenotype of GAN gene exon 8 SNP is not known, it is likely that a heterozygous SNP (T and C alleles) impacts gigaxonin expression as the gene seems to be inactivated by heterozygous allelic mutations (possibly loss of T allele expression) in patients with GAN (22).

Human papillomavirus (HPV) sequences detected in >90% of cervical cancer and 15%–20% of head and neck cancer show high p16 expression (23–27). While the p16 and p53 genes are not mutated in HPV-positive tumors, p53 and Rb proteins are inactivated through ubiquitination by E6 and E7 proteins of the HPV, respectively (26, 27). In HPV-negative tumors, p16 is inactivated through deletions and promotor methylation, and p53 is inactivated through point mutations (28, 29). Inactivation of the *RB* gene by the E7 protein results in the activation of the E2F1 transcription factor which in turn activates cell cycle proteins *CDK4*, *CDK6*, and cyclin D1. Thus, in the absence of functional p53 and Rb proteins, cancer cell regulation is disrupted in human cancer. We have previously shown that p16 in association with gigaxonin ubiquitinates *NFκB*, a major transcription factor involved in the development of head and neck cancer (30). It is not known whether other transcription factors are targeted directly or indirectly through *NFκB* by gigaxonin. To understand the role of gigaxonin in tumor development, we determined the prevalence *GAN* exon 8 SNP (rs2608555) in cervical and head and neck cancers. We then investigated cancer cell lines containing homozygous exon 8 SNP (T/T) or heterozygous alleles (C/T) in their genome, relationship of T versus C allele to gigaxonin expression and its effect on cancer cell growth including cisplatin sensitivity.

## Materials and Methods

### Primary Tumors and Cancer Cell Lines

Tumor samples were obtained with Institutional Review Board approval from VAGLAHS, UCLA, and City of Hope Medical Center. Cervical cancer cell lines were obtained from ATCC at different times and UM-SCC cell lines were obtained from The University of Michigan in 2000. Cell lines were used within 5 passages and were tested before their initial use for *Mycoplasma*. Latest testing on ME180 cells was done in 2021. Cell lines were grown as described earlier in RPMI medium containing PSF and supplemented with 10% FBS and ampules were stored in liquid nitrogen tank. Cell lines were tested for *Mycoplasma* contamination using the approved kit. Cell authentication was done using mitochondrial repeat PCR primers (refs. 31, 32; Supplementary Fig. S1). Cell morphology formed second method of cell authenticity (Supplementary Fig. S2). ATCC indicates HT3 cell line as HPV negative as well as containing HPV 30 sequences. These cells have homozygous mutation of TP53 gene. Because HPV positivity is related to wild-type p53, mutated p53 could be interpreted as HPV-negative cells.

### Incucyte (Real-time Cell Imager) Cell Proliferation Assay

A total of 4,000 or 8,000 cells (ME180, GAN 15.1, Control LV 50, GAN LV 50) were seeded in quadruplicates into 96-well plates on day 0 and media was changed next day with respective treatment concentrations (cisplatin) After the media was changed, the plate was put into Incucyte imager (located at core facility of Jonsson Comprehensive Cancer Center) and the image was automatically captured every 2 hours as per machine routine setting. After the 4–5 days, the data were analyzed by the built-in software, replot and statistical analysis was performed by GraphPad Software (Prism, version 6). The experiments were performed thrice for each experiment.

### Soft Agar Colony Assay

Cells were trypsinized, and suspended in minimum essential medium containing 0.1% lukewarm agar at a cell concentration of 5 × 10^3^ cells/mL. The suspension was spread on top of 0.5% solidified agar plates. The agar plates were incubated for 12 to 30 days at 37°C. Colonies were stained with 0.001% crystal violet blue, counted, and photographed using a Zeiss microscope (30, 33). Soft agar colony assays were performed in triplicates and repeated thrice.

### mRNA Stability Assay

Cancer cell lines (300,000 per well) were grown in 6-well tissue culture plates to 60% confluency (48 hours), serum-free media was added for 24 hours and then grown in serum plus media for 12 hours. Treated with actinomycin D (10 µg/mL, 8 µmol/L) and cells were isolated at different time periods for RNA and protein expression analyses. For the mRNA stability assay, total RNA (1 µg) was used for cDNA synthesis using the Applied Biosystems High-capacity cDNA reverse transcriptase kit and qPCR was performed using Applied Biosystems primers as described previously (34–37). Differential expression was calculated using house-keeping gene *GAPDH* as control and percent mRNA expression was determined for the different time periods. Percent remaining RNA was used against timepoints to derive 50% expression half-life of mRNAs. Increased γH2AX expression was used as a measurement of actinomycin D activity. Percent mRNA remaining was plotted on GraphPad Prism against the number of hours after actinomycin D treatment and analyzed with the built-in linear regression function. The equations determined by the linear regression analysis are as follows: *Y* = −15.52*X + 100.0 (HeLa), *Y* = −9.815*X + 100.0 (ME180), *Y* = −4.530*X + 100.0 (HT3), *Y* = −6.341*X + 100.0 (SiHa), *Y* = −3.650*X + 100.0 (HeLa: CCND1), *Y* = −9.851*X + 100.0 (ME180:CCND1), and *Y* = −9.111*X + 100.0 (HeLa: *CDKN2A*). Half-life (t1/2) was calculated by determining the *X*-axis value (hours after actinomycin D treatment) for 50% RNA in *Y*-axis. At least two different qRT-PCR experiments, and each time in quadruplicates, were carried out for all the cell lines. The studies were done at least thrice.

### Proteosome and Autophagy Inhibitor Assay

Cells (1 × 10^6^ in 10 mm tissue culture dishes) were grown for 48 hours to 60% to 70% confluency, shifted to serum-free media for 24 hours and then to serum plus media for 24 hours. Autophagy inhibitor treatment with bafilomycin A (100 nmol/L) was carried out for 24 hours. Treatment with proteosome inhibitors MG132 and n-ethylmaleimide (10 µmol/L each) was performed for final 4 of 48 hours cell growth in serum plus media. Proteins isolated from the cells were subjected to SDS-PAGE analysis, transferred to nylan filters, and hybridized to antibodies using the established Western blot hybridization protocol (30, 38, 39). Inhibitor studies were done thrice for each experiment.

### Boyden Chamber Migration and Matrigel Invasion Assays

Migration and Matrigel invasion assays were performed in triplicates using 8 µmol/L filters in 24-well plates (33). Twenty-five thousand cells were seeded onto Boyden chamber cell culture inserts of 24-well plates and grown in 0.5 mL of 2% FBS-RPMI medium. The bottom wells of the chamber received 0.6 mL of 10% FCS as a chemoattractant. For the Matrigel assay, filters contained 100 µL of 300 µg/mL Matrigel, dried in tissue culture incubator for 2.5 hours and 25,000 cells (0.5 mL 2% FBS-RPMI medium) were gently layered on top of the Matrigel. Chemo attractant in the bottom well was 10% FBS-RPMI medium. After 24 hours incubation in the tissue culture incubator, cells in the top surface were wiped out with cotton swabs, cells at the bottom surface were fixed with 4% paraformaldehyde (z-fix) for 12 hours, washed twice with PBS for 5 minutes each time, stained with 0.4% crystal violet for 12 hours. After two washes for 5 minutes with PBS, cells were fixed with 70% ethanol for 15 minutes. The blue cells were photographed using Thermo Fisher scientific EVOS XL cell imaging system at 10X and 20X magnification settings and plotted as cells per observed field. The studies were done at least thrice.

### Lentiviral GAN Gene Transduction

GAN plasmid, a gift from Dr. Erik Lykken of Dr. Steven Gray's lab at University of Texas – Southwestern, Dallas, TX was used to amplify the full gigaxonin sequence (nt 1– 1794), and the product was cloned in the eukaryotic expressing vector JeT CMV-Myc. The derivative plasmid was digested with SAC II to get 1,840 bp fragment containing the GAN gene (cytomegalovirus promoter and Myc probing marker) and cloned into the SAC II site of pLEX-MCS lentiviral vector (Thermo Fisher Scientific). Transductin of lentiviral control vector and the vector containing the *GAN* gene preparations were performed using the established protocol. Lenti boost Kolliphor P338 (Sirion Biotech) was used at 1 mg/mL in the viral transductions. Clones were selected in puromycin (2 µg/mL) RPMI medium and single clones were isolated using cloning ring technique.

### RNA Isolation, Library Construction, and RNA-sequencing Gene Differential Analysis

RNAs were isolated from control or siRNA transfected cells using a pure Link RNA mini kit from Ambion (12183018A; Life Technologies, Inc.) and the RNA libraries were prepared using the KAPA mRNA HyperPrep kit (Roche) according to the manufacturer recommendations. The final libraries were pooled at equimolar concentration and sequenced on an Illumina NovaSeq6000 sequencer (SP lane, 2 × 50 bp). First, reads were demultiplexed and RNA sequencing (RNA-seq) data were processed to remove adapter sequences and low-quality reads. Then, raw reads were mapped to the human genome (hg38/GRCh38) with comprehensive genome annotation from Genocode (version 36) by the STAR ultrafast universal RNA-seq aligner (version 2.7.3a; refs. 40–42). Uniquely mapped reads were used by Htseq-count to calculate the number of reads (43). The reads were then normalized using DESeq2’s median of ratios method and DESeq2 (version 1.4.5) was used to perform differential gene expression analysis (43). For the Gene Ontology (GO), rpkm.csv data were used to identify 50 genes in each signature pathway. log-transform, differential mean value as Input Matrix Transformation, and Euclidean Distance (Complete Linkage) for sample and signature clustering metric were used.

### Whole Genome Library Preparation and Exome Sequence Analysis

Purified genomic DNA from 48 hours after serum starvation of ME180 and CRISPR-Cas9 derivative cell lines were quantified using Qubit dsDNA HS. Libraries were prepared using SparQ DNA Frag Library Prep kit (Quanta Bio) according to manufacturer's protocol. Briefly, 100 ng of DNA was fragmented and end-repaired for 15 minutes at 32°C followed by 30 minutes at 65°C. Ligation was performed using Illumina TruSeq Single Index Adapters (catalog no. 20015960 and 20015961 - Illumina). The final PCR was performed according to manufacturer's instructions for a total of five PCR cycles. Final libraries were sequenced as 1 × 50 on a HiSeq3000 (llumina; refs. 41, 42).

Genomic SNPs and insertions/deletions (InDel) variants were discovered using the GATK4 best practices workflows (https://gatk.broadinstitute.org/hc/en-us/sections/360007226651-Best-Practices-Workflows). Paired-end sequencing reads were aligned to the Homo sapiens genome assembly hg38 with the Burrows-Wheeler Aligner (BWA) program (43). Aligned sequencing output from BWA was sorted and converted to binary alignment map (BAM) using samtools version 1.9 (44). Optical and PCR duplicates in the aligned BAM were then marked using the Picard MarkDuplicates tool (http://broadinstitute.github.io/picard) and base quality score recalibration was performed and applied using the GATK BaseRecalibrator and ApplyBQSR tools. Variant calling was performed using the GATK HaplotypeCaller in GVCF mode followed by consolidation and genotyping using the GATK CombineGVCFs and GentotypeGVCFs tools (45). Variants were classified and split as SNPs or InDels with the GATK SelectVariants command and hard filtered with the parameters (QD < 2.0, QUAL < 30.0, SQR > 3.0, FS > 60.0, MQ < 40.0, MQRankSum < −12.5, and ReadPosRankSum < −8.0) for SNPs and (QD < 2.0, QUAL<30.0, FS > 200.0, and ReadPosRankSum < −20.0) for InDels. The variants passed filters were annotated with the Genetic variant annotation toolbox SnpEff (46). Variant allele frequency was calculated as the ratio of observed variant depth divided by the overall depth at the variant locus (47). Density of the variants was counted in 1 Mb interval across the genome using the bedtools 2.29.2 (48) and used for the creation of Circos plots. Circos plos were created using the RCircos Package (49) and chromosome plots were created using the ggplot2 library (50). Source code available on request.

### Mouse Xenograft and Tail Vein Injection Studies

Animal experimental studies were carried out with approval from the Institutional Animal Care and Use committee of the VAGLAHS. Cells (2 × 10^6^) were injected into the right flank for subcutaneous xenograft tumor formation or into the tail vein for lung metastasis studies (33, 51). Tumors were measured daily, and the volume was calculated using the formula Length × Width^2^/2, where length is the larger measurement. Animals were sacrificed when the control ME180 xenograft tumors reached a size of 1,000 mm3 or after 2 months for tail vein injected animals. Tumor tissues were fixed in z-fix (aqueous buffered zinc formalin, Anatech Ltd.) and 5 µm tissue slices were used for IHC studies.

### Statistical Calculations

Fisher exact test, two sided, was used for calculating significance of SNP to primary tumors. For the qRT-PCR, statistical analysis of differential expression was performed by one-way ANOVA with multiple pairwise comparisons with Sidak correction. All the studies including growth suppression studies were carried out at least in three independent experiments and statistical significance was calculated using the Student *t* test. All the results are presented as means ± SD and a *P* value of <0.05 was considered significant.

MTT assay, PCR and qRT-PCR studies, CRISPR-Cas9 Oligo preparation and Cloning, siRNA, Western Blotting, Immunoprecipitation, IHC, and Immunofluorescence methods are included in the Supplementary Data.

### Data Availability

All the data including those presented in Supplementary Materials and Methods, Tables and figures are available to other investigators. RNA and genome sequencing data are available in the following sites: CRISPR-Cas9 clone: RNA-seq submission: Gene Expression Omnibus Submission (GSE179424). CRISPR-Cas9 clone: Exome seq submission: SRA Submission (SRP327422). Data and code used to create Circos plots archived in UCLA Dataverse: https://doi.org/10.25346/S6/IMXOOK.

## Results

### Increased Frequency of the Exon 8 SNP (c.1293 C>T, rs2608555) in Primary Tumor versus Normal

The prevalence of GAN gene exon 8 SNP ranges from 8.1% in the East Asian population to 45.6% in the European population (15). This SNP has also been identified to varying degrees in human tumors in the COSMIC, DriverDB, and ICGC databases. However, the association to clinical phenotypes is not known. To determine the SNP frequency in primary tumors, 53 normal endometrial tissues adjacent to tumors, 70 head and neck, and 52 cervical cancers were studied. For 19 cervical cancer samples, we had adjacent normal endometrium. Rest of 34 normal DNAs were random samples derived from normal squamous epithelial cells. Ethnicity of the normal or tumor tissues were not known. RFLP analysis with the enzyme TspGW1 was performed using the PCR product of exon 8 primers (Supplementary Table S1). The SNP (T allele) is recognized by the presence of a 287 bp product while the wild-type (C allele) yields 155 and 132 bp products as shown in Supplementary Fig. S3A). Our analysis identified exon 8 SNP in 24.5% of normal samples, comparable to the frequency observed in the International HapMap Project (Supplementary Table S2). Tumor samples had 44.6% and 48% frequency in head and neck and cervical cancers, respectively. High frequency in tumor samples could be due to tumors belonging to subjects of European ethnicity, higher fraction treated at the three hospitals. There was no association to HPV in the tumor samples. However, there was a significantly higher SNP frequency in primary tumors versus normal tissues (13/53 normal vs. 56/122 tumors; *P* = 0.011). Analysis using Fisher two-sided comparison did not show a statistical significance to recurrence and metastasis (*P* = 0.171), to C/T or T/T alleles (*P* = 0.671 for C/T and *P* = 0.390 for T/T alleles (Supplementary Table S3). Both genomic (C/C, C/T, and T/T) and RNA expression (T vs. C allele) status of primary and recurrence/metastatic tumors including those of HPV positivity will be required to confirm and extend our findings on the relationship between gigaxonin expression and tumor development.

**FIGURE 1:**
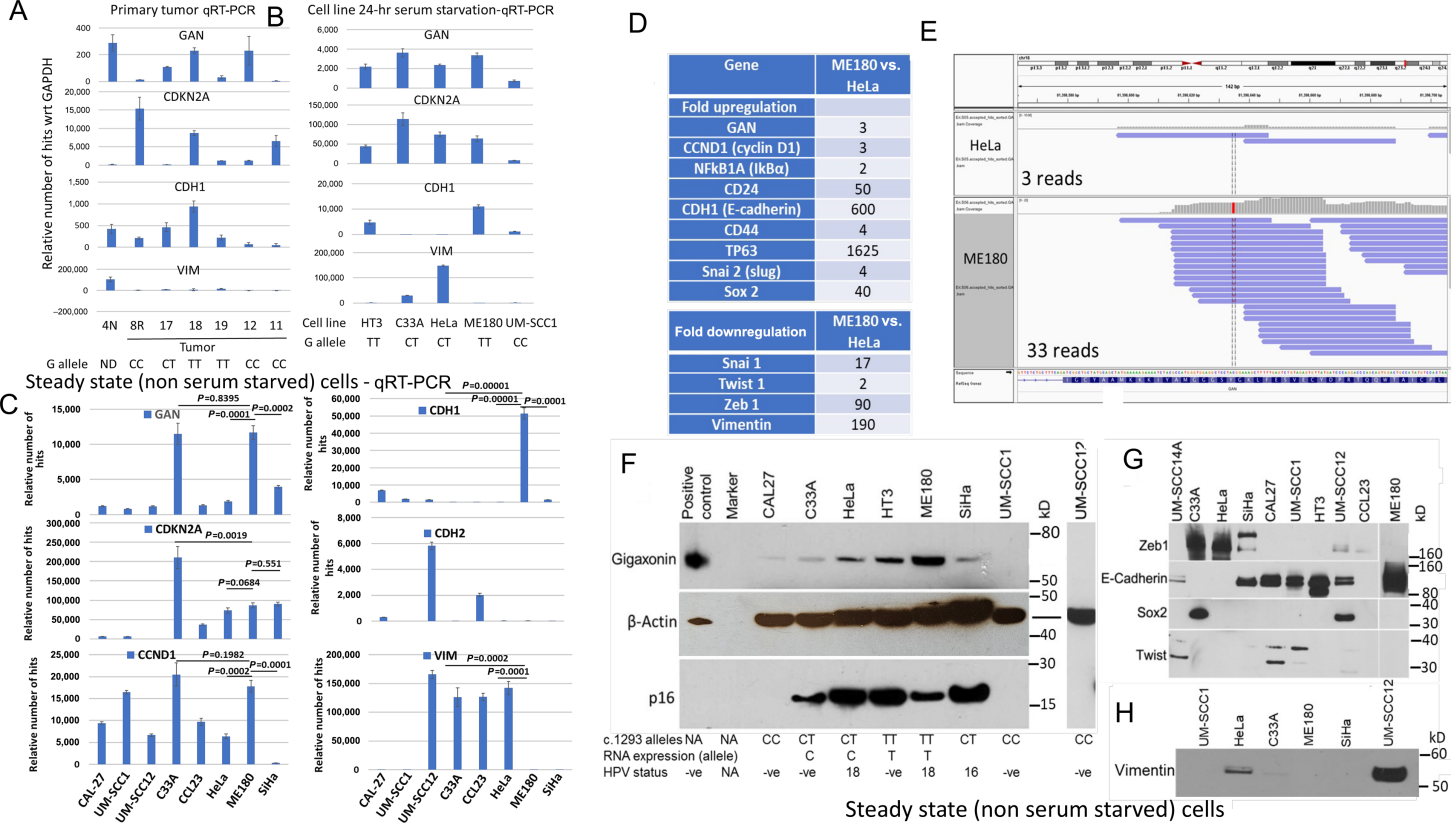
Association of exon 8 SNP to RNA and protein expression. Relative transcript hits are calculated from GAPDH transcript levels in HeLa cells (164,185 transcripts) and differential cell cycle (ΔCt) expression of *GAPDH* versus gene of interest of the qRT-PCR analysis. **A,** Normal endometrium (#4N) has higher GAN and vimentin expression, but reduced expression of *CDH1* (e-cadherin). Two (#17 and #18) of three primary tumors containing a T allele show higher *GAN* (gigaxonin) and *CDH1* (e-cadherin) expression. One tumor (#12) with CC alleles has higher gigaxonin, but reduced e-cadherin expression. A recurrence tumor (#8R) is positive for p16 but has low-level expression of gigaxonin and e-cadherin. Although preliminary, the results point to a direct relationship between gigaxonin and e-cadherin in primary tumor samples. Results indicate that gigaxonin expression is not correlating to p16 expression. **B,** HT3, C33A, HeLa, and ME180 cells express gigaxonin, but e-cadherin expression is seen only in HT3 and ME180 cells. Vimentin expression is observed in HeLa cells and at a reduced level in C33A. **C,** All cervical cancer cell lines and the head and neck cancer cell line CCL23 express *CDKN2A* (p16). Expression is absent in other head and neck cancer cell lines. Higher GAN expression is observed in ME180 and C33A cells. Expression of e-cadherin is seen only in ME180 cells. Low level expression of n-cadherin is seen in head and neck cancer cell lines CCL23 and UM-SCC12. While *CCND1* (cyclin D1) expression is observed in all cancer cell lines except for SiHa cells. Vimentin expression is seen in two each of cervical cancer (HeLa and C33A) and head and neck cancer (C33A and UM-SCC12) cell lines. **D,** RNA-seq data show 3-fold higher expression of GAN in ME180 in comparison with HeLa cells. Furthermore, RNA-seq data show statistically significant (*P* < 0.05) upregulation of e-cadherin *CDH1*, and *CD24* genes and downregulation of zeb1, and vimentin in ME180 in comparison with HeLa cells. **E,** Snap-shot view of ME180 versus HeLa shows 11-fold higher RNA transcripts in ME180 (T allele—red) pointing to RNA instability in HeLa cells due to C allele (blue) expression. Although genomic status of HeLa cells shows the presence of both C and T alleles, only C allele expression is seen at exon 8. Both qRT-PCR sequence and RNA-seq data show expression of T allele is absent in HeLa cells. **F,** Western blots show ME180 cells having highest gigaxonin expression. HT3 cells have higher expression than other cell lines containing C/T or C/C alleles. Reduced expression in CAL27 and absence of expression in UMSCC1 and UM-SCC12 cell lines are observed. Positive control used for gigaxonin protein size represents protein extract of FLAG tag *GAN* gene overexpressing Cos cells provided by Dr. Pascale Bomont of Lyon, France. While 10 µg of this protein extract was loaded, 40 µg of cell line protein lysates were loaded pointing to reduced β-actin expression in the positive control. Expression of CDKN2A is observed only in cervical cancer cell lines. **G,** Inverse relationship in the expression of e-cadherin and Zeb1 is seen in cancer cell lines. **H,** Higher expression of vimentin in HeLa, and UM-SCC12, and reduced expression in C33A cells are seen. Vimentin expression is absent in other cell lines. Same protein lysates were used for all the Western blots and thus a single β-actin blot is presented. UM cell lines refer to University of Michigan cell lines. *GAN*, gigaxonin; *CDKN2A*, p16; *CCDN1*, cyclin D1; *CDH1*, E-cadherin; *CDH2*, N-cadherin; *VIM*, vimentin; G allele, Genomic allele at codon 1293 of *GAN* gene.

qRT-PCR analysis of RNAs isolated from FFPE samples available from one normal endometrium and six head and neck cancers were performed and differential cycle numbers with respect to *GAPDH* (ΔCt values) was used to calculate relative transcript levels using HeLa cell (although it is a tumor cell line control) *GAPDH* RNA-seq numbers (164,185 transcripts) as control. The results showed higher gigaxonin expression in two of three cancers (#17 and 18) containing *GAN* gene exon 8 T allele in their genome (Fig. 1A). There was higher expression of *CDH1* (e-cadherin, a representative marker of epithelial cell phenotype) in these two primary tumors. A third primary tumor (#19) containing exon 8 T/T alleles had reduced gigaxonin and e-cadherin expression. The discordance between the presence of T allele and absence of gigaxonin expression may be related to RNA instability through other mutational events. One of the cancers (#8R), a recurrence tumor, containing C alleles was positive for *CDKN2A* (p16) by qRT-PCR and IHC. However, this tumor showed decreased expression of gigaxonin and e-cadherin. Of the two other tumors with C alleles, one of them (#12) had higher gigaxonin expression, but no e-cadherin expression. The other tumor (#11) was p16 positive but the expression of gigaxonin, and e-cadherin was absent. Although the results are preliminary, our data pointed to a direct relationship between the presence of T allele and expression of gigaxonin and e-cadherin in primary head and neck cancer.

### Presence of Exon 8 SNP Correlates with Higher Expression of Gigaxonin in ME180 (HPV18-Positive) and HT3 (HPV-Negative) Cell Lines

To determine the association between *GAN* gene exon 8 SNP and gigaxonin expression, we analyzed five head and neck squamous cell carcinoma (HNSCC) and seven cervical cancer cell lines. The neuroblastoma cell line LAN6 was used as the HPV-negative control. These cell lines were authenticated using mitochondrial DNA sequencing and cellular morphology (Supplementary Figs. S1 and S2). The exon 8 genomic analysis revealed homozygous SNP (T/T) alleles in two cervical cancer cell lines (ME180 and HT3), heterozygous (C/T) alleles in four cell lines (three cervical cancer and one HNSCC) and homozygous wild-type (C/C) alleles in the other seven cell lines (Supplementary Table S4). Sequencing of the PCR and RT-PCR products derived using genomic and exon specific primers confirmed the presence of T allele in the six cell lines (two with T/T and four with C/T alleles). We did not find missense or stop codon mutations in any of the cancer cell lines.

RT-PCR and qRT-PCR were performed on the cell lines using genome specific primers (Supplementary Table S5). Again, differential cycle numbers with respect to *GAPDH* (ΔCt values) was used to calculate relative transcript levels using HeLa cell (tumor cell line control) *GAPDH* RNA-seq numbers (164,185 transcripts) as control. In addition to gigaxonin we focused our attention on the expression of *CDKN2A* (p16) and *CCND1* (cyclin D1) as we have shown inverse relationship between the two proteins in our studies on head and neck cancer (28). Expression of e- and n-cadherins shown in epithelial versus mesenchymal phenotype and vimentin shown to be ubiquitinated by gigaxonin (3) were also studied. Analysis performed on RNAs from 24 hours after serum starved cervical cancer cell lines HT3, C33A, HeLa, ME180, and head and neck cancer cell line UM-SCC1 showed higher gigaxonin expression in all four cervical cancer cell lines (Fig. 1B). The cervical cancer cell lines were also positive for CDKN2A (p16 expression). However, HT3 and ME180 containing T alleles are the only cell lines with higher *CDH1* (e-cadherin) expression pointing to a direct association between gigaxonin and e-cadherin expression. Vimentin expression was higher in HeLa cells. The results from the steady state (no serum starvation—heterogenous cell population) cell line samples showed CDKN2A expression in cervical cancer cell lines and a head and neck cancer cell line, CCL23 (Fig. 1C). There was higher *GAN* (gigaxonin) expression in ME180 and C33A cells and reduced expression in all other cell lines. Expression of e-cadherin was observed only in ME180 cells. Expression of mesenchymal marker n-cadherin was seen at a low level in two head and neck cancer cell lines, UM-SCC12 and CCL23. While higher *CCND1* (cyclin D1) expression was seen in all the cell lines except SiHa, Vimentin was expressed in two each of cervical cancer (HeLa and C33A), and head and neck cancer (UM-SCC12 and CCL23) cell lines.

Time course expression analysis of *CDKN2A* and *GAN* at 0, 6, and 24 hours after a 24-hour serum starvation was performed to measure the relationship between SNP and stability of GAN transcripts during cell cycle. Fibroblast cell line GM05399 was used as normal cell line cell cycle control and *GAPDH* was used as housekeeping gene control. Similar pattern of *CDKN2A* expression, higher at 6 hours and reduced at 24 hours was seen in all four cervical cancer cell lines (Supplementary Fig. S3B). However, gigaxonin expression was stable in ME180 and HT3 in comparison with HeLa and C33A cell lines (Supplementary Fig. S3C). Expression of both CDKN2A and gigaxonin was not detected in head and neck cancer cell lines UM-SCC1 and UM-SCC14A.

RNA-seq analysis of ME180 and HeLa cell lines showed a 3-fold increased gigaxonin expression in ME180 in comparison with HeLa cells (Fig. 1D). The data further showed statistically significant (*P* < 0.05) upregulation of e-cadherin (*CDH1*), and CD24 genes and downregulation of Zeb1, and vimentin in ME180 in comparison with HeLa cells. Snapshot view (IGV: interactive genome browser) of exon 8 SNP area of RNA-seq showed T allele expression in ME180 and C allele in HeLa cells (Fig. 1E). There was an 11-fold increase in transcript reads (33:3) in ME180 in comparison with HeLa cells. Although both C and T alleles were present in the HeLa cell genome (Supplementary Table S4), expression was seen only for the C allele (vertical line in Fig. 1E). T allele expression was not observed pointing to lesser exon 8 expression in HeLa cells when the expression was from the C allele.

Western blot protein analysis showed highest expression of gigaxonin in ME180 cells containing T/T alleles (Fig. 1F). There was higher expression, but less than that of ME180 in HT3 cells. Cell line C33A is a faster growing cell line compared with HT3 both in the MTT and soft agar colony forming assays (Supplementary Fig. S4A–S4C). Although C33A cells had a similar GAN transcript level of ME180 in both the serum starved and non-serum starved steady state level conditions (Fig. 1B and C), protein expression was reduced because of expression of the C allele in the RNA sequence (Supplementary Fig. S4D). There was reduced expression in cell lines containing C/T alleles and minimal or absence of expression in C/C allele containing HNSCC cell lines. As observed in the RNA-seq, there was increased expression of e-cadherin in ME180 cells while the expression was absent in HeLa and C33A cell lines (Fig. 1G). SiHa, and head and neck cancer cell lines had reduced e-cadherin expression with the least expression in UM-SCC14A cells. High expression of Zeb1 was observed in HeLa and C33A cells and there was reduced expression in SiHa and UM-SCC12 cells. There was minimal or no Zeb1 expression in other cell lines. There was expression of Sox2 and Twist1 in some cell lines. Vimentin expression was highest in UM-SCC12 cell lines and a reduced expression in HeLa cells (Fig. 1H). Other cell lines, including ME180 and HT3, did not show vimentin expression.

### Inverse Relationship Between Gigaxonin Expression and *In Vitro* Cell Growth

**FIGURE 2:**
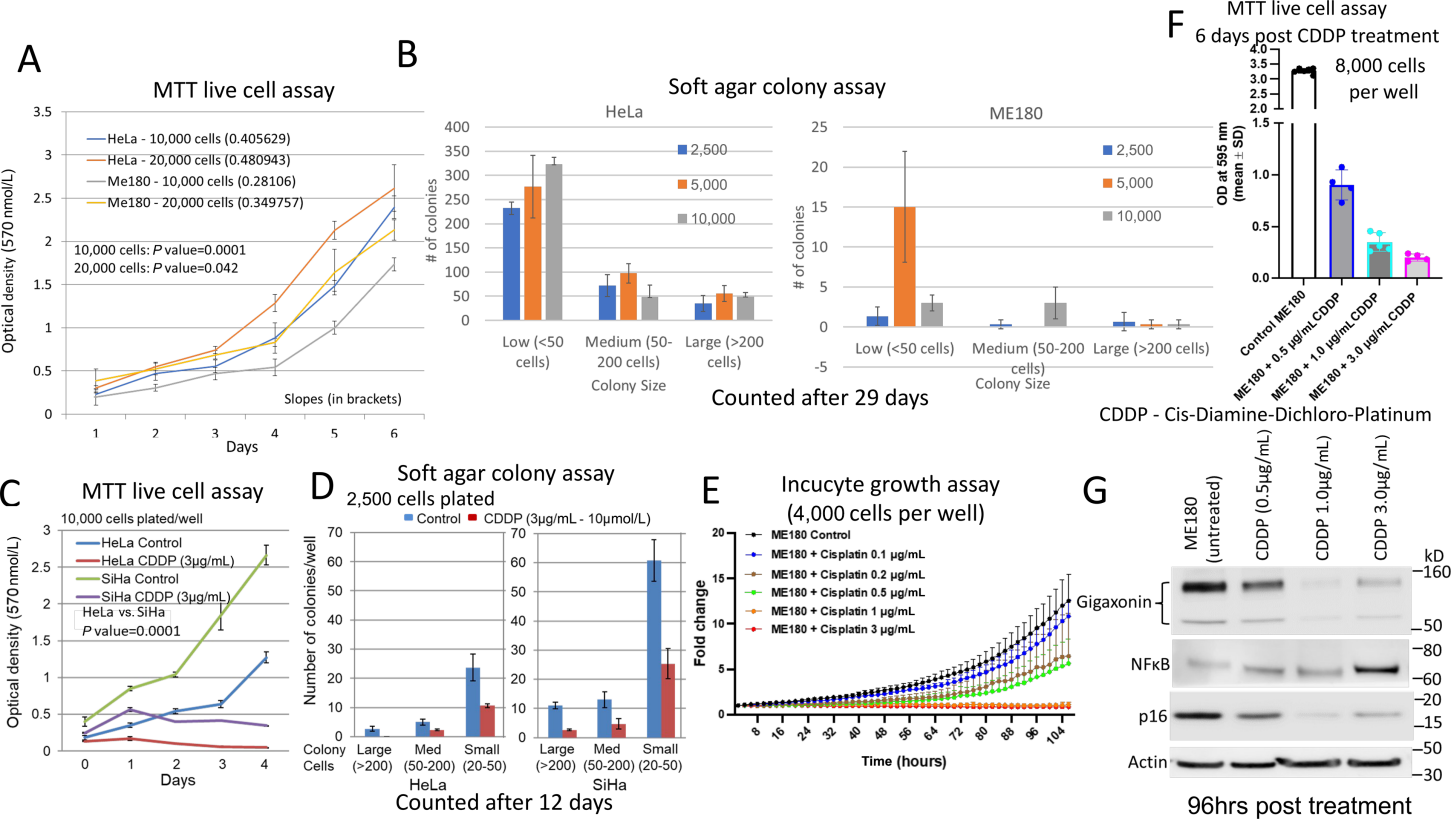
Inverse relationship between gigaxonin expression and cancer cell line growth in HPV18 and HPV18- versus HPV16-positive cell lines. **A,** Statistically significant reduced *in vitro* cell growth is seen in ME180 in comparison with HeLa cells. **B,** Soft agar colony formation assay confirms reduced growth of ME180 cells. Although HeLa and SiHa cell lines contain C/T alleles, HeLa cells with higher gigaxonin expression than SiHa cells. **C,** Shows *in vitro* slower growth. **D,** Reduced soft agar colony formation and increased cisplatin sensitivity. **E,** Incucyte growth assay demonstrates increasing sensitivity of ME180 cells with increasing concentration of cisplatin. Cell images acquired during the Incucyte assay shows presence of live cells 72 hours after cisplatin treatment (Supplementary Fig. S6). **F,** MTT assay demonstrates presence of live cells 6 days after cisplatin treatment (30%, 10%, and 6% live cells compared with untreated control in 0.5, 1.0, and 3.0 µg/mL of cisplatin treatment, respectively). **G,** Cells collected after 4 days of cisplatin treatment show decreased expression of GAN, and p16 (CDKN2A) and enhanced NFκB expression. The data suggest that NFκB activation is related to the growth of cisplatin-resistant cells.

To examine the association between gigaxonin expression and cell growth, MTT and soft agar colony formation assays were performed. HeLa and ME180 contain HPV18, HeLa and SiHa contain HPV18 and HPV16 sequences, respectively. Studies were performed in all three cell lines at the same time. Because of differential growth of SiHa (higher growth) and ME180 (slower growth), data are presented as ME180 versus HeLa and HeLa versus SiHa cells. ME180 cells showed statistically significant (*P* < 0.0001 for 10,000 cells and 0.042 for 20,000, cells respectively) reduced cell growth and soft agar colony formation in comparison with HeLa cells (Fig. 2A and B). Representative soft agar colonies are shown in Supplementary Fig. S5A. The data further revealed reduced cell proliferation and increased cisplatin sensitivity of HeLa cells in comparison with SiHa cells (Fig. 2C and D; Supplementary Fig. S5B). Our results therefore indicated that HPV18 containing cells are less aggressive and more sensitive to cisplatin than HPV16 containing cervical cancer cell lines.

To further identify the relationship of gigaxonin expression to cisplatin sensitivity, ME180 cells with the highest gigaxonin expression was treated with 0.1 to 3.0 µg/mL (0.34 to 10 µmol/L concentration) of cisplatin, and cell growth and protein expression were measured. The Incucyte (Real-time Cell Imager) growth assay showed increased sensitivity with increased cisplatin concentration (Fig. 2E). Cell images acquired during Incucyte growth assay showed presence of live cells 72 hours after 1.0 and 3.0 µg/mL of cisplatin treatment (Supplementary Fig. S6). MTT assay performed 6 days after cisplatin treatment confirmed the presence of live cells in cisplatin-treated cells (Fig. 2F). Protein expression analysis of cisplatin-treated cells showed upregulation of NFκB at 3.0 µg/mL cisplatin correlating to the development of cisplatin resistance (Fig. 2G). There was downregulation of p16 and gigaxonin expression correlating to the inverse relationship between the two proteins to NFκB and sensitivity to cisplatin. While we have observed an inverse relationship between *in vitro* cell growth and GAN gene exon 8 SNP, other intrinsic properties of each cell line could play a role in the differential phenotype of cancer cell lines.

### CRISPR-Cas9–mediated Conversion of Exon 8 SNP (T/T) to Wild-type C/C Alleles Results in Reduced Gigaxonin Expression, Increased *In Vitro* Cell Growth and Enhanced Epithelial–mesenchymal Transition Marker Expression

**FIGURE 3:**
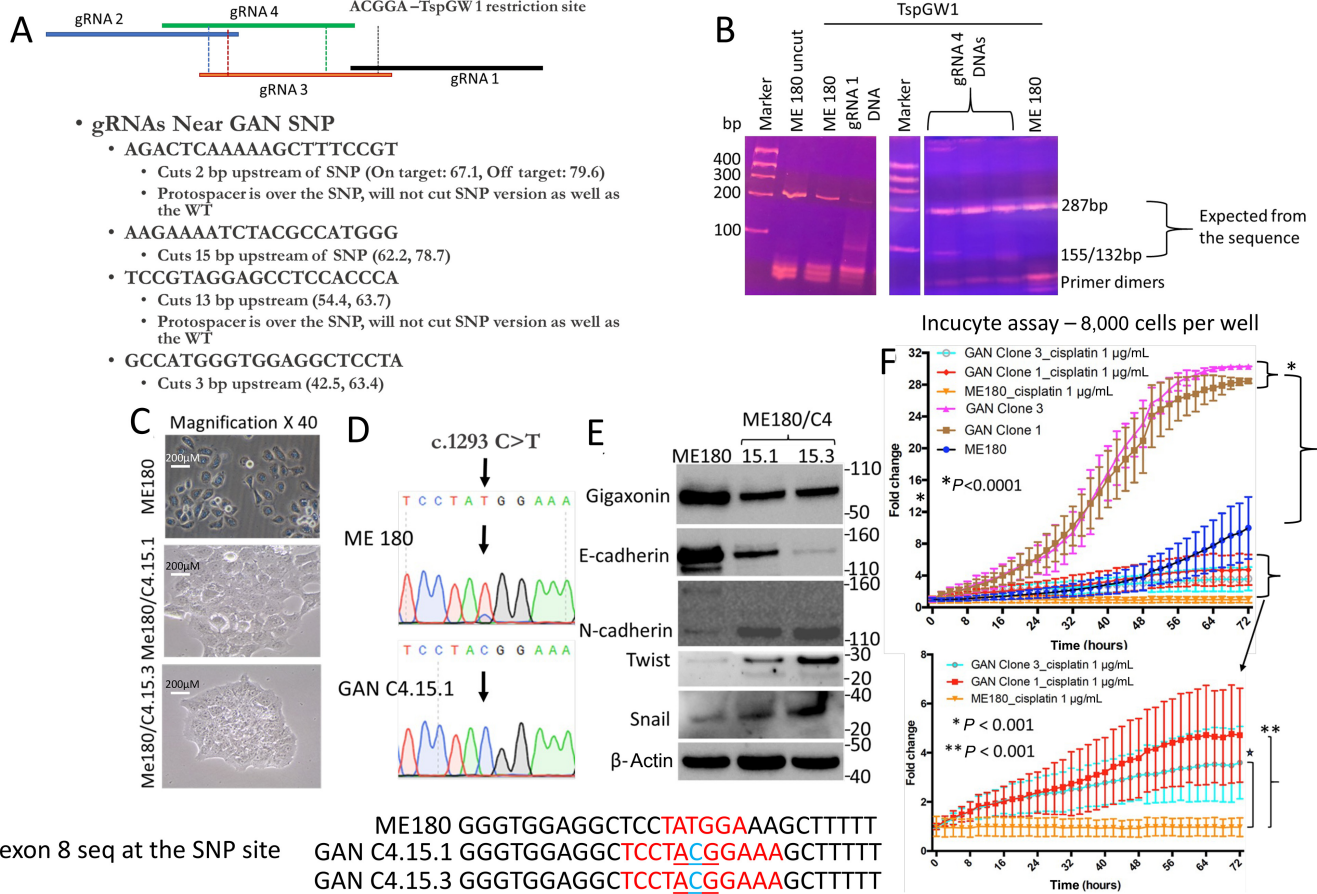
Enhanced *in vitro* cell growth and higher expression of EMT markers in GAN edited clones. **A,** Map of the gRNAs used in CRISPR-Cas9 assay. **B,** TspGW1 digested DNAs of puromycin-resistant clones of gRNAs 2 and 4 transfected ME180 cells show the presence of 287 and 155/132 bp PCR products (T and C alleles, respectively) compared with the presence of 287 bp (T/T alleles) in the parental ME180 cells. **C,** Representative morphology of *GAN* edited clones of gRNA4, C4.15.1, 15.3 in comparison with ME180 cells. **D,** Exon 8 PCR product sequencing shows conversion of T/T with C/C alleles in the *GAN* edited clone C4.15.1. Sequencing data show exon 8 SNP site containing T allele in ME180 and C allele in both the GAN edited clones C4.15.1 and C4.15.3. **E,** Western blot data show decreased expression of gigaxonin and e-cadherin in these clones in comparison with ME180 cells. However, there is increased expression of EMT markers, Snail, Twist, and n-cadherin in GAN edited clones. **F,** Incucyte growth assay shows faster growth of GAN edited clones C4.15.1 and C4.15.3 in comparison with parental ME180 cells. Cisplatin sensitivity is also reduced in these clones.

To explore the relationship further, we decided to convert T/T alleles of ME180 cells to C/C alleles using the CRISPR-Cas9 system. We prepared four different guide RNAs (gRNA) containing PAM sequences for the exon 8 SNP site (Fig. 3A; Supplementary Table S6). Exon 8 PCR products of the DNA were subjected to TspGW1 restriction enzyme analysis to identify the conversion of T to C allele (Fig. 3B). Puromycin-resistant single-cell clones were isolated using antibiotic selection and non-selection for 2 to 3 weeks. Clones of gRNA 1, C1.2, C1.2, and C1.3 had similar morphology to ME180 with the retention of the 287 bp exon 8 PCR product indicating the retention of the SNP (Supplementary Fig. S7). These clones were not pursued further. However, gRNA 4 clones C4.15.1, and C4.15.3 showed a flat cell-cell adherent mesenchymal cell morphology with low light transmission (Fig. 3C). Presence of C/C alleles in in these CRISPR-Cas9 clones (now on called *GAN* edited clones) was seen in the exon 8 PCR products (155/132 bp product in TspGW1 restriction enzyme analysis) which was confirmed by DNA sequencing (Fig. 3D).

Western blot analyses showed decreased gigaxonin expression in GAN edited cells indicating a direct relationship between T>C conversion and gene expression (Fig. 3E). Zeb1 expression was observed in cervical cancer cell lines HeLa, C33A, and SiHa, while it was absent in ME180 cells. We therefore searched for other epithelial–mesenchymal transition (EMT) markers and found ME180 cells to express Snail and Twist at basal levels and focused on these two proteins (Snail in later experiments) and cadherins in ME180 and GAN edited clones. There was enhanced expression of *Snail*, *Twist1*, and n-cadherin accompanied by reduced e-cadherin expression in the *GAN* edited clones in comparison with parental ME180 cells (Fig. 3E). Incucyte growth assay showed faster growth and increased resistance to cisplatin of GAN edited clones in comparison with ME180 cells (Fig. 3F).

### Reversal of EMT Marker Phenotype with Lentiviral-mediated Re-expression of Gigaxonin in GAN Edited Cells

**FIGURE 4:**
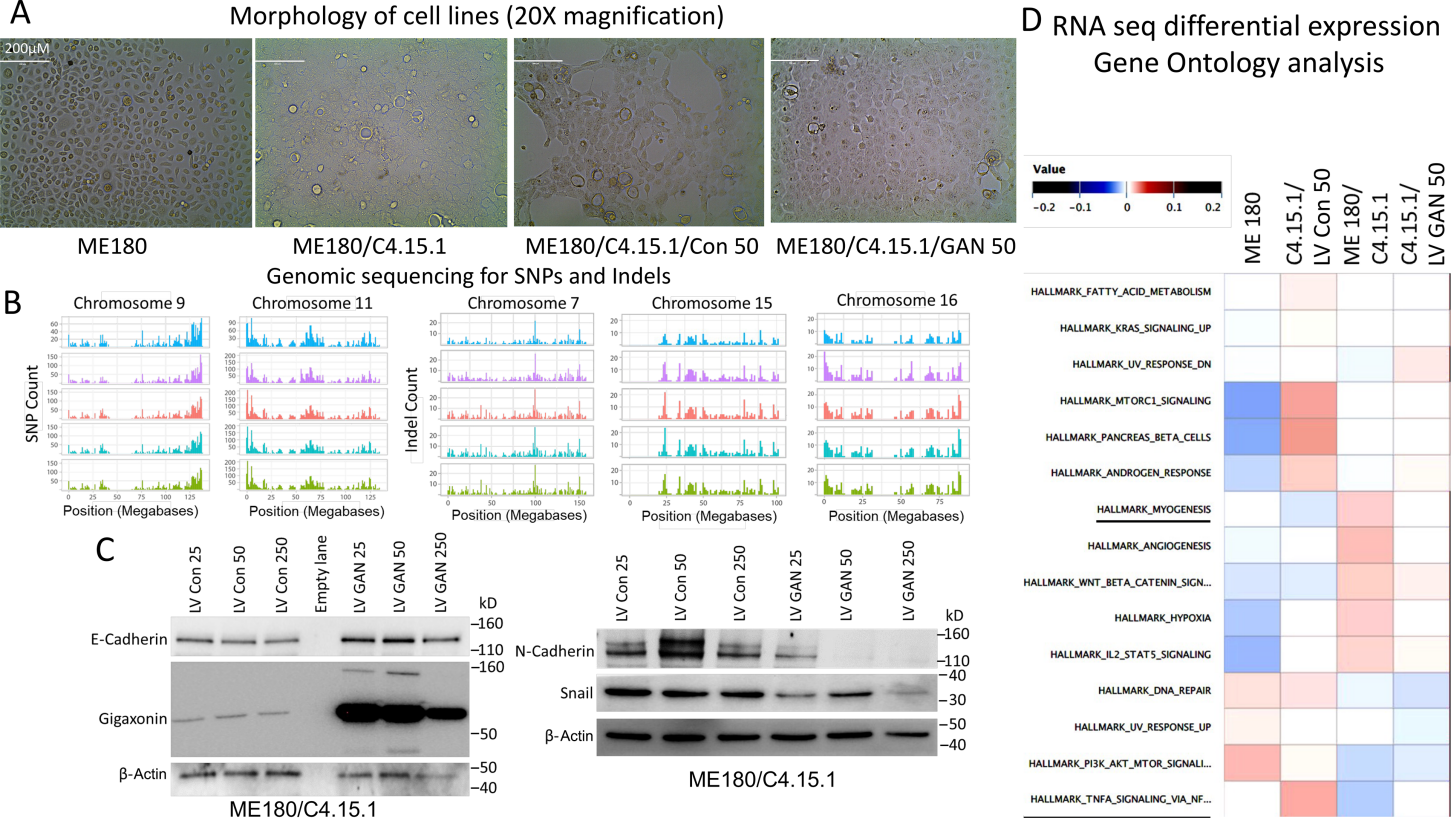
Re-expression of gigaxonin leads to reversal of NFκB pathway. **A,** Re-expression of gigaxonin in GAN edited cells showed an intermediate epithelial morphology with a brighter light transmission than GAN edited cells or control lentiviral clones. **B,** Circos plots of the exome sequence analysis showing similar peaks for SNP in chromosomes 9 and 11 and intrachromosomal deletions (indels) for chromosomes 7, 15, and 16 in the parental ME180, GAN edited, and the lentiviral transfected clones. **C,** Re-expression of gigaxonin leads to increased expression of epithelial marker e-cadherin. Loss of n-cadherin and Snail expression are seen in gigaxonin re-expressing cells confirming inverse relationship between the gigaxonin and EMT markers. **D,** GO program of the RNA-seq data shows reversal to myogenesis signaling, and *TNFα* signaling via *NFκB* pathways in GAN re-expressing ME180/C4.15.1/LV GAN 50 cells. Lentiviral aliquots 25, 50, and 250 represent transfection of 5 × 10^3^, 1 × 10^4^, and 5 × 10^4^ viral particles.

To validate the relationship between gigaxonin expression and tumor cell growth, we reintroduced lentiviral cloned GAN gene (Supplementary Fig. S8) using an established transduction protocol. A clone each of the control (LV Con 25, 50, and 250) and *GAN* (LV GAN 25, 50, and 250) transductions were analyzed. The aliquots 25, 50, and 250 correspond to 0.5 × 10^4^, 1 × 10^4^, and 2.5 × 10^4^ lentiviral particles, respectively. Re-expression of gigaxonin in GAN edited cells showed an intermediate epithelial morphology with a brighter light transmission than GAN edited cells or control lentiviral clones (Fig. 4A).

Exome sequence analysis of DNA samples from the control vector and GAN virus transductions (50 µL aliquot corresponding to 1 × 10^4^ virus particle transfected cells) showed identical peaks for chromosomal deletions (del plot), interchromosomal deletions (indel plots), and SNP plot in ME180, GAN edited and control or GAN vector transfected cells (Fig. 4B; Supplementary Figs. S9 and S10). The circos plots of the del, indel, and SNPs further demonstrated that GAN edited cells were derived from the parental ME180 cells (Supplementary Fig. S11A–S11C). Similarly, the lentiviral transfected cells were identical in genetic composition to the *GAN* edited C4.15.1 cells. The non-synonymous mutation analysis showed unique peaks in ME180 cells that were absent in other cell lines. A Venn diagram showed that two-thirds of sites (5,369 sites) were identical in all four cell lines and one-third (3,861 sites) was unique to ME180 (Supplementary Fig. S11D). Thus, the *GAN* edited cells were mostly identical for synonymous and non-synonymous chromosomal regions to the parental ME180 cells. *GAN* clone isolation from single-cell cloning through puromycin selection and non-selection and the isolation of cells with a morphology different from that of parental ME180 cells could explain genomic differences between the parental ME180 cells and the GAN clone ME180/C4.15.1. The difference between the GAN clone and lentiviral control or GAN lentiviral transfected cells was identical in (8,878 sites Supplementary Fig. S11D). Only five sites were unique to GAN edited ME180/C4.15.1 cells and 13 and 9 unique sites were present in control and GAN lentiviral transfected cell lines, respectively. Western blot studies revealed enhanced expression of gigaxonin and e-cadherin in all the GAN re-expression cells compared with control virus transfected clones (Fig. 4C). We also found decreased expression of Snail and n-cadherin indicating reversal of the EMT phenotype in gigaxonin re-expressing cells (Fig. 4C).

Differential expression analysis of the RNA-seq confirmed gigaxonin expression in GAN lentiviral transfected cells and partial reversion of genes of which SerpinB2 codes for a protein involved in senescence and autophagy (Supplementary Fig. S12). GO analysis data showed near reversal to ME180 signaling pathways including that of myogenesis and TNFα signaling via NFkB with the re-expression of gigaxonin (Fig. 4D; Supplementary Fig. S13). Thus, the RNA-seq data confirmed our earlier report on the effect of gigaxonin on NFκB (30) and suggested that NFκB might mediate downregulation of Snail. Both RNA sequence and Western blots suggested that many of the ME180 phenotypes are reversible with re-expression of gigaxonin.

### Gigaxonin Re-expression Inhibits *In Vitro* Cell Proliferation and Matrigel Invasion

A snapshot view of the exon 8 RNA-seq data pointed to high expression of gigaxonin in ME180 and GAN lentiviral transfected cells (Supplementary Fig. S14). Red vertical bar indicating T allele expression correlating to higher gigaxonin expression was seen in ME180 cells. Deletion of exon 8 sequences (red horizontal lines) was observed in GAN edited and control lentiviral transfected cell lines.

**FIGURE 5:**
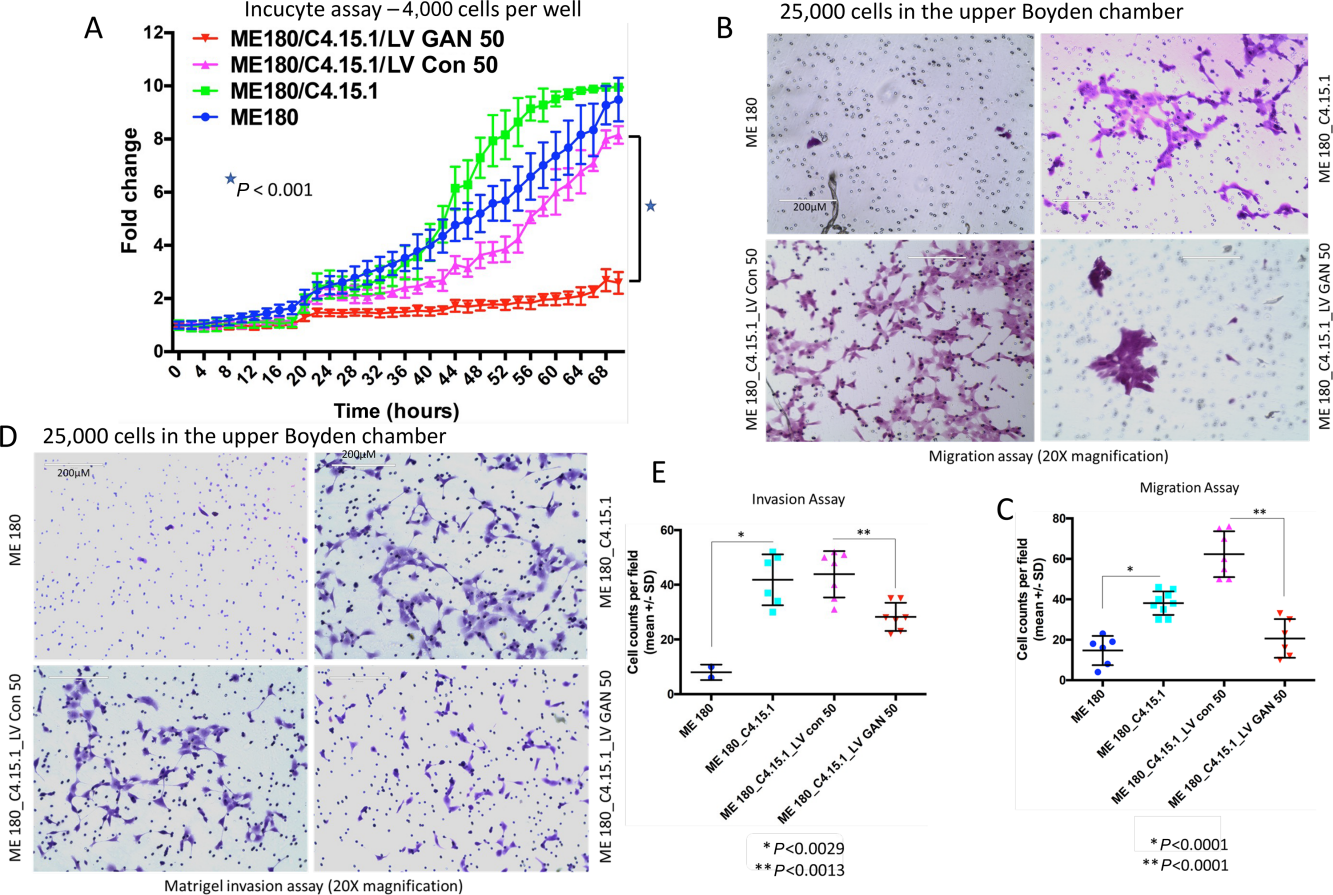
Gigaxonin re-expression leads to reversal of EMT phenotype. **A,** Incucyte growth assay shows gigaxonin re-expressing cells with statistically significant (*P* < 0.001) slower growth than control lentiviral transfected cells. Differential growth between parental GAN edited cells and that of control lentiviral transfected cells is not statistically significant (*P* = 0.1088). **B** and **C,** Migration assay shows minimal migration of ME180 cells. There is statistically significant (*P* < 0.0001) increased migration of *GAN* edited and control viral transfected cells which is reduced with the re-expression of gigaxonin. **D** and **E,** Invasion through Matrigel again shows absence of invasion in ME180 cells that dramatically increases in GAN edited cells. Gigaxonin re-expressing cells show statistically significant (*P* < 0.0013) reduced Matrigel invasion in comparison with the control viral transfected cells.

Furthermore, the Incucyte growth assay showed statistically significant reduced growth of GAN re-expressing cells in comparison with control lentiviral vector cells (*P* < 0.001, Fig. 5A). The reduced growth seen in control vector transfected cells in comparison with that of parental GAN edited cells was not significant (*P* = 0.1088). Because GAN edited cells migrated through soft agar and did not form recognizable colonies, we performed migration and Matrigel invasion assays using 8 µmol/L cell culture filters. The analysis showed statistically significant increased migration (*P* < 0.0001) and Matrigel invasion (*P* < 0.0029) of GAN edited cells in comparison with ME180 cells (Fig. 5B–E). GAN lentiviral transfected cells showed statistically significant reduced migration (*P* < 0.0001) and Matrigel invasion (*P* < 0.0013) in comparison with control vector transfected cells. Control lentiviral cells had a higher migration rate, but a similar Matrigel invasion to that of the GAN edited cells.

To confirm that loss of gigaxonin expression leads to aggressive cell growth, another protocol, that of siRNA transfection was performed. GAN siRNA transfected cells had reduced gigaxonin expression and statistically significant increase in migration (*P* < 0.0014) and Matrigel invasion (*P* < 0.0008) in comparison with untreated, liposome alone and control siRNA treated ME180 cells (Supplementary Fig. S15A–S15E). From both the CRISPR-Cas9 editing and siRNA transfection studies, we have confirmed that the loss of gigaxonin expression leads to aggressive *in vitro* cancer cell line growth and growth suppression occurs with the re-expression of gigaxonin.

Biochemical studies showed that siRNA transfected cells have increased expression of NFκB and Snail with the loss of gigaxonin expression. Gigaxonin reexpression through GAN cDNA showed reduced expression of NFκB and Snail (Supplementary Fig. S16A). Because HeLa cells do not express Snail, but express Zeb1, we re-expressed gigaxonin through GAN cDNA. There was reduced Zeb1 expression indicating downregulation of EMT markers by gigaxonin (Supplementary Fig. S16B).

### Reduced Mouse Lung Metastasis with the Re-expression of Gigaxonin

**FIGURE 6:**
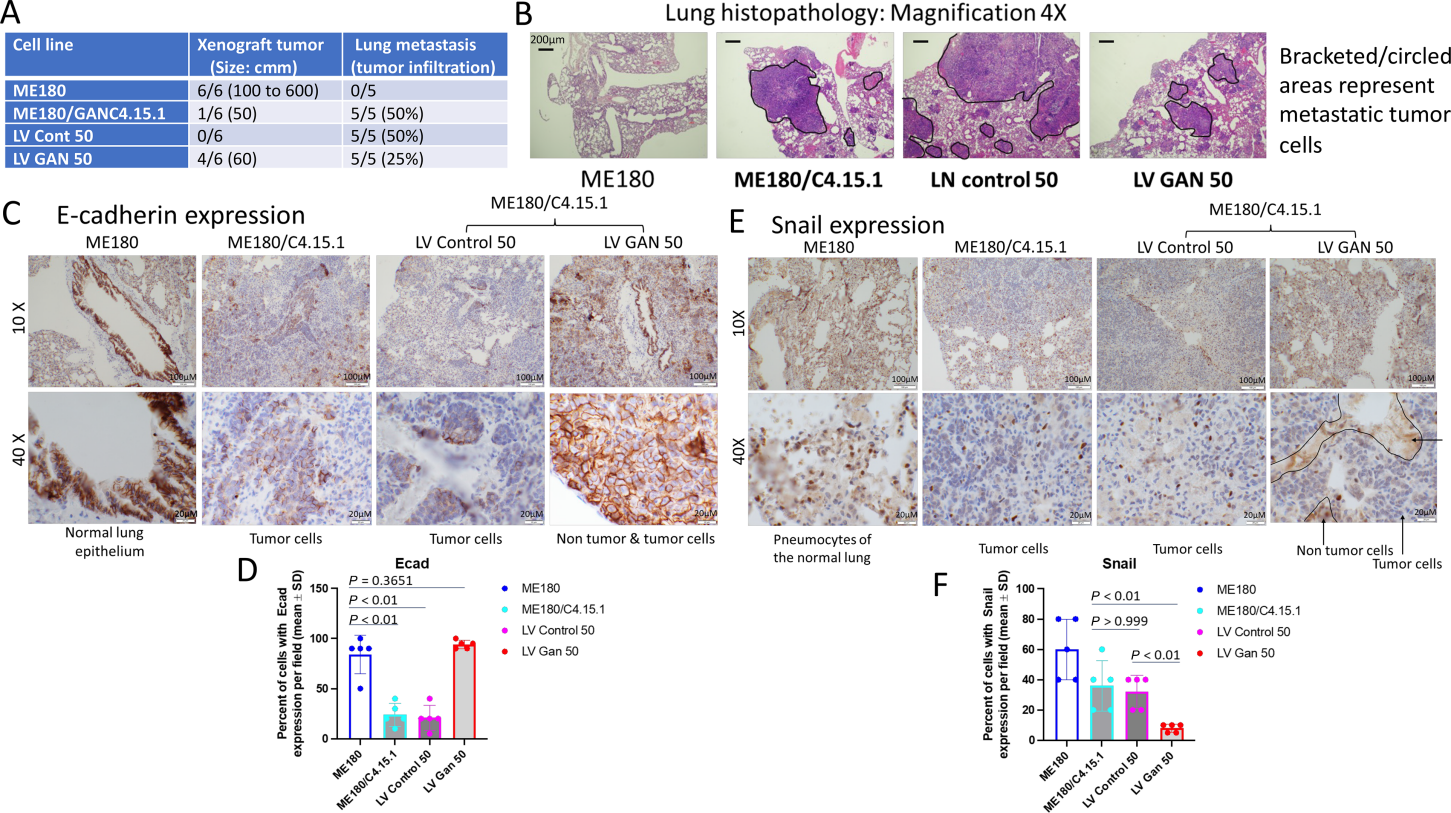
Inhibition of mouse lung metastasis with re-expression of gigaxonin. **A,** Table shows xenograft tumors formation by ME180 cells and lung metastasis by GAN edited clones. ME180 did not form lung metastasis. Greater than 50% reduced metastasis was seen in GAN lentiviral transfected cells compared with untransfected and control lentiviral transfected cells. **B,** H&E staining shows absence of tumor cells in the lung of parental ME180 cells after tail vein injection indicating absence of lung metastasis. While high metastasis (tumor cells infiltrating >50% of the lung, cells, bracketed and circled areas) is seen with *GAN* edited and control lentiviral transfected cells, a 50% reduction in metastasis (black circles) compared with the control virus transfected cells is observed in GAN lentiviral transfected cells. **C** and **D,** Statistically significant expression (3+, >90%, *P* < 0.01) of e-cadherin is seen in the epithelium of normal lung tissue after ME180 tail vein injection. GAN edited cells and control lentiviral transfected cells show low level expression (1+, 40%) in the lung metastatic tumor cells. *GAN* lentiviral transfected cells have higher expression (3+, >80%) in tumor cells (*P* < 0.01). **E** and **F,** The pneumocytes of normal lung tissue of ME180 injected mice shows nuclear Snail expression serving as an internal positive control. Lung tumors of GAN edited, and control lentiviral cell injected mice show higher nuclear expression. However, there is statistically significant reduced nuclear expression (*P* < 0.01) in GAN lentiviral injected lung tumor cells. The non-tumor cells of this sample (indicated by arrows) have weak cytoplasmic expression. Thus, an inverse relationship in the expression of e-cadherin and Snail is seen in gigaxonin overexpressing cells versus the GAN edited cells indicating a reduction in metastasis related to higher gigaxonin expression. Thicker arrows point to non-tumor cells and the thinner arrow points to tumor cells.

To identify the effect of gigaxonin on *in vivo* tumor growth, ME180, GAN edited and gigaxonin re-expressing cell lines were used for mouse subcutaneous xenograft tumor formation. Injection of ME180 cells (2 × 10^6^ cells per mouse) underneath the skin of immune compromised nude mice (NSG mice) showed xenograft tumor formation in 6 of 6 mice (Fig. 6A; Supplementary Fig. S17). Tumors grew to a size of 100 to 600 cmm in 4 weeks. Small tumors (60 cmm size) were seen in 4 of 6 mice with GAN lentiviral transfected cells. GAN edited cells formed a small tumor (50 cm) in one mouse. Tumor growth was not observed in the other 5 mice and in all 6 of 6 mice of control lentiviral transfected cells. Absence of tumor formation in GAN edited and control lentiviral transfected cells indicated that the cells did not attach to the skin.

To explore the possibility of *in vivo* invasion and metastasis, tail vein injections (2 × 10^6^ cells per mouse) were carried out in NSG mice, and the mice were sacrificed after 2 months. Lung, liver, and colon were examined by hematoxylin and eosin (H&E). There was no liver or colon metastasis in any of the animals. While there were tiny tumors at the base of the tail (may be from a fraction of cells attaching to inner endothelial cell wall), there was no lung metastasis in ME180 cells (Fig. 6B). GAN edited and control vector transfected cells formed metastatic lung tumors. There was >50% infiltration of tumor cells into the lung in these two cell lines. However, the GAN lentiviral transfected cells showed reduced (>50% reduction in comparison with the control lentiviral cells) metastasis. These results showed that ME180 cells expressing higher gigaxonin (through exon 8 T allele) did not form distant metastasis. GAN edited cells with reduced gigaxonin expression correlated with the development of metastasis. Re-expression of the GAN gene leads to reduced metastasis indicating gigaxonin as a metastasis suppressor protein.

To determine the relationship between EMT and lung metastasis, paraffin fixed lung tissue slides were hybridized to e-cadherin and Snail antibodies. Prostate cancer with e-cadherin positivity, and Snail negativity and lobular breast cancer with negative e-cadherin and positive cytoplasmic Snail expression were used as controls (Supplementary Fig. S18). There was high e-cadherin expression in the normal lung epithelium of ME180 cell injected mice (Fig. 6C). Expression was reduced in GAN edited and control lentiviral tumor cells. Statistically significant enhanced e-cadherin expression was observed in GAN lentiviral tumor cells (*P* < 0.01, Fig. 6C and D). ME180 cells did not form metastasis. Thus, ME180 cell injected mice showed normal lung and Snail expression was seen in normal lung pneumocytes (Fig. 6E). GAN re-expression leads to 50% reduced metastasis in comparison with the control GAN edited and control lentiviral transfected cells. Thus, there was statistically reduced Snail expression in comparison with control lentiviral transfected cells (*P* < 0.01, Fig. 6E and F). The brown stains in the non-tumor regions of GAN re-expressing cells might reflect normal pneumocytes of the lung. Thus, both *in vitro* and mouse *in vivo* studies showed that gigaxonin expression has a direct relationship to e-cadherin and inverse relationship to Snail.

### Indirect Mechanism of Snail Downregulation by Gigaxonin

To determine the effect of gigaxonin on exogenously expressed Snail and to identify the mechanism of gigaxonin-Snail relationship, transfection studies were performed using 5 µg each of FLAG-tagged Snail and GAN plasmid DNAs. Two sets of gene transfected cells, one split 1:2 after 48-hour transfection and grown for an additional 24 hours, and the second set grown without splitting for 72 hours after transfection, were analyzed.

**FIGURE 7:**
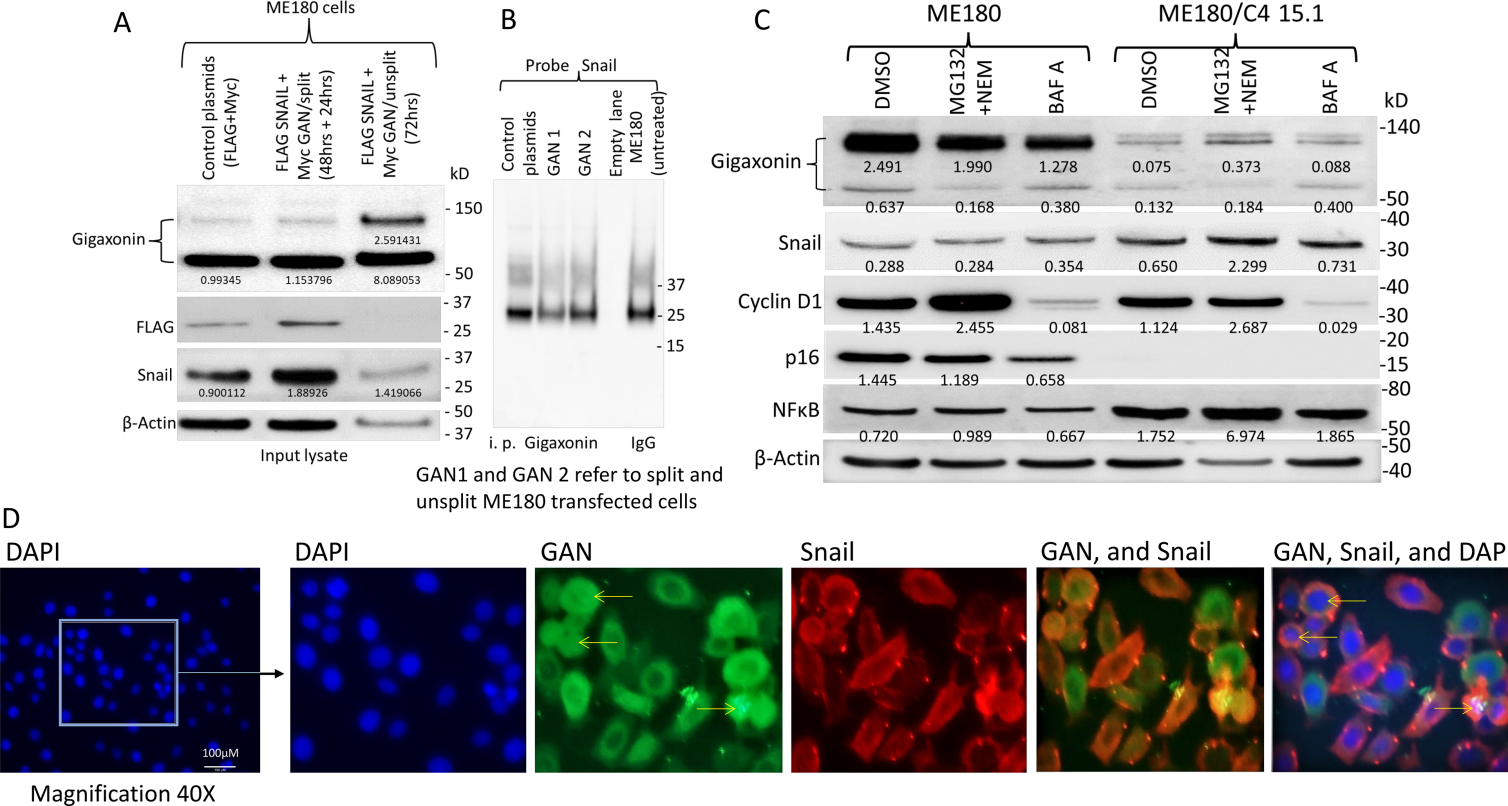
Gigaxonin-mediated regulation of Snail in gene transfected ME180 cells. **A,** FLAG-Snail and Myc-GAN plasmid DNA cotransfected ME180 cells show higher expression of FLAG and Snail in cells split 48 hours after transfection pointing to the expression of both exogenous (FLAG-Snail) and endogenous Snail in transfected cells. Expression of higher molecular weight GAN protein (140 kD) is reduced in these cells possibly containing higher fraction of fast growing cells. The unsplit cells show higher expression of gigaxonin (both lower and higher molecular weight proteins) due to the presence of slow growing cells in the population. These cells however have lost expression of FLAG-Snail and Snail confirming giagxonin mediated downregulation of exogenous and endogenous Snail. Reduced actin expression indicates that gigaxonin could be involved in actin ubiquitination. **B,** Immunoprecipitation with gigaxonin and hybridization to Snail shows hybridizing band to be same size as that of IgG immunoprecipitated proteins. Thus, we believe that there is no direct interaction between gigaxonin and Snail. **C,** Treatment of ME180 and GAN edited cells with inhibitors of proteosome (10 µmol/L each of MG132 and NEM) and autophagy (100 nmol/L of bafilomycin A) pathways shows increased expression of Snail in proteosome inhibitor–treated *GAN* edited cells in comparison to that of DMSO control treated cells pointing to the involvement of gigaxonin in Snail downregulation. As a confirmation of proteosomal inhibitor activity, there is increased expression of cyclin D1 (both ME180 and GAN edited cells) and NFκB of ME180/C4.15.1 cells in MG132/NEM-treated cell lines. Reduced expression of cyclin D1, p16, and gigaxonin after bafilomycin A treatment indicates blockage of cells, as expected at G_1_–S boundary, by bafilomycin A. The above results suggest that gigaxonin downregulates Snail through an indirect pathway, likely through NFκB. Numbers at the bottom of the protein bands indicate ratio of band intensity versus β-actin. Although there is reduced β-actin in proteosomal inhibitor lane (fifth lane), there is higher protein band intensity in both Snail and *NFκB* in comparison with DMSO-treated cells. This is also reflected in band intensity/β-actin numbers. **D,** Immunofluorescence analysis of ME180 cells shows endogenous expression of *GAN* and Snail in the cytoplasm, surrounding the nucleus. *GAN* nuclear expression was seen in 10% to 20% of cells (indicated by arrows). While overlap of the two proteins is seen in many cells, there are also cells expressing Snail or GAN alone pointing to the expression of both proteins in ME180 cells.

Both the control and plasmid transfected cells showed reduced expression of the higher molecular weight gigaxonin in 48-hour split cells (Fig. 7A). There was increased FLAG and Snail (combined endogenous and exogenous Snail) expression in gene transfected cells in comparison with liposome transfected cells. The unsplit cells revealed higher gigaxonin expression and loss of Snail expression (using both the FLAG and Snail antibodies) confirming Snail degradation with the expression of gigaxonin. There was reduced actin expression in the unsplit cells that could be due to gigaxonin mediated regulation of actin or the effect of combined Snail and gigaxonin expression.

Western blots of immunoprecipitated proteins with FLAG and Snail antibodies hybridized to a protein with similar size as that of the control IgG immunoprecipitated proteins (Fig. 7B). This indicated that it is not possible to demonstrate Snail interaction with immunoprecipitation studies. It is also possible that the hybridizing protein is not Snail, but IgG. In the reverse experiment where the FLAG or Snail immunoprecipitated proteins were hybridized to gigaxonin, we did not observe hybridization to the 60 or 140 kD gigaxonin protein confirming that there is no direct interaction between gigaxonin and Snail.

Because Snail expression is lost with the overexpression of gigaxonin (Fig. 7A; Supplementary Fig. S16A), we wanted to find out whether Snail degradation occurs through ubiquitination. We performed expression studies on cell lines treated with inhibitors of proteosomal (MG132 and n-ethylmaleimide) and autophagy (bafilomycin A) pathways. As has been shown earlier for the activity of MG132/NEM and bafilomycin A on cyclin D1 (36, 37), we found increased cyclin D1 expression in the presence of proteosomal inhibitors (MG132 and NEM) and decreased expression in the presence of bafilomycin A confirming that the inhibitors were functionally active (Fig. 7C). Proteosomal inhibitors did not affect the expression of gigaxonin and p16. We observed a 50% reduced expression of p16 and gigaxonin with bafilomycin A indicating a direct association between these two proteins and cell cycle blockage at G_1_–S boundary by bafilomycin A.

With the loss of gigaxonin in *GAN* edited cells, there was increased expression of Snail in the presence of proteosomal inhibitors, but there was no effect of bafilomycin A in comparison with the untreated cells (Fig. 7C). Absence of increased Snail expression with proteosomal inhibitors in the control ME180 cells could be attributed to low level expression of Snail and robust expression of gigaxonin even in the presence of proteosomal inhibitors. We also found increased NFκB expression in *GAN* edited cells correlating to decreased NFκB ubiquitination in the absence of p16 and gigaxonin. Absence of an effect by bafilomycin A suggesting that NFκB degradation does not involve autophagy.

Immunofluorescence analysis was done on ME180 cells to identify cellular expression of gigaxonin and Snail. There was overlapping expression of the two proteins in the cytoplasm, surrounding the nucleus (Fig. 7D). There were also cells expressing gigaxonin or Snail alone. Nuclear expression of gigaxonin was observed in 10% to 20% of cells (indicated by arrows).

To determine whether Snail ubiquitination occurs correlating to gigaxonin-mediated downregulation of Snail, proteosomal inhibitor–treated ME180 and *GAN* edited cells were immunoprecipitated with Snail and hybridized to ubiquitin antibody. Higher molecular weight proteins were not seen in ME180 cells pointing to the absence of Snail ubiquitination (Supplementary Fig. S19A). To probe Snail downregulation further, control and *GAN* siRNA transfected and proteosomal inhibitor–treated ME180 cells were hybridized to NFkB. There was increased NFκB expression with *GAN* siRNA in comparison with control siRNA (Supplementary Fig. S19B). Ubiquitin hybridization showed the presence of higher molecular weight bands in ME180 cells (Supplementary Fig. S19C). Lighter intensity bands could also been in control liposome and siRNA-treated cells. These bands were absent in *GAN* siRNA treated cells indicating gigaxonin is involved in NFκB ubiquitination and loss of gigaxonin leads to loss of NFκB ubiquitination.

### Loss of Exon 8 T Allele Expression is Associated with the Loss of Gigaxonin Multimeric Protein Formation and mRNA Instability

**FIGURE 8:**
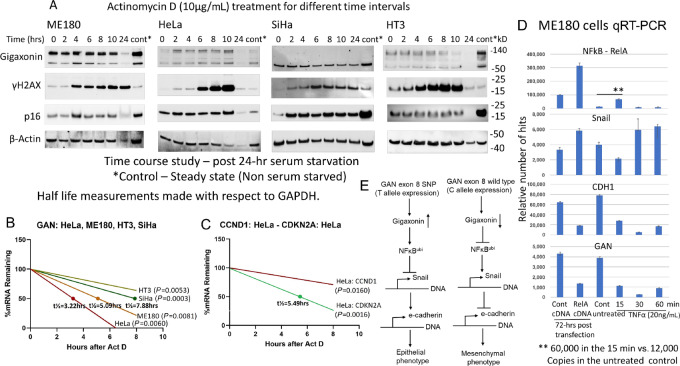
Absence of T allele expression is associated with absence of multimeric protein formation and mRNA instability. **A,** Western blot analysis of ME180, HeLa, HT3, and SiHa cells post actinomycin D treatment (10 µg/mL, 8 µmol/L) shows enhanced γH2AX expression in all four cell lines pointing to positive drug effect. Higher expression of 60 kD gigaxonin protein is seen in ME180, HT3, and SiHa cells in comparison with that of HeLa cells. Multimeric forms of the protein are absent in SiHa cells pointing to the association between loss of T allelic expression and absence of multimeric protein formation. **B,** RNA stability assays show half-life of HeLa cells to be 3.22 hours and that of ME180 and SiHa cell lines to be 5.09 and 7.88 hours, respectively. *GAN* RNA is more stable in HT3 cells. These results therefore indicate instability of *GAN* mRNA in HeLa cells showing C allele expression. **C,** Half-live of p16 (*CDKN2A*) is 5.49 hours and cyclin D1 mRNA is more stable in HeLa cells, matching the half-lives reported in the literature (34, 35). These results therefore indicate that actinomycin D treatment was effective in the cancer cell lines. **D,** qRT-PCR studies of ME180 cells transfected with RelA cDNA (3-fold) or treated with TNFα (5-fold after 15 minutes) show activation of NFκB compared with control cDNA or untreated control cells, respectively. There is activation of Snail in RelA transfected cells (2-fold) and in 30 and 60 minutes treatment of TNFα (1.6-fold) confirming transcription activation of Snail by *NFκB*. The time lag to NFκB may be related to time delay in the activation of NFκB signaling pathway. Downregulation of *CDH1* (e-cadherin) and *GAN* (gigaxonin) coinciding with Snail upregulation points to transcriptional downregulation of these two genes. While inhibition of e-cadherin transcription by Snail is known (58–60), we for the first time show transcriptional regulation of GAN with NFκB activation indicating a feedback regulation. **E,** A mechanism is proposed where *GAN* gene exon 8 SNP (T allele) expression is associated with enhanced gigaxonin expression (upward arrow) leading to ubiquitination of *NFκB* resulting in Snail downregulation, increased expression of e-cadherin and maintenance of epithelial phenotype. Loss of gigaxonin through C allele expression (downward arrow) leads to enhanced *NFκB* expression, activation of Snail, downregulation of e-cadherin and development of mesenchymal (EMT) phenotype. Our results extend the interrelationship between these genes reported earlier by the other investigators (52–55). → Activation, ┤ Inhibition.

To identify the molecular mechanism of association between exon 8 T allele and gigaxonin expression, RNA stability assays were performed. Activation of DNA damage response represented by γH2AX expression was used as a marker of transcription inhibitor actinomycin D treatment in ME180 cells. Enhanced γH2AX expression was observed with different concentrations of actinomycin D (Supplementary Fig. S20). There was cell death by 24 hours even for treatment with 0.1 µg/mL of the drug. Maximal γH2AX expression observed in 7 hours for the 10 µg/mL concentration was used in subsequent experiments. Higher expression of γH2AX expression was observed in drug-treated HeLa and HT3 cells in comparison to that of ME180 and SiHa cells (Fig. 8A). Multimeric forms of gigaxonin observed in ME180, HeLa, and HT3 cells was absent in SiHa cells. Because *GAN* gene mutations are not present in SiHa cells, we suggest that loss of multimeric protein formation, a posttranslational event, could also be associated with the loss of T allele expression.

RNA expression levels as measured by qRT-PCR showed half-life of *GAN* mRNA to be 3.22 hours in HeLa cells (*P* = 0.0060) while it was 5.09 hours for ME180 (*P* = 0.0081) and 7.88 hours for SiHa cells (*P* = 0.0003; Fig. 8B). The mRNA was stable in HT3 cells. While ME180 cells had increased mRNA instability than SiHa cells, there was an association between loss of exon 8 T allele expression and mRNA instability in HeLa cells in comparison with T allele expressing ME180 and HT3 cell lines. As a confirmation of actinomycin D effect, we found that the half-life of HeLa cell p16 to be 5.49 hours (*P* = 0.0016) and cyclin D1 RNA to be stable (*P* = 0.0160; Fig. 8C). The half-lives of the two proteins matched with half-lives (4.16 hours for p16 and stable expression for cyclin D1) reported in the literature (36, 37). Our results therefore showed that actinomycin D treatment was functional in the four cancer cell lines and that absence of T allele expression was associated with gigaxonin mRNA instability in HeLa cells.

To determine whether NFκB is responsible for the upregulation of Snail that in turn results in downregulation of e-cadherin, qRT-PCR analysis was performed on ME180 cells transfected with RelA cDNA or TNFα (20 µg/mL) up to 1 hour. There was activation of NFκB in RelA transfected cells in comparison with that of control cDNA transfected cells (Fig. 8D). There was enhanced NFκB expression 15 minutes after exposure to TNFα in comparison with untreated control cells. Snail was transcriptionally activated with RelA cDNA and at 30 and 60 minutes after exposure to TNFα. Time delay between NFκB and Snail activations could be attributed to a delay in NFκB-mediated signaling. Downregulation of e-cadherin coincided with Snail activation pointing to transcriptional downregulation of e-cadherin by Snail confirming the direct relationship between NFκB and Snail and inverse relationship between Snail and e-cadherin as was reported by others (52–57). We have also observed transcriptional downregulation of GAN after NFκB activation, as was seen with cisplatin treatment (see Fig. 2G) pointing to a feedback regulation between these two genes. From our results, we propose a mechanism where gigaxonin regulates NFκB through ubiquitination and NFκB mediated transcriptional activation of Snail results in transcriptional downregulation of e-cadherin (Fig. 8E).

## Discussion

GAN gene mutations have not been reported in head and neck cancer. Also, there is no information in databases for exon 8 SNP and head and neck cancer survival. However, the Stanford University cancer and survival database https://precog.stanford.edu shows expression of p16, gigaxonin, and e-cadherin (CDH1) to be associated with better overall survival and expression of Snail, Twist1, and n-cadherin (CDH2) to be associated with poor overall survival of head and neck cancer (Supplementary Fig. S21). We hypothesize that exon 8 T allele expression showing a direct relationship to gigaxonin expression and inverse relationship to EMT markers, plays a role in the inhibition of aggressive tumor cell growth, thereby improving survival of cancer subjects.

Snail is a transcription factor that inhibits e-cadherin expression accompanied by progression to the EMT phenotype. Snail is reported to be overexpressed in aggressive head and neck and other human cancers (52, 57). It has also been shown that the loss of Snail through inhibitors leads to the reversal of EMT phenotype. Conversely, overexpression of Snail is shown to convert less aggressive head and neck cancer to aggressive cancer, strongly correlating Snail expression to tumor cell metastasis (57–60). While stabilization of Snail through BDR4 or TGFβ-activated USP27X deubiquitinase has been shown, destabilization has not yet been demonstrated (57). In the absence of *GAN* expression in head and neck cancer cell lines, we used ME180, a cervical cancer cell line with higher levels of *GAN* and basal Snail expression to study the relationship between these two proteins. We show that Snail deregulation occurs with gigaxonin expression resulting in the inhibition of EMT. Our studies indicate that this inhibition is due to gigaxoin-mediated ubiquitination of NFκB.

Although we isolated T>C converted faster growing *GAN* edited cells from slow growing ME180 cells, the reverse isolation of C>T of HeLa cells was not successful. This could be due to fast growing parental HeLa cells masking the isolation of slow growing *GAN* edited cells. It is to be noted that HeLa and C33A cells contain both C and T alleles and yet express only the C allele, indicating loss of slow growing T allele containing cells during the isolation of these cell lines.

Our previous investigation (30) and current results (see Fig. 7C; Supplementary Fig. S19B and S19C) have shown downregulation of NFκB through ubiquitination. Protein interactome studies have shown that NFκB interacts with several EMT signaling proteins including Snail, Twist, and CBP/p300: Protein–Protein Interactions » NFκB Transcription: https://www.bu.edu/nf-kb/physiological-mediators/interacting-proteins. These studies have also shown that there is an interaction between *NFκB* and actin, possibly explaining reduced β-actin expression in some gigaxonin-overexpressing cells. Because, equal concentration of proteins was loaded in Western blots, loading is not the reason for the actin downregulation shown in Fig. 7A. Tubulins have also been shown to be downregulated by gigaxonin making it difficult to use other loading controls (61). It has also been shown by Barberà and colleagues (52) that NFκB regulates Snail expression through transcriptional control. We have data pointing to transcriptional upregulation of Snail by *NFκB* which in turn leads to transcriptional downregulation of e-cadherin. We therefore suggest that gigaxonin downregulates Snail expression through downregulation of NFκB (Fig. 8E).

We have observed expression of Twist1 in *GAN* edited cells in the absence of p16 and gigaxonin (see Fig. 3). We have observed cervical cancer cell lines to have basal expression of Snail and Twist1, and head and neck cancer cell lines exhibiting higher Twist1 expression indicating a distinct biology governing cervical and head and neck cancers. In support of our data pointing to the relationship between p16, *GAN*, and *Twist1*, TCGA (http://ualcan.path.uab.edu/cgi-bin/TCGA-survival1.pl?genenam=TWIST11&ctype=HNSC) and (http://www.progtools.net/gene/index.php database; this later database is cited in https://bmccancer.biomedcentral.com/articles/10.1186/1471–2407–14–970) shows that, overexpression of CDKN2A (p16; *P* = 0.0756202), and GAN (*P* = 0.0108553) are associated with better overall survival of patients with metastatic head and neck cancers (Supplementary Fig. S22). The combined effect of CDKN2A and GAN expression for better overall survival is statistically significant (*P* = 0.023389), although GAN alone (*P* = 0.0108553) has a better statistical significance. Furthermore, recurrence of primary laryngeal cancers is directly associated with over expression of Twist1 (*P* = 0.0093843). We suggest therefore that, in addition to Snail, expression of Twist1 is also a prognostic marker of metastatic head and neck cancer.

Gigaxonin has many partners, probably >100 (8). For now, all known partners are shown to be regulated directly: Gigaxonin interacts with the rod domain of all IF proteins (explaining why all IF are aggregating in patients and animal models), and with the WD40 domain of ATG16L1. These findings have been shown by direct (immunoprecipitation), and indirect (proximity ligation assay staining) binding studies and reveal a direct interaction between gigaxonin and its partner proteins. We show in the present investigation that downregulation of Snail by gigaxonin is indirect. We demonstrate that downregulation of NFκB leads to downregulation of Snail which in turn leads to increased expression of e-cadherin and maintenance of epithelial phenotype. Loss of gigaxonin leads to activation of NFκB and Snail and inactivation of e-cadherin resulting in the development of mesenchymal phenotype. In addition, our results suggest a feedback regulation between gigaxonin and NFκB that would require extensive future studies to confirm and extend the findings. Our data also provide opportunities for preclinical therapeutic studies for GAN activation using NFkB inhibitors.

There are multiple neurogenerative diseases that are implicated in human cancer (Supplementary Table S7). Depending on the type of protein that is accumulated in the disease, the relationship to cancer is altered. In the gradual neurodegenerative diseases Alzheimer's and Parkinson's, there is a lower cancer incidence. However, more aggressive neurodegenerative diseases such as Ataxia-telangiectasia seems to have increased cancer incidence through gene mutations, leading to inhibition of DNA damage repair pathway (Supplementary Table S7). In conclusion, we show for the first time that GAN gene product gigaxonin involved in the control of neuronal maintenance is also a human metastasis suppressor protein. We conclude that (i) GAN gene exon 8 SNP T allele expression is associated with RNA stability, (ii) loss of T allele results in the loss of gigaxonin expression, (iii) activation of NFκB, (iv) upregulation of Snail, (v) downregulation of e-cadherin, and (vi) development of chemoradiation resistance and faster tumor cell growth.

## Supplementary Material

Supplementary Figure Legends Supplementary figure legends

Supplementary Methods Supplementary methods

Supplementary Table 1 GAN gene exon primers

Supplementary Table 2 Exon 8 SNP frequency in normal vs tumors

Supplementary Table 3 Exon 8 SNP in primary vs recurrence/metastasis

Supplementary Table 4 Exon 8 SNP in cancer cell lines

Supplementary Table 5 GAN gene RT-PCR primers

Supplementary Table 6 CRISPR-Cas9 oligonucleotides

Supplementary Table 7 Neurodegenerative diseases and cancer

Supplementary Table 8 Antibody details

Supplementary Figure 1 Mitochondrial gene authentication primers

Supplementary Figure 2 Morphology of fibroblast and cancer cell lines

Supplementary Figure 3 SNP detection and CDKN2A and GAN expression in cancer cell lines

Supplementary Figure 4 MTT and sift agar assay and Snapshot view of exon 8 of C33A and HT3 cell lines

Supplementary Figure 5 Representative soft agar colonies of ME180, HeLa, and Siha cell lines

Supplementary Figure 6 Incucyte live cell assay of cisplatin treated ME180 cells

Supplementary Figure 7 ME180 and CRISPR-Cas9 clones

Supplementary Figure 8 Map of lentiviral cloning vector

Supplementary Figure 9 Synonymous exome duplication sequences in ME180 and GAN edited cell lines

Supplementary Figure 10 Synonymous exome SNP and Indel sequences in ME180 and GAN edited cell lines

Supplementary Figure 11 All non synonymous SNPs in ME180 and GAN edited cell lines

Supplementary Figure 12 expression of GAN, senescence and autophagy genes

Supplementary Figure 13 RNA differential expression in mE180 and GAN edited cell lines

Supplementary Figure 14 Snapshot view of exon 8 in ME180 and GAN edited cell lines

Supplementary Figure 15 Invasion and migration of control and GAN siRNA treated ME180 cells

Supplementary Figure 16 Control, GAN siRNA and GAN overexpression effect on transcription factors in ME180 and HeLa cell lines

Supplementary Figure 17 Subcutaneous xenograft tumor formation in nude (NSG) mice

Supplementary Figure 18 E-cadherin and Snail expression in breast and prostate cancer samples

Supplementary Figure 19 Ubiquitination assay for GAN binding to Snail and NF-kB

Supplementary Figure 20 expression of GAN, gamma H2AX, and p16 post Act D treatment of ME180 cells

Supplementary Figure 21 Survival probability of head and neck cancer

Supplementary Figure 22 Overall survival of head and neck cancer
