# Supplementary material for: Gigaxonin Suppresses Epithelial-to-Mesenchymal Transition of Human Cancer Through Downregulation of Snail
Source: Cancer Res Commun. 2024 Mar 8;4(3):706–22. doi: 10.1158/2767-9764.CRC-23-0331 (PMC10921914; doi:10.1158/2767-9764.CRC-23-0331)
Supplement: Supplementary Figure Legends [file crc-23-0331-s01.docx]

Supplementary figure legends

Supplementary figure 1. Cell authentication using mitochondrial DNA repeat sequences HVR1 and HVR2. Primer sequences used for PCR are shown in green and the PCR product size for HVR1 And HVR2 are 535bp and 499bp respectively. Sequences were taken from the ref. Andrew et al Nat Genet 1999, 23:147.

Supplementary figure 2. Cell authentication using morphology. Microscopic images (10X and 20X) of fibroblast cell line GM05399, and head and neck and cervical cancer cell lines show different morphologies, indicating differences in cellular phenotypes used in the investigation.

Supplementary figure 3. Stability of GAN RNA in HT3 and ME 180 cells. A) PCR products were digested with TspGW1 restriction enzyme and separated on 10% PAGE gels. Product size of 155/132bp (wild type C allele), 287bp (SNP, mutant T allele) and 287 and 155/132bp (hybrid C/T alleles) are visualized. Sequencing of PCR products confirm the presence of wild type vs SNP and hybrid alleles. B-C) Fold differentail expression with respect to a fibroblast cell line GM05399 (ΔΔCt -differential cell cycle expression of GAPDH vs gene of interest compared to GAPDH vs gene expression in GM05399) shows CDKN2A expression higher at 6hrs and reduced at 24hrs in all 4 cell lines. GAN expression however, is stable in ME180 and HT3 than HeLa and C33A cells. Head and neck cancer cell lines UM-SCC1 and UM-SCC14A do not show expression of these two genes.

Supplementary figure 4. A-C) HT3 cells containing T/T alleles show statistically significant slower *in vitro* growth, soft agar colony formation and increased sensitivity to cisplatin in comparison to C/T allele containing C33A cells. D) Snapshot view (IGV) of exon 8 shows GAN expression to be from T allele (red) in HT3 and C allele (blue) in C33A cells.

Supplementary figure 5. Reduced soft agar colony formation in ME180 cells. A) A representative soft agar colony figure shows smaller colonies in ME180 cells.in comparison to HeLa cells. B-C) Hela cell show reduced soft agar colony formation and increased cisplatin sensitivity in comparison to SiHa cells.

Supplementary figure 6. Cell images of the Incucyte assay. Live ME180 cells are visualized at 72-hrs post in 1.0 and 3.0μg/ml cisplatin treated cells.

Supplementary figure 7. CRISPR-Cas9 clones of gRNA1 showing similar morphology to parental cell line ME180. Two different clones ME180/1 C1.1 and ME180/1 C1.2 of gRNA1 transfection show similar morphology to ME180 cells. These cell lines were not pursued further.

Supplementary figure 8. Map of *GAN* gene lentiviral vector indicating MCS for cloning the *GAN* gene. This plasmid vector is available from Thermo Fisher Scientific.

Supplementary figure 9. Sequence similarity of CRSPR-Cas9 clones to ME180 cells. Circos plots of the exome sequence analysis shows near identical of synonymous genomic duplications in CRISPR-Cas9 clones to that of parental ME180 cells, indicating that the clones are true derivatives of ME180 cells.

Supplementary figure 10. Genomic sequencing shows overlap of SNPs and indels in the ME180, GAN edited and lentiviral transfected cell lines.

Supplementary figure 11. Non-synonymous sequence identity in CRISPR-Cas9 clones to the parental ME180 cells. A-C) Circos plots of non-synonymous (mutations) sequences show sequence similarity in the CRISPR-Cas9 and ME 180 cell lines. D) Venn diagram shows an overlap of two-thirds of non-synonymous sequences and one-third of sequences that are unique to ME180 indicating loss of these sequences during derivation of gRNA 4 single cell CRISPR-Cas9 clones.

Supplementary figure 12. Partial reversal of CRISPR-Cas9 morphology with lentiviral *GAN* gene. Re-expression of gigaxonin through lentiviral GAN gene transfection (ME180/C4.15.1/LV GAN 50) leads to an intermediate morphology of ME180 and CRISPR-Cas9 clone C4.15.1.

Supplementary figure 139. Changes in the expression of cytoskeletal genes and cathepsin C with gigaxonin overexpression. RNA seq data shows re-expression in lentiviral mediated transfer of the GAN gene results in partial re-expression of cytoskeletal proteins and cathepsin C.

Supplementary figure 14. A snap-shot view of exon 8 of RNA seq shows T allele expression of gigaxonin in ME180 and high expression in *GAN* gene transfected cells. There is minimal expression of exon 8 in CRISPR-Cas9 and control viral transfected cells. Horizontal red lines in these two cell lines points to exon 8 sequence deletions.

Supplementary figure 15. Increased migration and Matrigel invasion associated with the loss of gigaoxinin expression. A) ME180 cells treated with GAN siRNA shows loss of gigaxonin expression in comparison to control, liposome, and control siRNA transfected cells. B-E) Statistically significant increased migration (p<0.0014) and Matrigel invasion (p<0.0008) are observed in GAN siRNA transfected cells.

Supplementary figure 16. Inverse relationship between GAN and NF-κB. A) While decreased gigaxonin expression with GAN siRNA shows increased NF-κB and snail expression, decreased expression of the two proteins is visualized with overexpression of gigaxoin with GAN cDNA, B) There is reduction in Zeb1 expression in Hela cells with overexpression of gigaxonin.

Supplementary figure 17. Mouse subcutaneous xenograft tumor cell growth is observed in 6 of 6 ME180 injected animals. There is tumor growth, although reduced, in 4 of 6 mice of GAN lentiviral transfected cells. CRISPR-Cas9 cells produced small tumor growth in 1 of 6 animals. There was no tumor growth in the remaining 5 animals and in 6 of 6 of control viral transfected cells.

Supplementary figure 18. Lobular cancer of the breast shows e-cadherin expression in ductal epithelial cells and cytoplasmic expression of snail in the lobular cells. Prostate cancer cells show e-cadherin expression in the epithelial cells and weak cytoplasmic snail expression in the tumor cells. These two tumor samples served as controls for e-cadherin and snail immunohistochemical hybridizations respectively.

Supplementary figure 19. Absence of Snail ubiquitination and NF-κB ubiquitination with gigaxonin expressin. A) Immunoprecipitation of ME180 and GAN edited cells with Snail antibody post proteosomal inhibitor treatment and hybridization to ubiquitin antibody does not show higher molecular weight bands indicating absence of Snail ubiquitination. B) GAN siRNA treated ME180 cells in the presence of proteosomal inhibitors shows decreased gigaxonin and increased NF-κB expression. C) Ubiquitination assay shows higher molecular weight bands poiting to ubiquitination of NF-κB by gigaxonin and loss of ubiquitination in GAN siRNA expressing cells.

Supplementary figure 20. Increased γH2AX expression post actinomycin D treatment of ME180 cells. Cells (1X10^6^) were plated onto 100mm tissue culture dishes in RMPI plus 10%FBS medium. After 2 days of growth (60 to 70% confluency), cells were treated for 24hrs in serum free media and in serum plus media for 12hrs prior to actinomycin D treatment. Stock actinomycin D solution (Cell Signaling Technology, Danvers, MA, 12mg/ml, 10mM) made in DMSO was used for treatment with different concentrations. There was increased γH2AX expression from 3hr post treatment of 1μM and effect was sustained at 7hrs at higher concentration. There was reduced expression of p16 at 24hrs reflecting cell death.

Supplementary figure 21. Direct relationship between gigaxonin and head and neck cancer survival. Kaplan-Meier survival plots of Stanford University database <https://precog.stanford.edu> of head and neck cancer shows higher expression of gigaxonin, p16 and e-cadherin correlating with better overall survival, and those with higher expression of Snail, Twist1 and n-cadherin correlating with poor overall survival.

Supplementary figure 22. TCGA database (<http://ualcan.path.uab.edu/cgi-bin/TCGA-survival1.pl?genenam=TWIST1&ctype=HNSC>) shows a direct relationship of overall survival post lymph node metastasis to A) CDKN2A (p16) and B) GAN expression and inverse relationship to C) Twist1 expression. D) Twist1 expression is also directly associated with the recurrence of laryngeal primary tumors.
