# Supplementary material for: Gigaxonin Suppresses Epithelial-to-Mesenchymal Transition of Human Cancer Through Downregulation of Snail
Source: Cancer Res Commun. 2024 Mar 8;4(3):706–22. doi: 10.1158/2767-9764.CRC-23-0331 (PMC10921914; doi:10.1158/2767-9764.CRC-23-0331)
Supplement: Supplementary Methods [file crc-23-0331-s02.docx]

**Supplementary data**

**MTT [3-(4,5-dimethylthiozol-2-yl)-2,5-diphenyl tetrazolium bromide] assay for cell viability.** Control and cisplatin treated cells were grown in 24 well tissue culture dishes for 24 to 144 (6 days) hours. The MTT assay was carried out following a previously established protocol^30^. The assays were performed in quadruplicates and repeated thrice.

**PCR, and RT-qPCR studies.** These studies were performed according to established methods^34-37^. Primary tumor RNAs from FFPE slices were isolated using the Qiagen RNase easy FFPE kit (catalog number 73504, Qiagen, Germantown, Maryland). RNAs from 24-hr serum starved or steady state (non-serum starved) cell lines were prepared using the Qiagen mini RNase easy kit (Catalogue number 74104). Quantitative RT-PCR (RT-qPCR) was performed in quadruplicates using Applied Biosystems RT-qPCR equipment with the company provided kit and gene specific primers (Supplementary Table 3). RT-qPCR for NF-κB (RelA) and Snail were performed using the qPCR primer pairs (Catalogue numbers HP214292 for RelA, and HP209016 for Snail primer pairs) and SYBR Master Mix Hi-ROX (Catalogue number QP100001) obtained from Origene Inc., Rockville, MD 20850. SuperScriptTM III Platinum One-Step qRT-PCR kit (Catalogue number 11732020) obtained from ThermoFisher Scientific, Carlsbad, CA, was used for RT-qPCR reactions. TNFα animal free recombinant protein (catalogue number AF-300-01A-10ug) was obtained from ThermoFisher Scientific for the NF-κB activation studies. RNA copy numbers were calculated with respect to GAPDH (ΔCt values) and the copy number of GAPDH seen from the RNA sequencing of HeLa cells (164,185 transcripts) as a reference. The experiments were performed thrice.

**CRISPR-Cas9 oligo preparation and cloning into px459 plasmid.** Four different gRNA CRISPR-Cas9 forward and reverse primers of 25 nucleotides (fig. 3A, supplementary Table 4) were created using the database <http://www.genome-engineering.org/crispr/?page_id=41>. The oligonucleotides were synthesized through IDT (integrated DNA Technologies, San Diego, CA). Stock solutions of 200μm were made for each of the primers. The forward and reverse gRNA primers were annealed in a 20μl reaction in the annealing buffer (10mM Tris-HCl, pH 8.0, 50mM NaCl, and 1mM EDTA) to a final concentration 50μM at 95°C for 4 minutes and cooled slowly for 15 minutes at room temperature. A stock solution of 10μM annealed primers were used for ligating to the Bbs I site of CRISPR-Cas9 plasmid px459. The ligation in a 25μl reaction consisted of 2.5μl of 10X NEB restriction buffer 1, 1μl (1μg) of px459 plasmid, 1U of Bbs I enzyme, 2.5μl of 10X T4 ligase buffer, 1ul annealed primers (to a final concentration of 0.4μM using the 10μm stock solution), 1.5 units (1u/ul) of T4 DNA ligase. The reaction was carried out at 37°C for 1 hour and 1 to 5μl ligated products were used for transforming One Shot™ TOP10 Chemically Competent *E. coli* (Invitrogen, Carlsbad, CA) cells. Ampicillin resistant colonies were selected, and plasmid preparations were made using the Quigen midi prep kits. Insertion of gRNAs were confirmed by sequencing using the U6 forward and px459 reverse sequences (supplementary Table 4).

**CRISPR-Cas9 converted T>C allele containing single cell cloning**. The plasmids containing gRNA inserts were used for transfection into ME180 cells using the OptiMEM (Invitrogen Technologies, Carlsbad, CA) medium using the Invitrogen technologies transfectamine method. Puromycin in steps of 0.5, 1.0, 1.5 and 2μg/ml selection was applied 48 hours post transfection and over ten days (2 days per concentration selection). Then, the cells were grown in non-puromycin medium for 5 days and then back in puromycin (2μg/ml) medium for the selection of stable transfected colonies. DNA isolated from bulk colonies were used for GAN gene exon 8 PCR. Products were digested with TspGW1 restriction enzyme and separated on 10% PAGE-acrylamide gels to identify the presence of wild type 155/132bp C allele. Transformed bulk cells containing the C allele were used for single cell cloning using the 24 well/96 well/24 well/cloning ring method. DNAs of isolated CRISPR-Cas9 colonies were tested for the presence of exon 8 wild type C/C alleles using the exon 8 PCR/TspGW1 method and then confirmed by DNA sequencing.

**Small interfering RNA** (siRNA) studies were carried out with Dharmacon On-Target plus human GAN siRNA using the established protocol^30^. Studies were done at least thrice for each experiment.

**Western blotting analysis.** Western blotting was carried out following the standard published protocols^30,38,39^. The antibodies used for the blotting studies, their dilutions and catalog numbers are listed in supplementary Table 8. Briefly, cells were grown in RPMI medium to 70% confluence and the cell lysates were prepared with the lysis buffer containing protease and phosphatase inhibitors (50 mM HEPES, 200 mM KCl, 1 mM EGTA, 1mMMgCl2, 0.5mMdithiothreitol [DTT], 0.5% NP-40). The lysates were centrifuged at 15,000 rpm for 10 min, supernatants were collected, and proteins were quantified by Bradford assay (Biorad, Hercules, CA). Proteins (40ug) were electrophoresed on 4-12% gradient SDS-PAGE gels (Invitrogen, Carlsbad, CA), transferred onto PVDF membranes and hybridized to antibodies as described^30,33,38^. Membranes were developed using Supersignal West Pico Plus chemiluminescence reagents (Thermofisher Scientific, Carlsbad, CA) and images were captured using Biorad Chemidoc Imaging System (Biorad, Hercules, CA). Studies were repeated thrice for each experiment.

**Immunoprecipitation:** Immunoprecipitations were carried out using1mg of protein. Protein lysates were mixed with appropriate dilution of the antibodies or control IgG (as per vendor recommendations) and incubated overnight at 4^0^ C with gentle rocking. The following day, prewashed protein A/G beads (50ul) (Santacruz Biotechnology, Santacruz, CA) were added, and the mixtures were incubated for 2 h at 4°C. Samples were then centrifuged briefly (30 seconds) at 2000 rpm at 4^0^ C and the supernatant were collected. Pelleted beads were subsequently washed briefly for three times with 1X PBS. Further, proteins were eluted from the agarose beads with 1X sample buffer. Eluted samples were heated at 90^0^ C for 5 min and were run on 4-12% gradient gels. Further procedure was similar to the above mentioned Western blotting protocol. After secondary antibody hybridization, protein bands in the membranes were visualized using a BioRad Chemidoc imaging system. These studies were repeated thrice.

**Immunohistochemistry**. A standard protocol was followed for the immunohistochemical analysis of mouse lung tissues^39^. Paraffin-embedded sections were cut at 4μm thickness and paraffin removed with xylene and rehydrated through graded ethanol. Endogenous peroxidase activity was blocked with 3% hydrogen peroxide in methanol for 10 min. Heat-induced antigen retrieval (HIER) was carried out for all sections in AR9 buffer (AR9001KT, Akoya) using a Biocare decloaker at 95^o^C for 25 min. The slides were then stained with e-cadherin, 1:200, Rabbit mAb, Cat # 3195 (24E10) or Snail, 1: 100, Mouse mAb, Cat # 3895 (L70G2) (Cell Signaling technology, Danvers, MA) at 4°C overnight. Hybridization signals were detected using the BOND Polymer Refine Detection (Leica Biosystems, DS9800). Prostate cancer and invasive lobular carcinoma of the breast were used as positive controls for e-cadherin and snail antibodies respectively. Slides were stained with Hematoxylin and Eosin for pathological evaluation of normal, tumor, and metastasis phenotypes. Percent reactivity of protein expression was scored from 0-100 and the intensity of expression was scored as 0, 1, 2 and 3 with 3 being the maximum expression.

**Immunofluorescence.** ME 180 cells were grown to 60 to 70% confluency in cuvette (5000 cells per well of the 8 well cuvette), washed twice with cold PBS, and fixed with 4.0% paraformaldehyde for 4hrs at 4^0^ C. The fixed cells were washed twice with cold 1X PBS and cells were then permeabilized with 0.2% Triton X-100 for 10 minutes followed by blocking with 1% bovine serum albumin (BSA) in PBS for ~~15 minutes~~ one hour. Next, cells were incubated with primary antibodies (Snail (Novus Biotechne, CO) and Gigaxonin (Santa Cruz Biotechnology, CA) for an overnight at 4^0^C followed by PBS washing. Further, secondary antibodies tagged with Alexa-568 (anti-rabbit) and Alexa-488 (anti-mouse) were added and cells were incubated for another 30 minutes at room temperature, followed by washings with PBS.  They were then coverslipped after adding Vectashield (Vector Laboratories, Burlingame, CA) and observed using a Zeiss Axio microscope.
