# Supplementary material for: Gigaxonin Suppresses Epithelial-to-Mesenchymal Transition of Human Cancer Through Downregulation of Snail
Source: Cancer Res Commun. 2024 Mar 8;4(3):706–22. doi: 10.1158/2767-9764.CRC-23-0331 (PMC10921914; doi:10.1158/2767-9764.CRC-23-0331)
Supplement: Supplementary Table 1 — GAN gene exon primers [file crc-23-0331-s03.docx]

Supplementary Table 1. GAN gene exon PCR primers

| Exon | Location of exon in the RNA seq (from ATG) | Primers (localized to introns) | HS chr16 GRch38.p13  Genomic seq location | Product  size | Annealing  Temperature |
| --- | --- | --- | --- | --- | --- |
| 1 | -230~167 | 5’-GGAGCGACGGTGCGG-3’  5’-GCCGGGGACAGGG-3’ | 81314885-81314900  81315384-81315371 | 499 | *55* |
| 2 | 168-282 | 5'-ATA/GCT/ATT/TCT/GTT/CTT/TCA/TA-3'  5'-TATAATGGATGAAAGGAGACC-3' | 81351531-81351551  81351757-81351736 | 226 | *55* |
| 3 | 283-633 | 5'-GTTTGGGTTTTAAATGTACA-3'  5'-CAACTAAAATTTGAATTAAAAAGAAA-3' | 81354350-81354369  81354820-81354795 | 470 | *55* |
| 4 | 634-851 | 5'-CCCTCTTCTGCAGGTCCAC-3'  5'-TGGAACTACCTCTCCCATACAC-3' | 81356772-81356790  81357057-81357035 | 285 | *60* |
| 5 | 852-973 | 5'-TAAACTAAAACTAGTGTGGCTACT-3'  5'-GTATCTTTAAAAGGCTCTGAGTC-3' | 81357729-81357753  81358024-81358002 | 295 | *60* |
| 6 | 974-1086 | 5'-TCTTCAGATGCTGTTTCTATATATG-3'  5'-GCTCCGTTTCTTCCCTGAAC-3' | 81362411-81362435  81362702-81362686 | 291 | *60* |
| 7 | 1087-1236 | 5'-CAGCTTTCAATATGAT-3'  5'-CACCATCAGTTATATTAAAGGTTT-3' | 81363737-81363752  81364043-81364020 | 306 | *60* |
| 8 | 1237-1373 | 5'-ACAGTTTAATATCTGTTCACCT-3'  5'-AAAAGCCAGGCAGGGTAA-3' | 81364901-81364922  81365188-81365171 | 287 | *55* |
| 9 | 1374-1502 | 5'-TGCTGCAGAGTTAAACCAG-3'  5'-CAAAACTAAACAAAGCTAAAATA-3' | 81365258-81365276  81365557-81365535 | 299 | *55* |
| 10 | 1503-1612 | 5'-GATGACTCACCAAGCTTGCT-3'  5'-TCGTAATTGGTACCTAAGCC-3' | 81377128-81377149  81377427-81377408 | 299 | *55* |
| 11 | 1613-1794 | 5'-CTGTTTCCTGGTGATTCTGG-3'  5'-CTTTCGGAGCTATGTTATGG-3' | 81377358-81377377  81377657-81377638 | 299 | 55 |

HS- [*Homo sapiens* (human*)*]
