# Supplementary material for: Gigaxonin Suppresses Epithelial-to-Mesenchymal Transition of Human Cancer Through Downregulation of Snail
Source: Cancer Res Commun. 2024 Mar 8;4(3):706–22. doi: 10.1158/2767-9764.CRC-23-0331 (PMC10921914; doi:10.1158/2767-9764.CRC-23-0331)
Supplement: Supplementary Table 2 — Exon 8 SNP frequency in normal vs tumors [file crc-23-0331-s04.docx]

Supplementary Table 2. *GAN* gene exon 8 SNP frequency in normal vs tumors

| Samples | Number | HPV positive 16, 18, others (total) | HPV Negative | Wild type C/C allele | Heterozygous C/T alleles | Homozygous T/T allele | Percentage of C/T + T/T |
| --- | --- | --- | --- | --- | --- | --- | --- |
| Normal samples | 53 | 0/53 | 53 | 40 | 12 | 1 | 24.5 |
| Cervical Cancer* I City of Hope | 26 | 13, 2, 5 (20) | 6 | 13 | 10 | 3 | 50.0 |
| Cervical Cancer II City of Hope | 26 | 16, 4, 3 (23) | 3 | 14 | 10 | 2 | 46.2 |
| HNSCC I City of Hope | 28 | 13, 0, 4 (17) | 11 | 16 | 7 | 5 | 42.9 |
| HNSCC II UCLA | 20 | 10, 0, 0 (10) | 10 | 10 | 5 | 5 | 50.0 |

VAGLAHS 22 1, 0, 0 (1) 21 13 6 3 40.9

Tumor 56/122 = 45.9% vs Normal 13/53 = 24.5%, **p=0.011^a^** Significant

**^a^Fisher’s exact test, two-sided**

*19 normal DNAs were from adjacent endometrium of tumor samples. Heterozygous alleles were observed in both the normal and tumor DNAs of 7 samples. The other 12 samples had C or T allele in both the normal and tumor samples. Remaining 34 normal DNAs were random samples. They were from normal squamous epithelial cells.
