# Supplementary material for: Gigaxonin Suppresses Epithelial-to-Mesenchymal Transition of Human Cancer Through Downregulation of Snail
Source: Cancer Res Commun. 2024 Mar 8;4(3):706–22. doi: 10.1158/2767-9764.CRC-23-0331 (PMC10921914; doi:10.1158/2767-9764.CRC-23-0331)
Supplement: Supplementary Table 3 — Exon 8 SNP in primary vs recurrence/metastasis [file crc-23-0331-s05.docx]

Supplementary Table 3. *GAN* gene exon 8 SNP in primary vs recurrence and metastasis

Exon 8 SNP Primary tumor Metastasis/Recurrence p value of Primary vs metastasis/recurrence

C/T + T/T 43/86 = 50% 13/36 = 36.1% **p=0.171^a^**

C/T 29/86 = 33.7% 10/36 = 27.8% **p=0.671^a^** Not significant

T/T 14/86 = 16.3% 3/36 = 8.3% **p=0.390^a^**

**^a^Fisher’s exact test, two-sided**
