# Supplementary material for: Gigaxonin Suppresses Epithelial-to-Mesenchymal Transition of Human Cancer Through Downregulation of Snail
Source: Cancer Res Commun. 2024 Mar 8;4(3):706–22. doi: 10.1158/2767-9764.CRC-23-0331 (PMC10921914; doi:10.1158/2767-9764.CRC-23-0331)
Supplement: Supplementary Table 4 — Exon 8 SNP in cancer cell lines [file crc-23-0331-s06.docx]

|  | Cell Line | Cancer type | HPV status | Exon 8 RFLP (bp) | Ex 1-11 DNA seq^a^ | ex 1-11 mRNA seq^b^ | GAN c.1293 C/T^c^ |  |  |  |  |  |  |
| --- | --- | --- | --- | --- | --- | --- | --- | --- | --- | --- | --- | --- | --- |
|  | CAL-27 | Head-Neck | negative | 155/132 | Done | Done | 100/0 |  |  |  |  |  |  |
|  | SCC-1 | Head-Neck | negative | 155/132 | Done | Done | 100/0 |  |  |  |  |  |  |
|  | SCC-12 | Head-Neck | negative | 155/132 | Not done | Done | 100/0 |  |  |  |  |  |  |
|  | SCC-14A | Head-Neck | negative | 155/132 | Not done | Done | 100/0 |  |  |  |  |  |  |
|  | CCL-23 | Head-Neck | 18 | 287/155/132 | Done | Done | 30/70 |  |  |  |  |  |  |
|  | C-33 A | Cervix | negative | 287/155/132 | Not done | Done | 50/50 |  |  |  |  |  |  |
|  | HT-3 | Cervix | negative | 287 | Not done | Done | 0/100 |  |  |  |  |  |  |
|  | MS751 | Cervix | 16 | 155/132 | Not done | Not done | 100/0 |  |  |  |  |  |  |
|  | SiHa | Cervix | 16 | 287/155/132 | Done | Done | 20/80 |  |  |  |  |  |  |
|  | HeLa | Cervix | 18 | 287/155/132 | Done | Done | 60/40 |  |  |  |  |  |  |
|  | ME-180 | Cervix | 18 | 287 | Done | Done | 0/100 |  |  |  |  |  |  |
|  | C-4 I | Cervix | 18 | 155/132 | Not done | No | 100/0 |  |  |  |  |  |  |
|  | LAN-6 | Neuroblastoma | negative | 155/132 | Not done | Not done | 100/0 |  |  |  |  |  |  |

Supplementary Table 4. Exon 8 SNP in cancer cell lines

a – sequence of individual GAN gene exons 1 to 11, b – sequence of the RT-PCR products,

c – Percentage of C and T alleles calculated from exon 8 acrylamide gel band intensities of DNA samples
