# Supplementary material for: Gigaxonin Suppresses Epithelial-to-Mesenchymal Transition of Human Cancer Through Downregulation of Snail
Source: Cancer Res Commun. 2024 Mar 8;4(3):706–22. doi: 10.1158/2767-9764.CRC-23-0331 (PMC10921914; doi:10.1158/2767-9764.CRC-23-0331)
Supplement: Supplementary Table 5 — GAN gene RT-PCR primers [file crc-23-0331-s07.docx]

Supplementary Table 5. GAN gene RT-PCR primers

| Exon | Exon location | Genomic location | Primer sequence | Product size (genomic) | Annealing temperature |
| --- | --- | --- | --- | --- | --- |
| 1 - 3 | 120 - 762 | 5120 - 44771 | 5’-GACCCGTCGGCAGAGGAG-3’  5’-TGTGCTATCCATCGAATTACTCG-3’ | 643 (39650) | 58 |
| 3 - 7 | 627 - 1326 | 44636 - 53920 | 5’-ATTTCCGAGACGTCAGCAGC-3’  5’-CACTCCATGGAAATCAGCTCCT-3’ | 700 (9283) | 60 |
| 7 - 11 | 1284 - 1975 | 53878 - 67664 | 5’-TGGGAGGAGAGGATGGTGAA-3’  5’-GGTCAGGATCTCGCACTCTG-3’ | 692 (13785) | 59 |
| 1 - 11 | 16 - 2015 | 5016 - 67704 | 5’-CGGGGGCTCCAGCTTCTG-3’  5’-TCTCTGCTCTCCCTTTCGGA-3’ | 2000 (62688) | 60 |
| β-actin | 305 - 868 |  | 5’-CTGGGACGACATGGAGAAAA-3’  5’-AAGGAAGGCTGGAAGAGTGC-3’ | 562 (1005) | 52 |

TaqMan gene expression (RT-qPCR) primers from Applied Biosystems

| Assay ID | Gene symbol | Part number | Exon boundary |
| --- | --- | --- | --- |
| Hs00923894_m1 | CDKN2A (p16) | 4453320 | 2-3 |
| Hs00222775_m1 | GAN | 4448892 | 2-3 |
| [Hs00958113_g1](https://www.thermofisher.com/taqman-gene-expression/product/Hs00958113_g1?CID=&ICID=&subtype=) | VIM (vimentin) | [4331182](https://www.thermofisher.com/order/catalog/product/4331182) | 6-7 |
| [Hs01023895_m1](https://www.thermofisher.com/taqman-gene-expression/product/Hs01023895_m1?CID=&ICID=&subtype=) | CDH1 (e-cadherin) | 4331182 | 6-7 |
| [Hs00765553_m1](https://www.thermofisher.com/taqman-gene-expression/product/Hs00765553_m1?CID=&ICID=&subtype=) | CCND1 (cyclin D1) | 4331182 | 3-4 |
| Hs03929097_g1 | GAPDH | 4453320 | 9-9 |
