# Supplementary material for: Gigaxonin Suppresses Epithelial-to-Mesenchymal Transition of Human Cancer Through Downregulation of Snail
Source: Cancer Res Commun. 2024 Mar 8;4(3):706–22. doi: 10.1158/2767-9764.CRC-23-0331 (PMC10921914; doi:10.1158/2767-9764.CRC-23-0331)
Supplement: Supplementary Table 6 — CRISPR-Cas9 oligonucleotides [file crc-23-0331-s08.docx]

Supplementary Table 6. CRISPR-Cas9 oligonucleotides for the GAN gene

exon 8 SNP T>C conversion

| Sequence | Orientation | Primer sequence |
| --- | --- | --- |
| gRNA1 | Sense  Anti-sense | 5’-CAC CGA GAC TCA AAA AGC TTT CCG T-3’  5’-AAA CAC GGA AAG CTT TTT GAG TCT C-3’ |
| gRNA2 | Sense  Anti-sense | 5’-CAC CGA AGA AAA TCT ACG CCA TGG G-3’  5’-AAA CCC CAT GGC GTA GAT TTT CTT C-3’ |
| gRNA3 | Sense  Anti-sense | 5’-CAC CGT CCG TAG GAG CCT CCA CCC A-3’  5’-AAA CTG GGT GGA GGC TCC TAC GGA C-3’ |
| gRNA4 | Sense  Antisense | 5’-CAC CGC CAT GGG TGG AGG CTC CTA-3’  5’-AAA CTA GGA GCC TCC ACC CAT GGC-3’ |
| Sequencing primers | px459 U6 – Forward  px459 - Reverse | 5’-ACT ATC ATA TGC TTA CCG TAA C-3’  5’-GTC TGC AGA ATT GGC GCA CGC G-3’ |
