# Supplementary material for: Gigaxonin Suppresses Epithelial-to-Mesenchymal Transition of Human Cancer Through Downregulation of Snail
Source: Cancer Res Commun. 2024 Mar 8;4(3):706–22. doi: 10.1158/2767-9764.CRC-23-0331 (PMC10921914; doi:10.1158/2767-9764.CRC-23-0331)
Supplement: Supplementary Table 7 — Neurodegenerative diseases and cancer [file crc-23-0331-s09.docx]

Supplementary Table 7. Neurodegenerative diseases and cancer

| Disease | Protein (Gene) | Gene Location | Protein Location | Biochemical function | Life expectancy | Relationship to cancer |
| --- | --- | --- | --- | --- | --- | --- |
| Alzheimer’s | Apoprotein E (APOE) | Ch19q13.2 | Nucleus | FAT binding apolipoprotein  Interacts with LDL receptor, Mediates cholesterol metabolism | 8-11 years after diagnosis | Inversely expressed in cancer, many factors upregulated in cancer are downregulated^1^. |
| Ataxia-telangiectasia | Ataxia-telangiectasia mutated (ATM) | Ch11q23 | Nucleus | Protein Kinase, DNA damage repair (DDR) | 19-25 years | ATM is involved in early recognition and response to double strand DNA breaks.  ATM mutations increase frequency and mortality of human cancers including breast and digestive cancers^2,3^. |
| Fredrich’s Ataxia | Frataxin (FXN) | Ch9q13-21.1 | Mitochondrion | Function not clear, proposed to be involved in the assembly of iron-sulfur clusters | 40-50 years | Frataxin protects tumor cells against oxidative stress through glutathione peroxidase and apoptosis but also acts as a tumor suppressor.  Overexpression of frataxin reduces malignant transformation induced by ROS in nude mice^4,5^. |
| Huntington’s | Huntingtin (HTT) | Ch4p16.3 | Nucleus and cytoplasm | Modulates cellular adhesion and differentiation, unclear mechanism | 10-30 years | Reduced cancer incidence in Huntington’s patients^6,7^. |
| Parkinson’s | Parkin (PRKN),  Alpha synuclein (SNCA),  Leucine rich repeat kinase 2 (LRRK2) | Ch6q26  4q22.1  12p11.2–q13.1 | Nucleus and cytoplasm | Parkin - E3 ubiquitin ligase  Precise function unknown  Alpha synuclein - Regulates synaptic vesicle trafficking.  LRRK2 – a kinase | 60+ years | Parkin reduces incidence of cancer, likely inhibits tumorigenesis.  Alpha-synuclein is probably indicated in cancers, low expression in lung carcinoma,  Loss of LRRK2 knockout model in murine lung cancer model promotes tumorigenesis^8–11^. |
| Motor neuron (Amyotrophic lateral sclerosis) | Superoxide dismutase (SOD1) | Ch21q22.1 | Mitochondrion | Binding Mn/Cu/Zn ions, destruction of free superoxide radicals | 2-5 years after diagnosis | Mn-SOD1 overexpression acts as a tumor suppressor protein in human cancers^12^. |
