# Supplementary material for: Gigaxonin Suppresses Epithelial-to-Mesenchymal Transition of Human Cancer Through Downregulation of Snail
Source: Cancer Res Commun. 2024 Mar 8;4(3):706–22. doi: 10.1158/2767-9764.CRC-23-0331 (PMC10921914; doi:10.1158/2767-9764.CRC-23-0331)
Supplement: Supplementary Table 8 — Antibody details [file crc-23-0331-s10.docx]

Supplementary Table 8. Antibody details

| Antibody | Source | Dilution | Procedure used | Company | Catalog No. | City and the State |
| --- | --- | --- | --- | --- | --- | --- |
| Actin | Mouse | 1:1000 | Western blotting | Santacruz Biotechnology | SC-8432 | Dallas, TX |
| Cyclin D1 | Mouse | 1:1000 | Western blotting | Upstate Technology/Sigma Millipore | 05-362 | Temecula, CA |
| E-cadherin | Rabbit | 1:1000  1:200 | Western blotting  Immunohistochemistry | Cell Signaling Technology | 3195 (24E10) | Danvers, MA |
| Gigaxonin | Mouse | 1:1000 | Western blotting | Santa Cruz Biotechnology | SC-376173 | Dallas, TX |
| Gigaxonin | Mouse | 1:100 | Western blotting | From Dr. P. Bomont | Gift | INMG, Lyon, France |
| N-cadherin | Rabbit | 1:1000 | Western blotting | Cell Signaling Technology | 13116 | Danvers, MA |
| NFκB | Rabbit | 1:1000 | Western blotting | Santa Cruz Biotechnology | SC-109 | Dallas, TX |
| p16 | Rabbit | 1:1000 | Western blotting | Abcam | ab108349 | Cambridge, MA |
| SNAIL | Rabbit  Mouse | 1:1000  1;100 | Western blotting Immunohistochemistry | Novus Biologicals  Cell Signaling technology | NBP-229626  3895 (L70G2) | Centennial, CO  Danvers, MA |
| SOX2 | Rabbit | 1:1000 | Western blotting | Cell Signaling Technology | 14962 | Danvers, MA |
| TWIST | Mouse | 1:500 | Western blotting | Santacruz Biotechnology | SC-81417 | Dallas, TX |
| Ubiquitin | Mouse | 1:1000 | Western blotting | Cell Signaling Technology | 3936 | Danvers, MA |
| Vimentin | Rabbit | 1:1000 | Western blotting | Cell Signaling Technology | 5741 | Danvers, MA |
| Zeb | Mouse | 1:1000 | Western blotting | ebioscience | 14-9741-80 | San Diego, CA |
| Normal rabbit IgG | Rabbit | 1:100 | Immunoprecipitation | R&D Biosystems | AB-105-C | Minneapolis, MN |
| anti-rabbit HRP (secondary ab) | Mouse | 1:2000 | Western blotting | Santacruz Biotechnology | Sc-2357 | Dallas, TX |
| anti-mouse HRP (secondary ab) | Recombinant mouse IgG_k_ | 1:2000 | Western blotting | Santacruz Biotechnology | Sc-516102 | Dallas, TX |
