# Supplementary material for: Gigaxonin Suppresses Epithelial-to-Mesenchymal Transition of Human Cancer Through Downregulation of Snail
Source: Cancer Res Commun. 2024 Mar 8;4(3):706–22. doi: 10.1158/2767-9764.CRC-23-0331 (PMC10921914; doi:10.1158/2767-9764.CRC-23-0331)
Supplement: Supplementary Figure 1 — Mitochondrial gene authentication primers [file crc-23-0331-s11.docx]

Supplementary Figure 1

Mitochondrial cell authentication primers: Ref - Andrew et al Nat Genet 1999, 23:147

Primers used were HVR1 F 5’- TTTGGTATTTTCGTCTGG - 3’, HVR1 R 5’- TACATAAACTGTGGGGGG - 3’; HVR2 F 5’- TAATTTAAACTATTCTCT - 3’, HVR2 R 5’- CAGATGTCGGATACAGTT - 3’.

HVR1 sequence: 50-585bp (PCR product of 535bp)

GATCACAGGTCTATCACCCTATTAACCACTCACGGGAGCTCTCCATGCA

50 TTTGGTATTTTCGTCTGGGGGGTATGCACGCGATAGCATTGCGAGACGCTGGAGCCGGAGCACCCTATGTCGCAGTATCTGTCTTTGATTCCTGCCTCAT

150

CCTATTATTTATCGCACCTACGTTCAATATTACAGGCGAACATACTTACTAAAGTGTGTTAATTAATTAATGCTTGTAGGACATAATAATAACAATTGAA

250

TGTCTGCACAGCCACTTTCCACACAGACATCATAACAAAAAATTTCCACCAAACCCCCCC--T-CCCCCGCTTCTGGCCACAGCACTTAAACACATCTCTGCC

350

AAACCCCAAAAACAAAGAACCCTAACACCAGCCTAACCAGATTTCAAATTTTATCTTTTGGCGGTATGCACTTTTAACAGTCACCCCCCAACTAACACAT

450

TATTTTCCCCTCCCACTCCCATACTACTAATCTCATCAATACAACCCCCGCCCATCCTACCCAGCACACACACACCGCTGCTAACCCCATACCCCGAACC

550

AACCAAACCCCAAAGACACCCCCCACAGTTTATGTA 585

HVR2 Primer sequences: 16005-16504 (PCR product of 499bp)

16005 TAATTTAAACTATTCTCTGTTCTTTCATGGGGAAGCAGATTTGGGTACCACCCAAGTATTGACTCACCCATCAACAACCGCTATGTATTTCGTACATTAC

16105 TGCCAGCCACCATGAATATTGTACGGTACCATAAATACTTGACCACCTGTAGTACATAAAAACCCAATCCACATCAAAACCCCCTCCC-CATGCTTACAAG

16205 CAAGTACAGCAATCAACCCTCAACTATCACACATCAACTGCAACTCCAAAGCCACCCCTCACCCACTAGGATACCAACAAACCTACCCACCCTTAACAGT

16305 ACATAGTACATAAAGCCATTTACCGTACATAGCACATTACAGTCAAATCCCTTCTCGTCCCCATGGATGACCCCCCTCAGATAGGGGTCCCTTGACCACC

16405

ATCCTCCGTGAAATC-AATATCCCGCACAAGA-GTGCTACTCTCCTCGCTCCGGGCCCATAACACTTGGGGGTAGCTAAAGTGAACTGTATCCGACATCTG

16505

GTTCCTACTTCAGGGTCATAAAGCCTAAATAGCCCACACGTTCCCCTTAAATAAGACATCACGATG
