# Supplementary material for: Gigaxonin Suppresses Epithelial-to-Mesenchymal Transition of Human Cancer Through Downregulation of Snail
Source: Cancer Res Commun. 2024 Mar 8;4(3):706–22. doi: 10.1158/2767-9764.CRC-23-0331 (PMC10921914; doi:10.1158/2767-9764.CRC-23-0331)
Supplement: Supplementary Figure 2 — Morphology of fibroblast and cancer cell lines [file crc-23-0331-s12.pptx]

## Slide 1
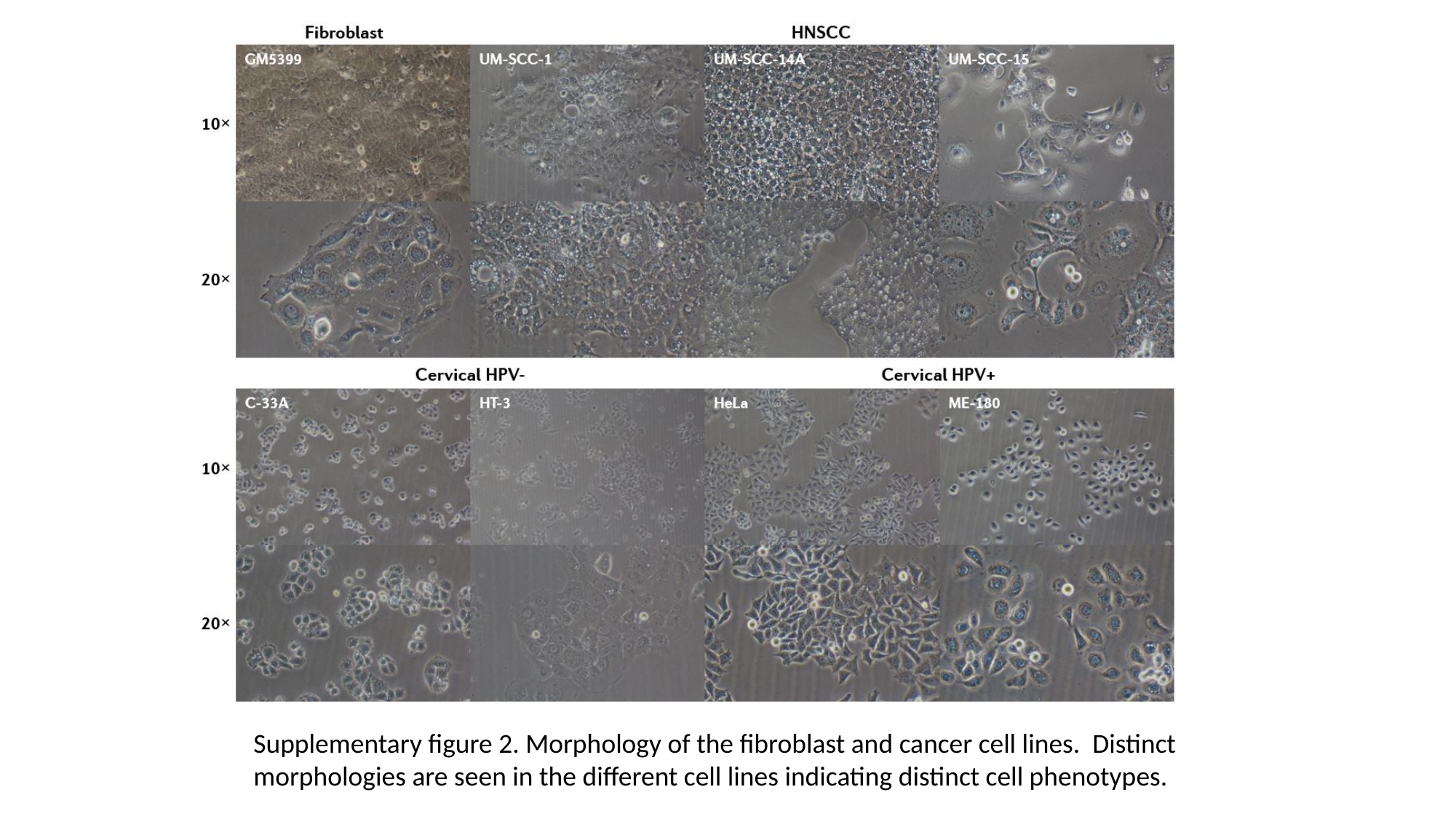

Supplementary figure 2. Morphology of the fibroblast and cancer cell lines. Distinct
morphologies are seen in the different cell lines indicating distinct cell phenotypes.
