# Supplementary material for: Gigaxonin Suppresses Epithelial-to-Mesenchymal Transition of Human Cancer Through Downregulation of Snail
Source: Cancer Res Commun. 2024 Mar 8;4(3):706–22. doi: 10.1158/2767-9764.CRC-23-0331 (PMC10921914; doi:10.1158/2767-9764.CRC-23-0331)
Supplement: Supplementary Figure 3 — SNP detection and CDKN2A and GAN expression in cancer cell lines [file crc-23-0331-s13.pptx]

## Slide 1
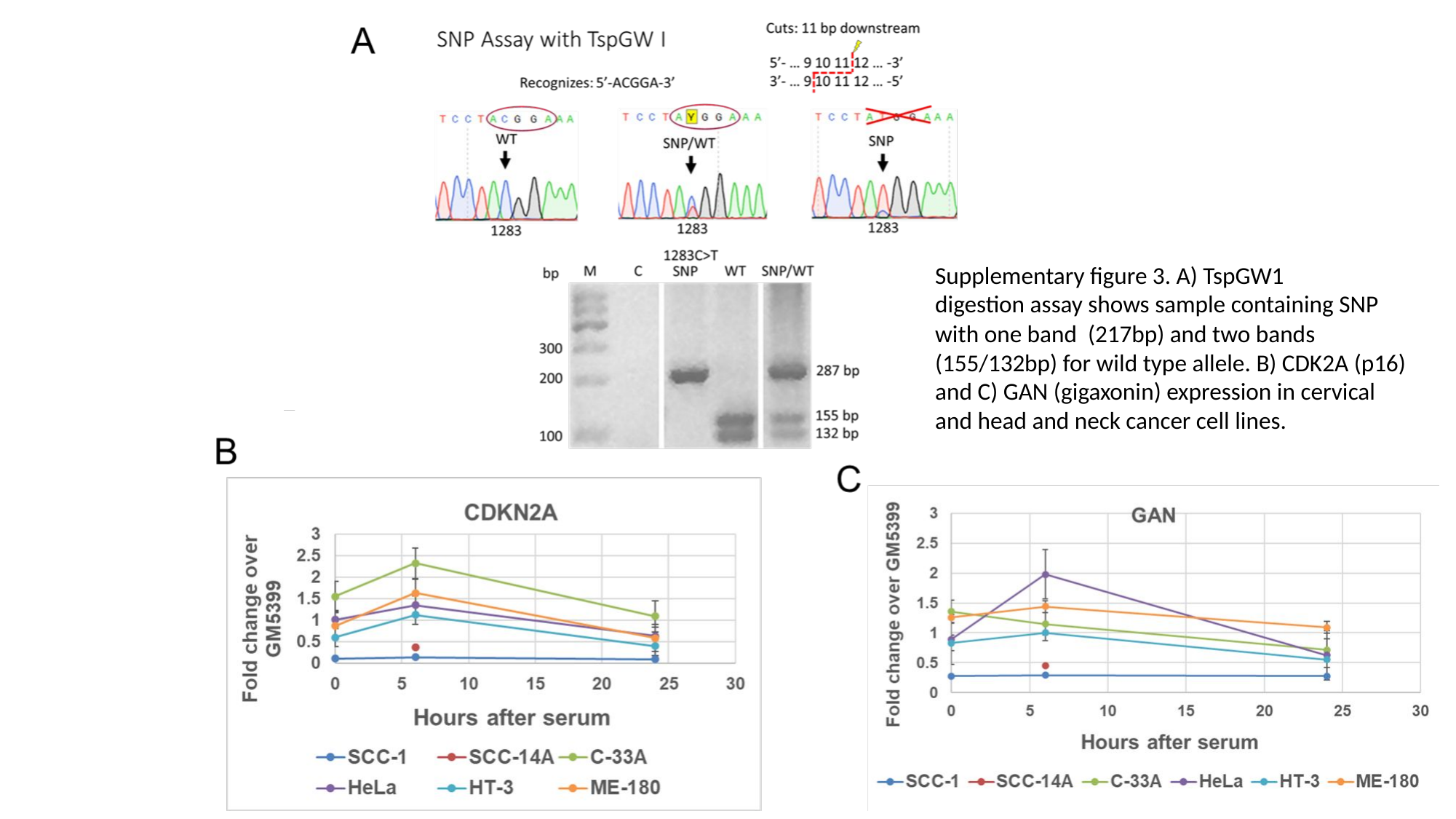

Supplementary figure 3. A) TspGW1
digestion assay shows sample containing SNP
with one band (217bp) and two bands (155/132bp) for wild type allele. B) CDK2A (p16) and C) GAN (gigaxonin) expression in cervical and head and neck cancer cell lines.
