# Supplementary material for: Gigaxonin Suppresses Epithelial-to-Mesenchymal Transition of Human Cancer Through Downregulation of Snail
Source: Cancer Res Commun. 2024 Mar 8;4(3):706–22. doi: 10.1158/2767-9764.CRC-23-0331 (PMC10921914; doi:10.1158/2767-9764.CRC-23-0331)
Supplement: Supplementary Figure 4 — MTT and sift agar assay and Snapshot view of exon 8 of C33A and HT3 cell lines [file crc-23-0331-s14.pptx]

## Slide 1
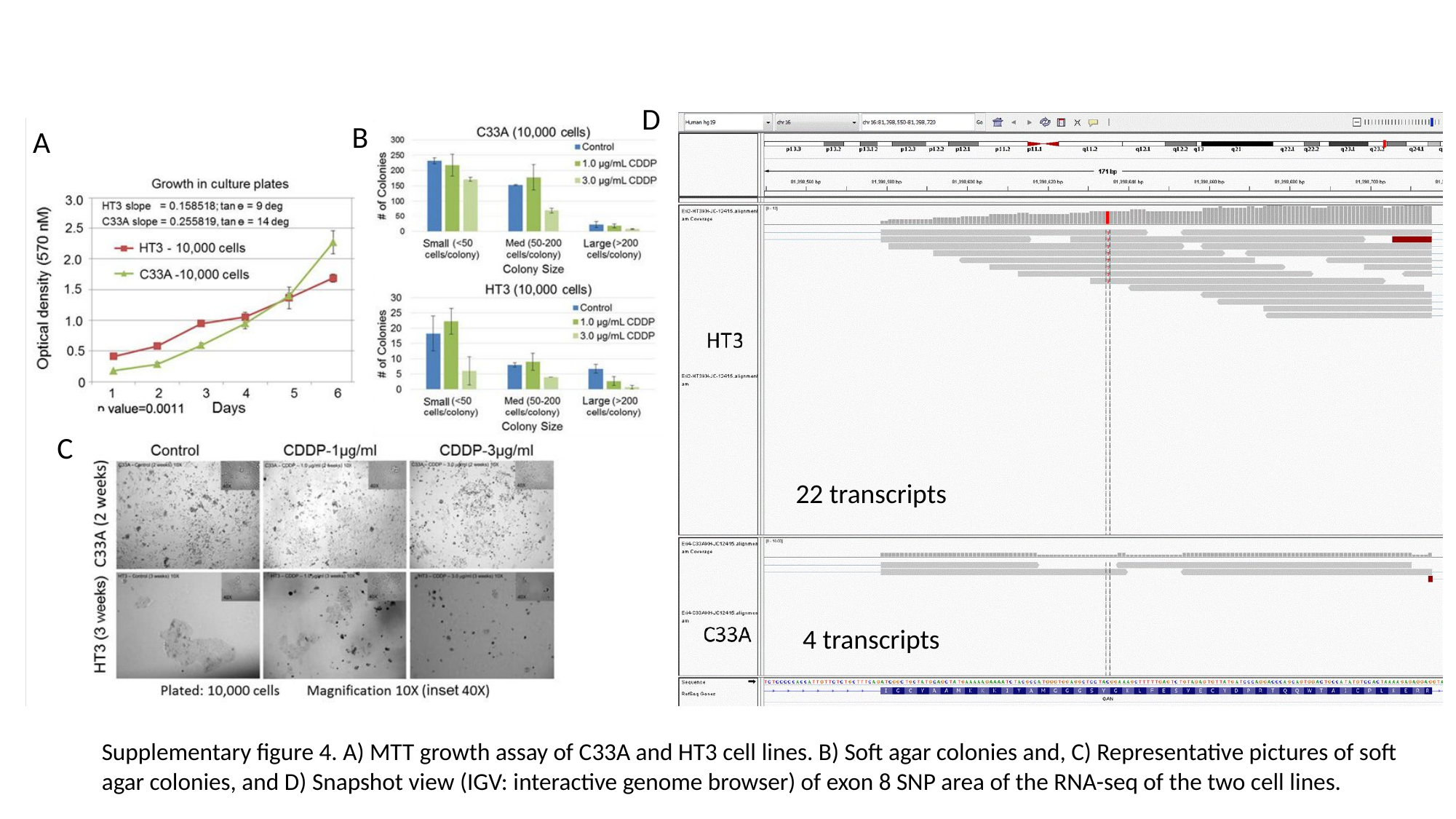

D
B
A
C
22 transcripts
4 transcripts
Supplementary figure 4. A) MTT growth assay of C33A and HT3 cell lines. B) Soft agar colonies and, C) Representative pictures of soft agar colonies, and D) Snapshot view (IGV: interactive genome browser) of exon 8 SNP area of the RNA-seq of the two cell lines.
