# Supplementary material for: Gigaxonin Suppresses Epithelial-to-Mesenchymal Transition of Human Cancer Through Downregulation of Snail
Source: Cancer Res Commun. 2024 Mar 8;4(3):706–22. doi: 10.1158/2767-9764.CRC-23-0331 (PMC10921914; doi:10.1158/2767-9764.CRC-23-0331)
Supplement: Supplementary Figure 5 — Representative soft agar colonies of ME180, HeLa, and Siha cell lines [file crc-23-0331-s15.pptx]

## Slide 1
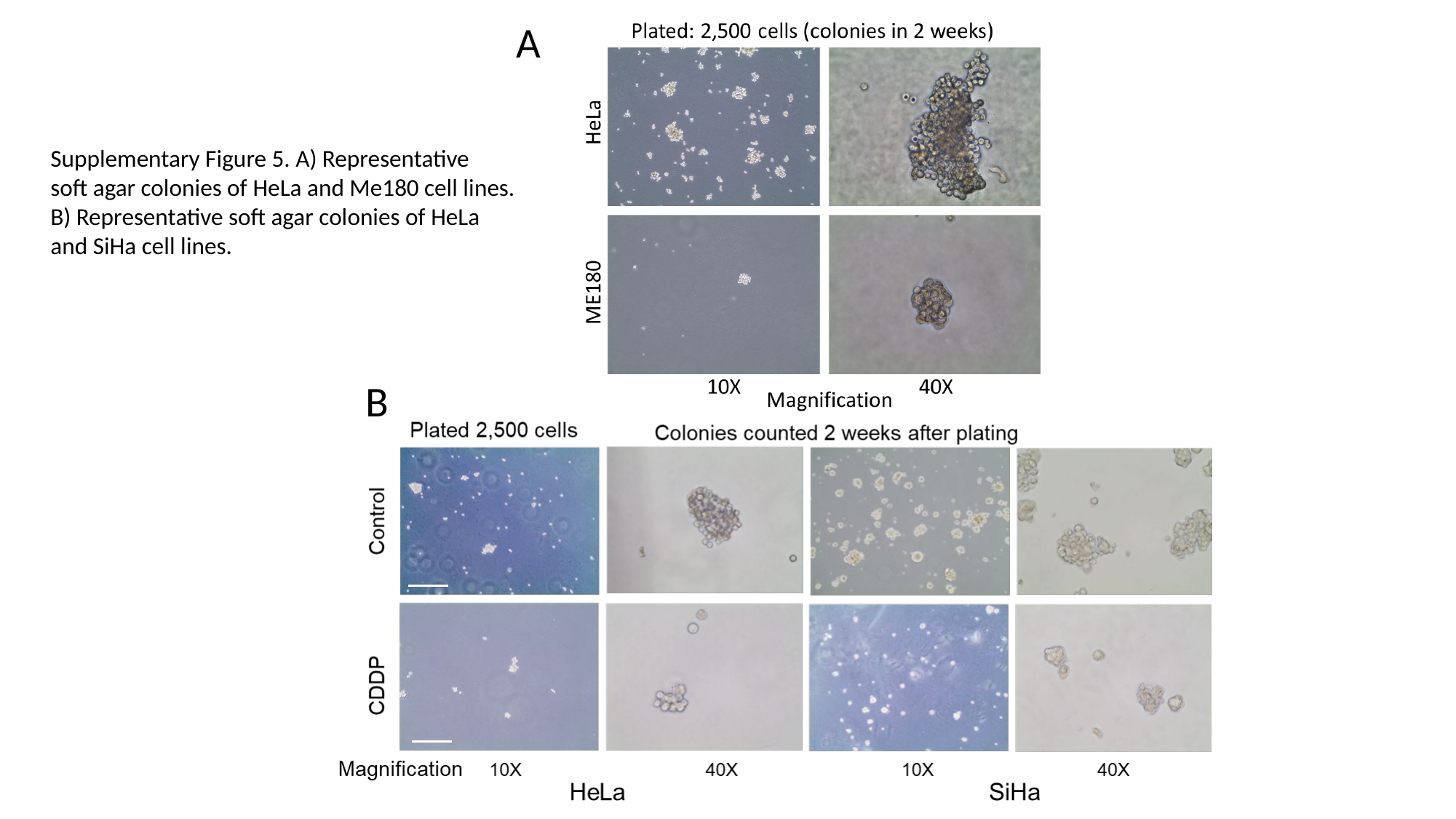

A
Supplementary Figure 5. A) Representative
soft agar colonies of HeLa and Me180 cell lines.
B) Representative soft agar colonies of HeLa
and SiHa cell lines.
100μM
B
