# Supplementary material for: Gigaxonin Suppresses Epithelial-to-Mesenchymal Transition of Human Cancer Through Downregulation of Snail
Source: Cancer Res Commun. 2024 Mar 8;4(3):706–22. doi: 10.1158/2767-9764.CRC-23-0331 (PMC10921914; doi:10.1158/2767-9764.CRC-23-0331)
Supplement: Supplementary Figure 6 — Incucyte live cell assay of cisplatin treated ME180 cells [file crc-23-0331-s16.pptx]

## Slide 1
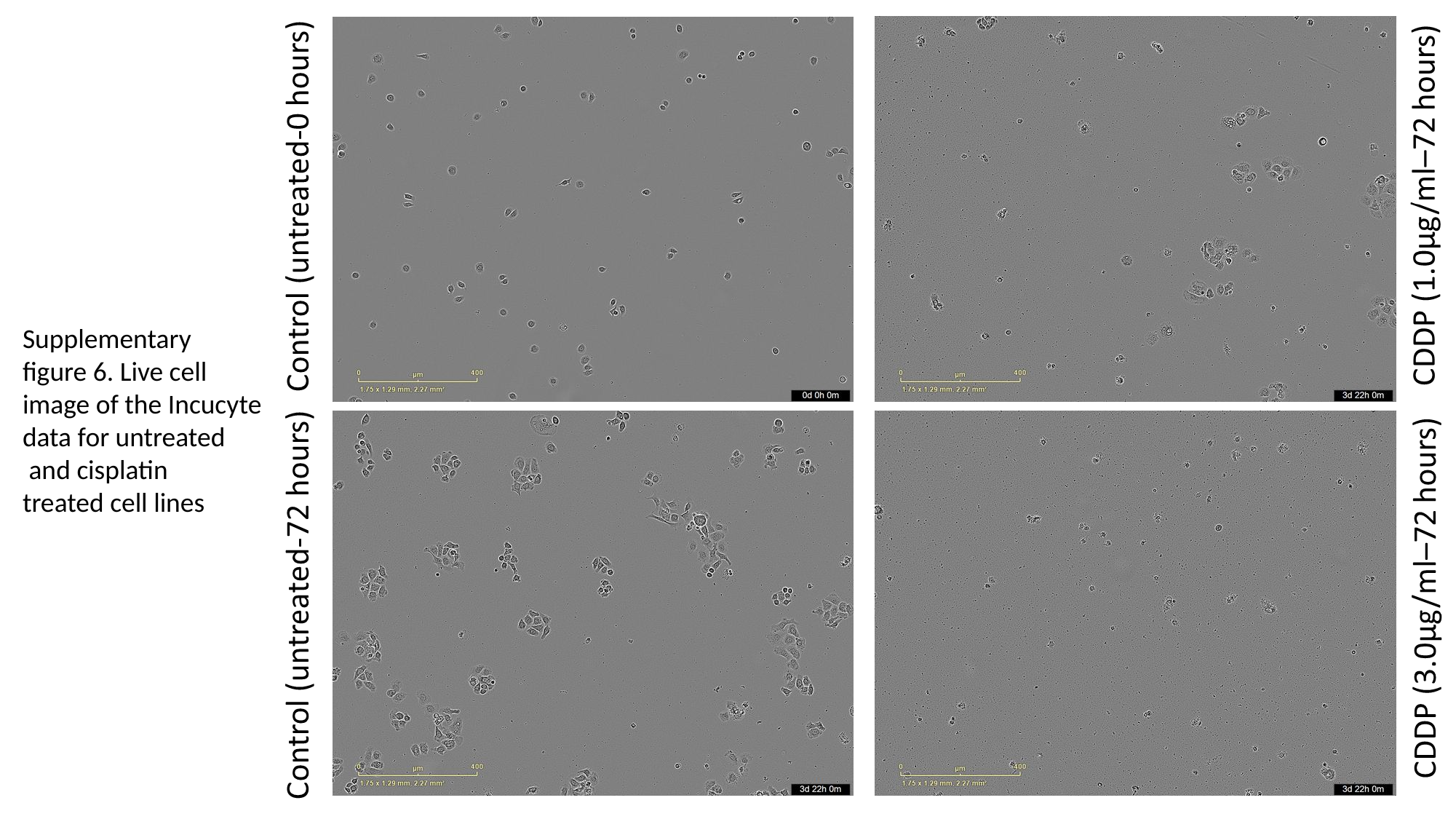

Supplementary
figure 6. Live cell
image of the Incucyte
data for untreated
 and cisplatin
treated cell lines
