# Supplementary material for: Gigaxonin Suppresses Epithelial-to-Mesenchymal Transition of Human Cancer Through Downregulation of Snail
Source: Cancer Res Commun. 2024 Mar 8;4(3):706–22. doi: 10.1158/2767-9764.CRC-23-0331 (PMC10921914; doi:10.1158/2767-9764.CRC-23-0331)
Supplement: Supplementary Figure 7 — ME180 and CRISPR-Cas9 clones [file crc-23-0331-s17.pptx]

## Slide 1
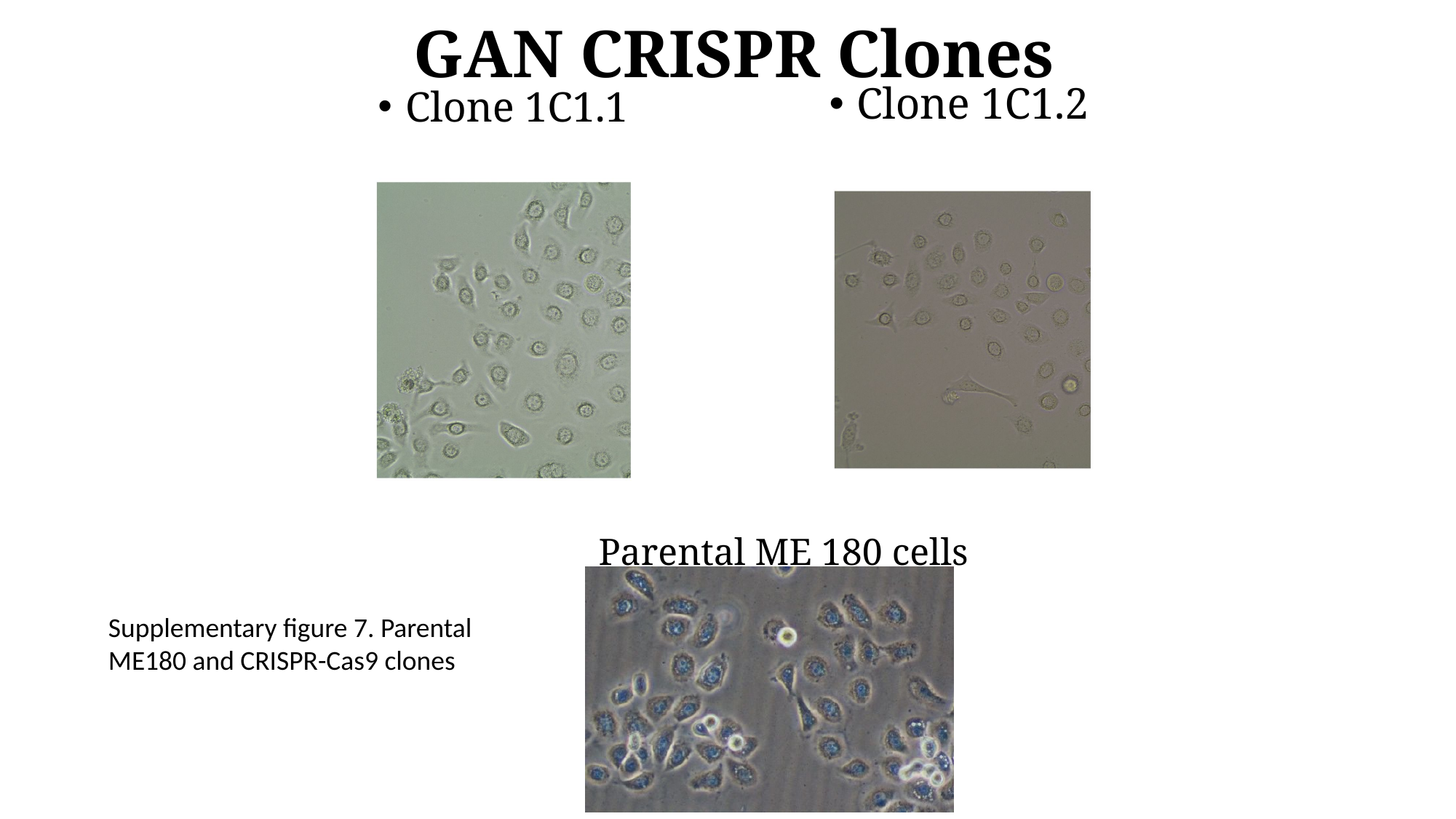

# GAN CRISPR Clones
Clone 1C1.2
Clone 1C1.1
Parental ME 180 cells
Supplementary figure 7. Parental
ME180 and CRISPR-Cas9 clones
