# Supplementary figures and images for: Gigaxonin Suppresses Epithelial-to-Mesenchymal Transition of Human Cancer Through Downregulation of Snail
Source: Cancer Res Commun. 2024 Mar 8;4(3):706–22. doi: 10.1158/2767-9764.CRC-23-0331 (PMC10921914; doi:10.1158/2767-9764.CRC-23-0331)

## Slide 1
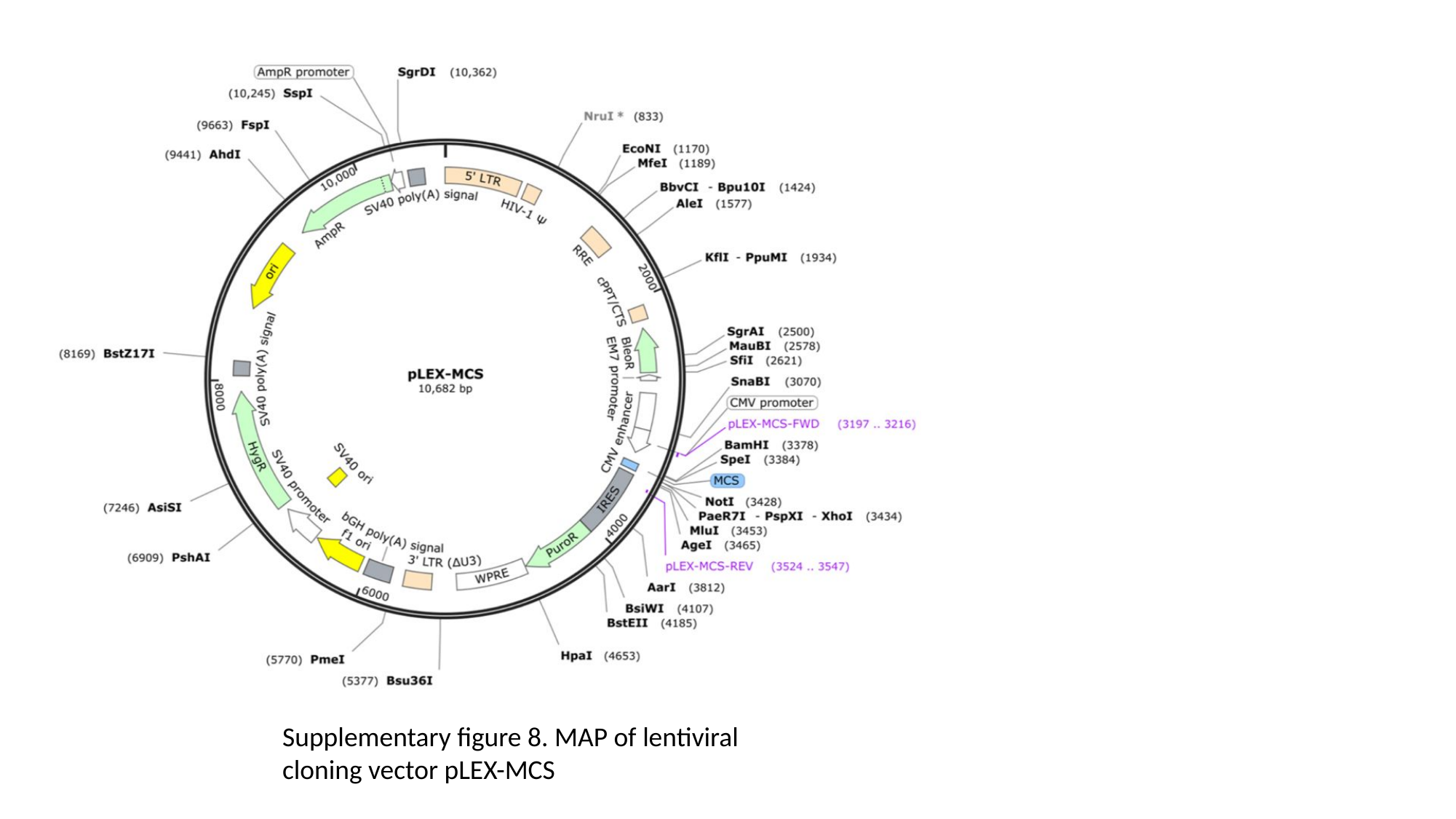

Supplementary figure 8. MAP of lentiviral
cloning vector pLEX-MCS

Supplement: Supplementary Figure 8 — Map of lentiviral cloning vector [file crc-23-0331-s18.pptx]

## Slide 1
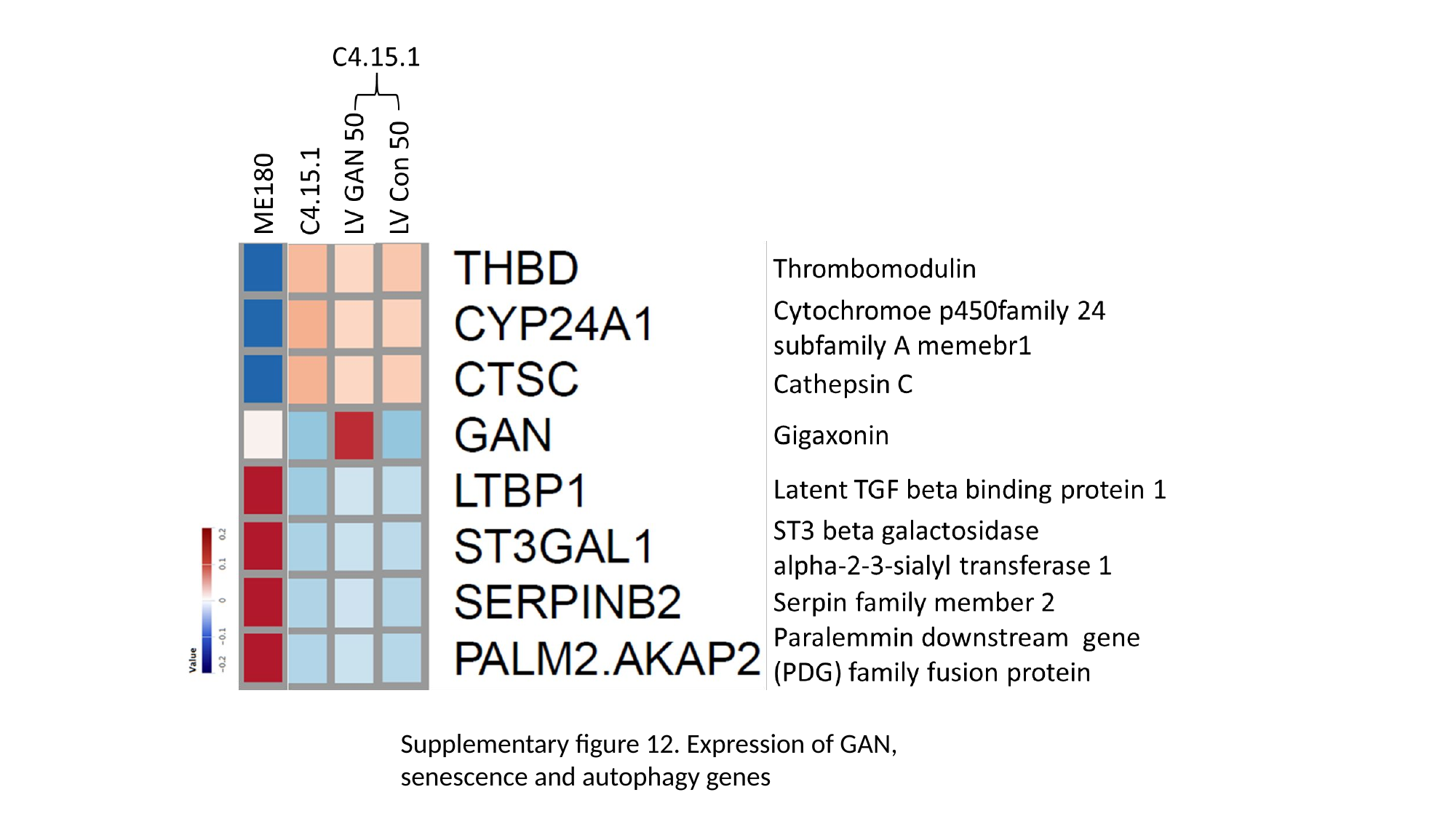

Supplementary figure 12. Expression of GAN,
senescence and autophagy genes

Supplement: Supplementary Figure 12 — expression of GAN, senescence and autophagy genes [file crc-23-0331-s22.pptx]

## Slide 1
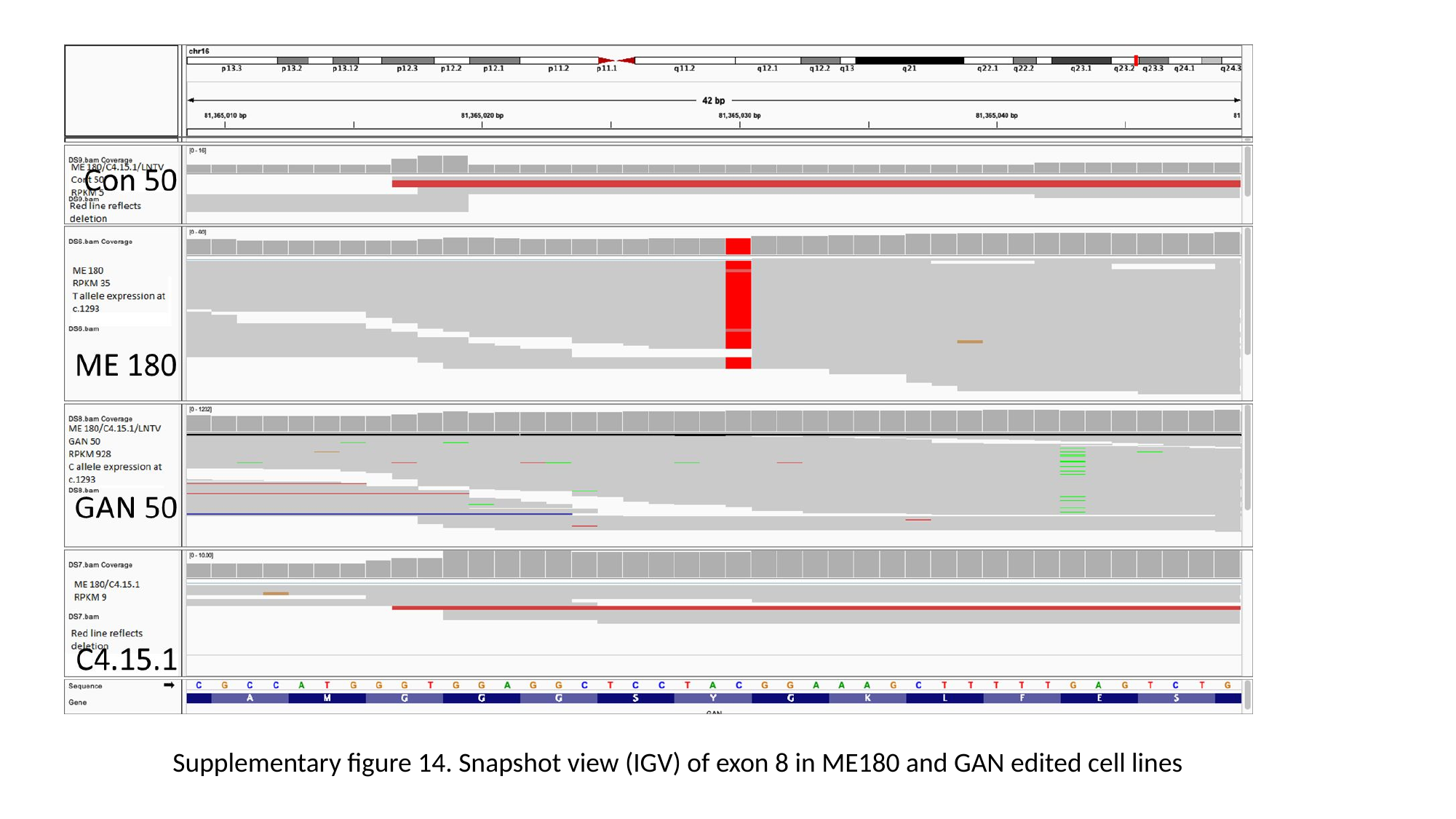

Supplementary figure 14. Snapshot view (IGV) of exon 8 in ME180 and GAN edited cell lines

Supplement: Supplementary Figure 14 — Snapshot view of exon 8 in ME180 and GAN edited cell lines [file crc-23-0331-s24.pptx]

## Slide 1
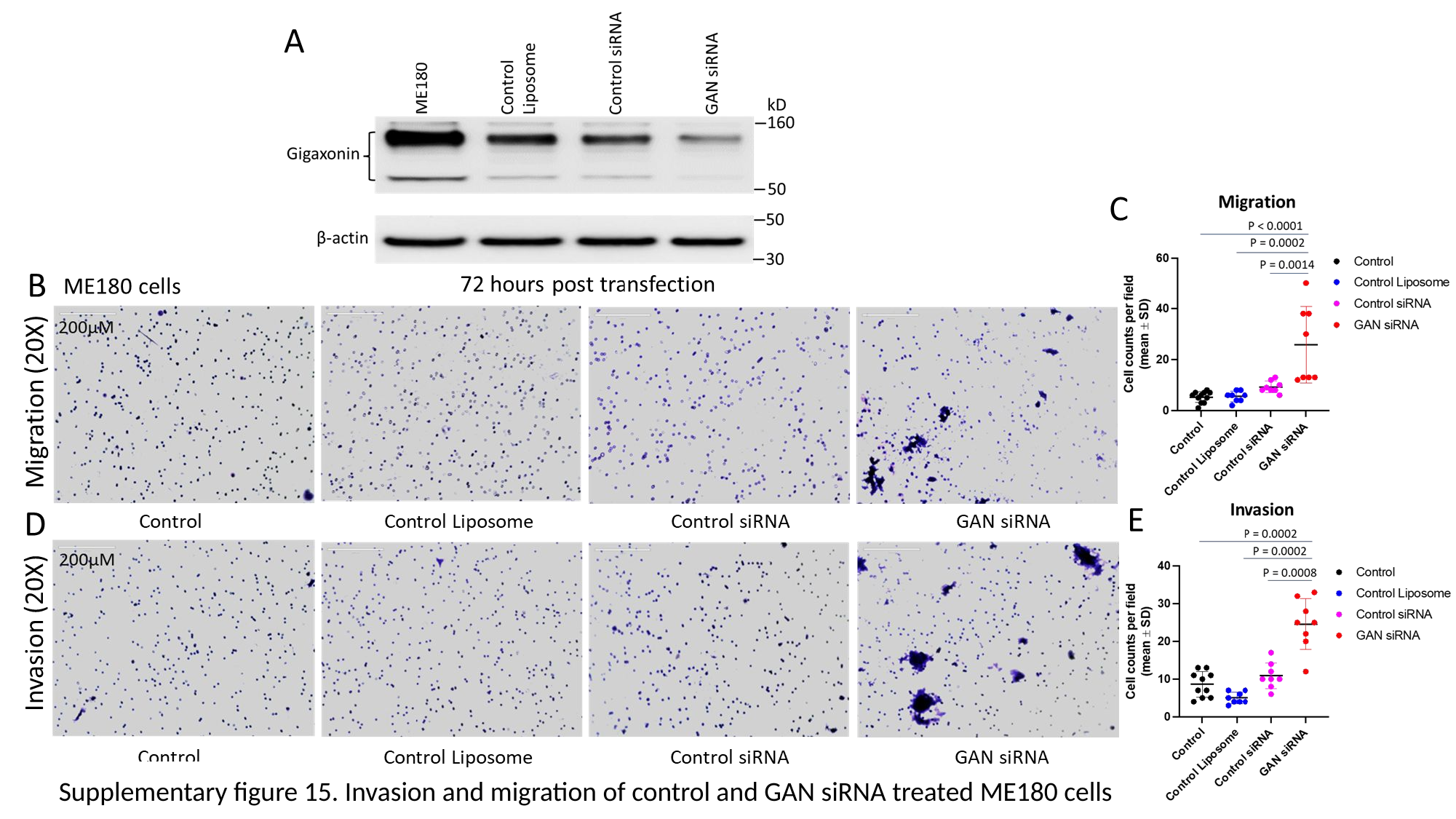

Supplementary figure 15. Invasion and migration of control and GAN siRNA treated ME180 cells

Supplement: Supplementary Figure 15 — Invasion and migration of control and GAN siRNA treated ME180 cells [file crc-23-0331-s25.pptx]

## Slide 1
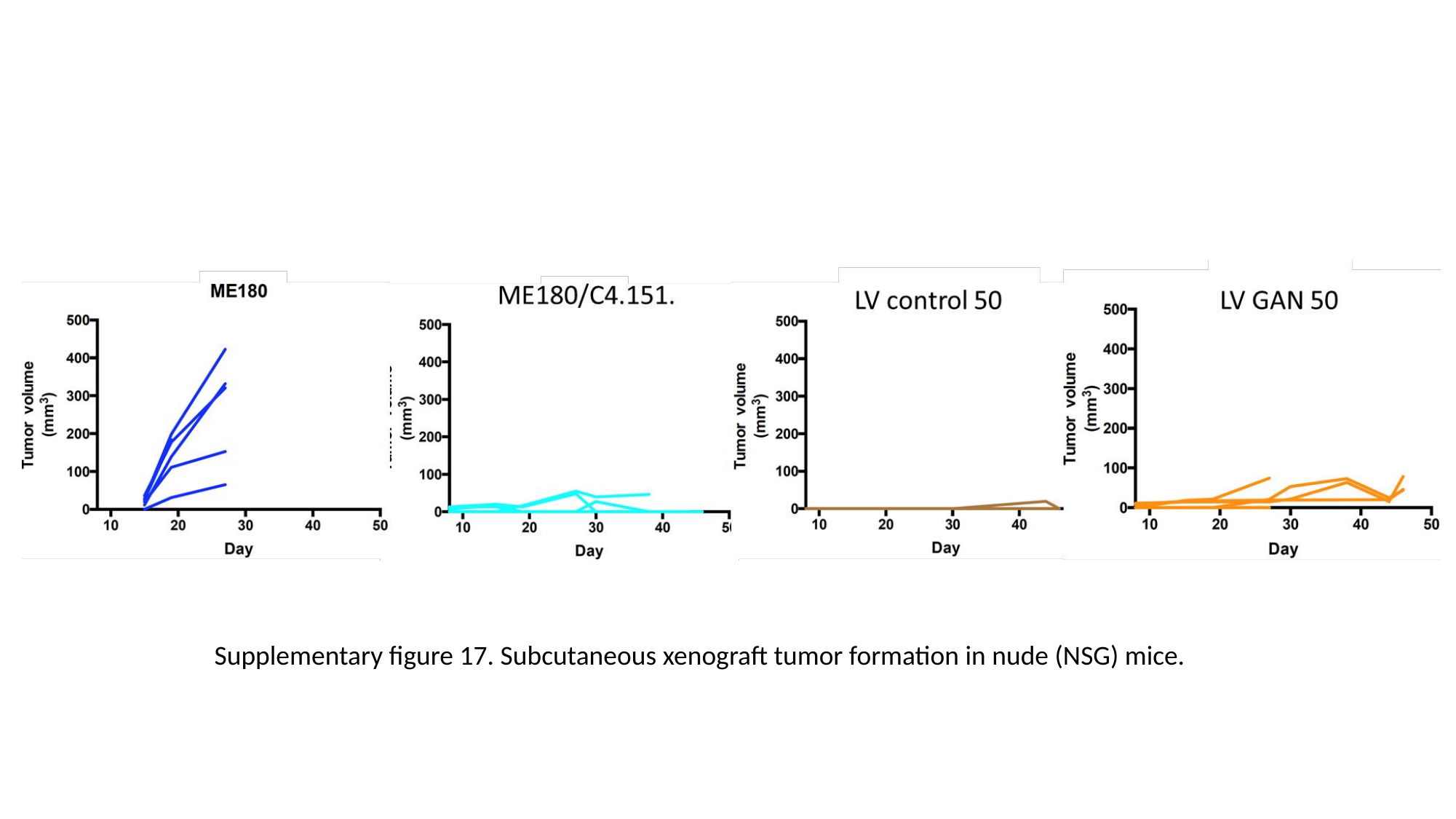

Supplementary figure 17. Subcutaneous xenograft tumor formation in nude (NSG) mice.

Supplement: Supplementary Figure 17 — Subcutaneous xenograft tumor formation in nude (NSG) mice [file crc-23-0331-s27.pptx]

## Slide 1
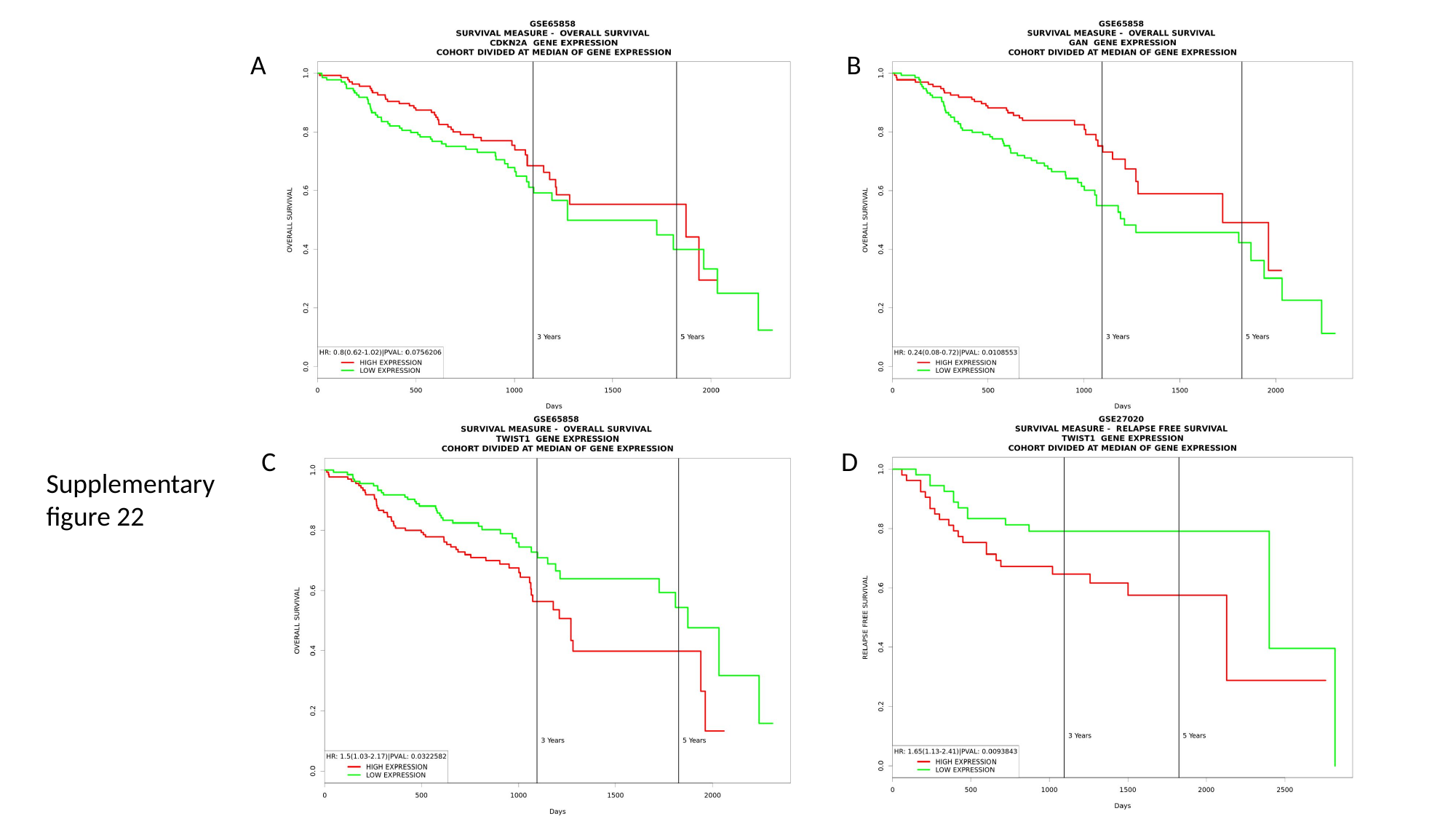

A
B
C
D
Supplementary figure 22

Supplement: Supplementary Figure 22 — Overall survival of head and neck cancer [file crc-23-0331-s32.pptx]
