# Supplementary material for: Gigaxonin Suppresses Epithelial-to-Mesenchymal Transition of Human Cancer Through Downregulation of Snail
Source: Cancer Res Commun. 2024 Mar 8;4(3):706–22. doi: 10.1158/2767-9764.CRC-23-0331 (PMC10921914; doi:10.1158/2767-9764.CRC-23-0331)
Supplement: Supplementary Figure 9 — Synonymous exome duplication sequences in ME180 and GAN edited cell lines [file crc-23-0331-s19.pptx]

## Slide 1
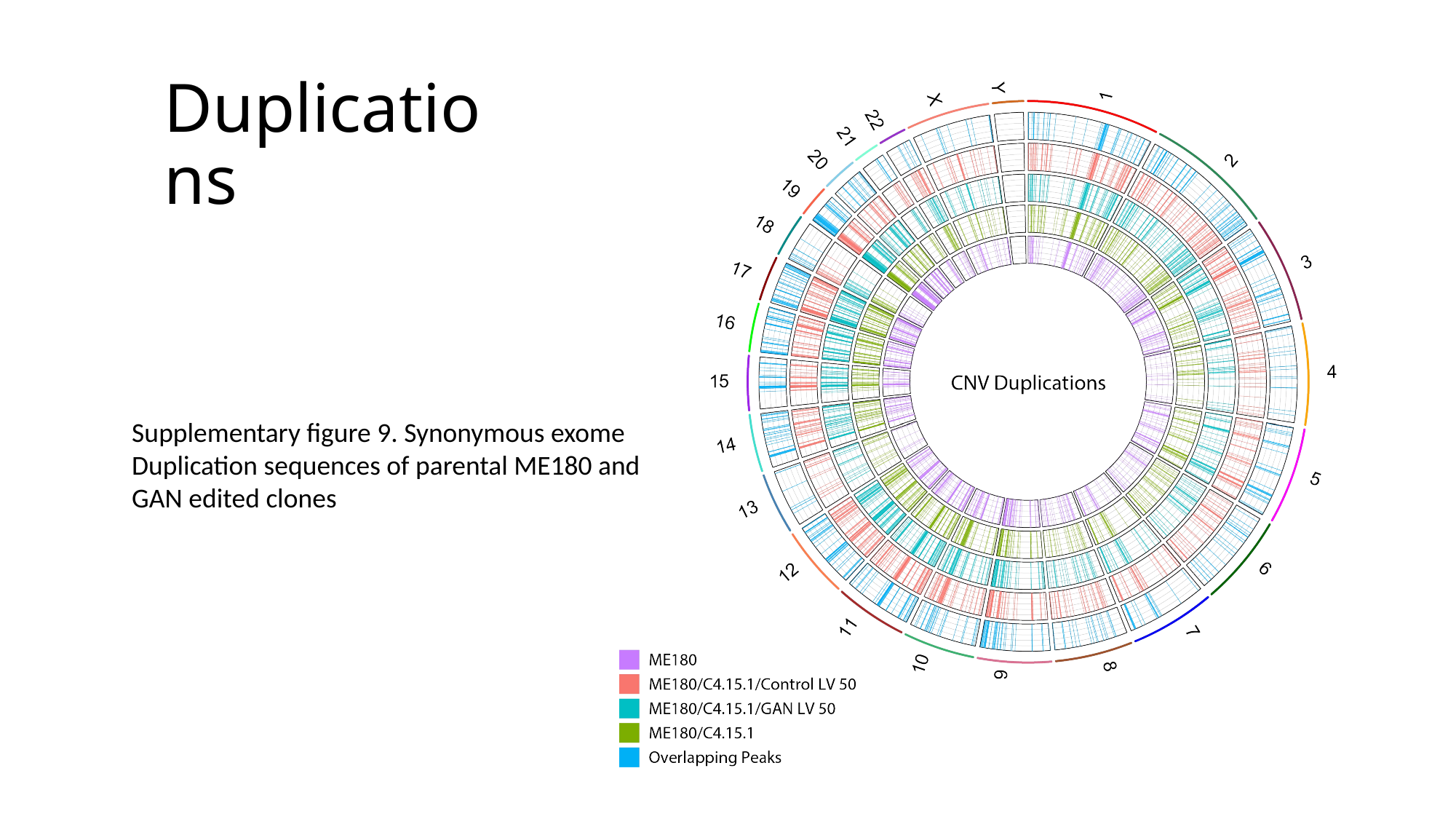

# Duplications
Supplementary figure 9. Synonymous exome
Duplication sequences of parental ME180 and
GAN edited clones
