# Supplementary material for: Gigaxonin Suppresses Epithelial-to-Mesenchymal Transition of Human Cancer Through Downregulation of Snail
Source: Cancer Res Commun. 2024 Mar 8;4(3):706–22. doi: 10.1158/2767-9764.CRC-23-0331 (PMC10921914; doi:10.1158/2767-9764.CRC-23-0331)
Supplement: Supplementary Figure 10 — Synonymous exome SNP and Indel sequences in ME180 and GAN edited cell lines [file crc-23-0331-s20.pptx]

## Slide 1
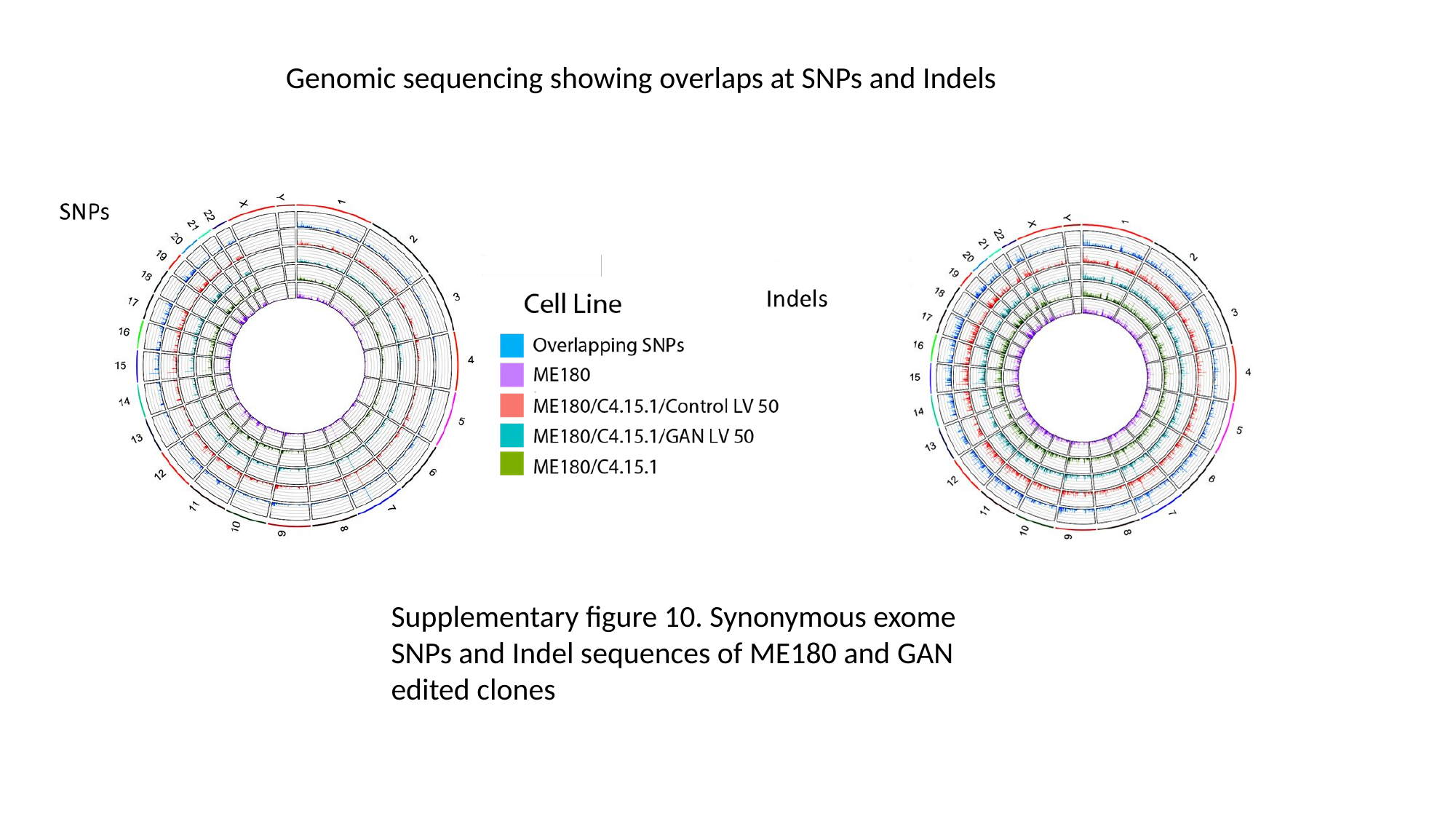

Genomic sequencing showing overlaps at SNPs and Indels
Supplementary figure 10. Synonymous exome SNPs and Indel sequences of ME180 and GAN edited clones
