# Supplementary material for: Gigaxonin Suppresses Epithelial-to-Mesenchymal Transition of Human Cancer Through Downregulation of Snail
Source: Cancer Res Commun. 2024 Mar 8;4(3):706–22. doi: 10.1158/2767-9764.CRC-23-0331 (PMC10921914; doi:10.1158/2767-9764.CRC-23-0331)
Supplement: Supplementary Figure 11 — All non synonymous SNPs in ME180 and GAN edited cell lines [file crc-23-0331-s21.pptx]

## Slide 1
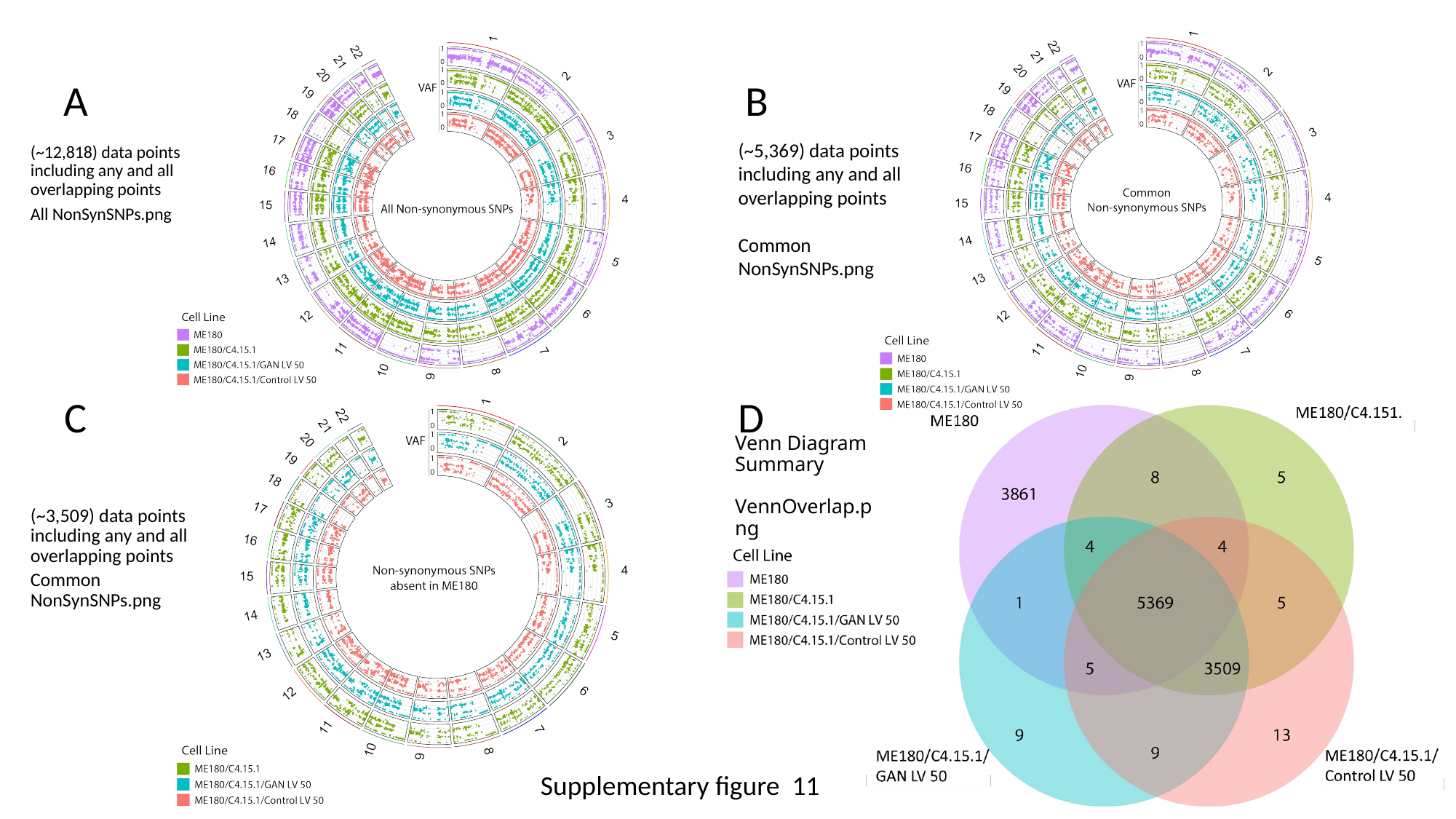

A
B
(~5,369) data points including any and all overlapping points
Common NonSynSNPs.png
(~12,818) data points including any and all overlapping points
All NonSynSNPs.png
C
D
# Venn Diagram Summary VennOverlap.png
(~3,509) data points including any and all overlapping points
Common NonSynSNPs.png
Supplementary figure 11
