# Supplementary material for: Gigaxonin Suppresses Epithelial-to-Mesenchymal Transition of Human Cancer Through Downregulation of Snail
Source: Cancer Res Commun. 2024 Mar 8;4(3):706–22. doi: 10.1158/2767-9764.CRC-23-0331 (PMC10921914; doi:10.1158/2767-9764.CRC-23-0331)
Supplement: Supplementary Figure 13 — RNA differential expression in mE180 and GAN edited cell lines [file crc-23-0331-s23.pptx]

## Slide 1
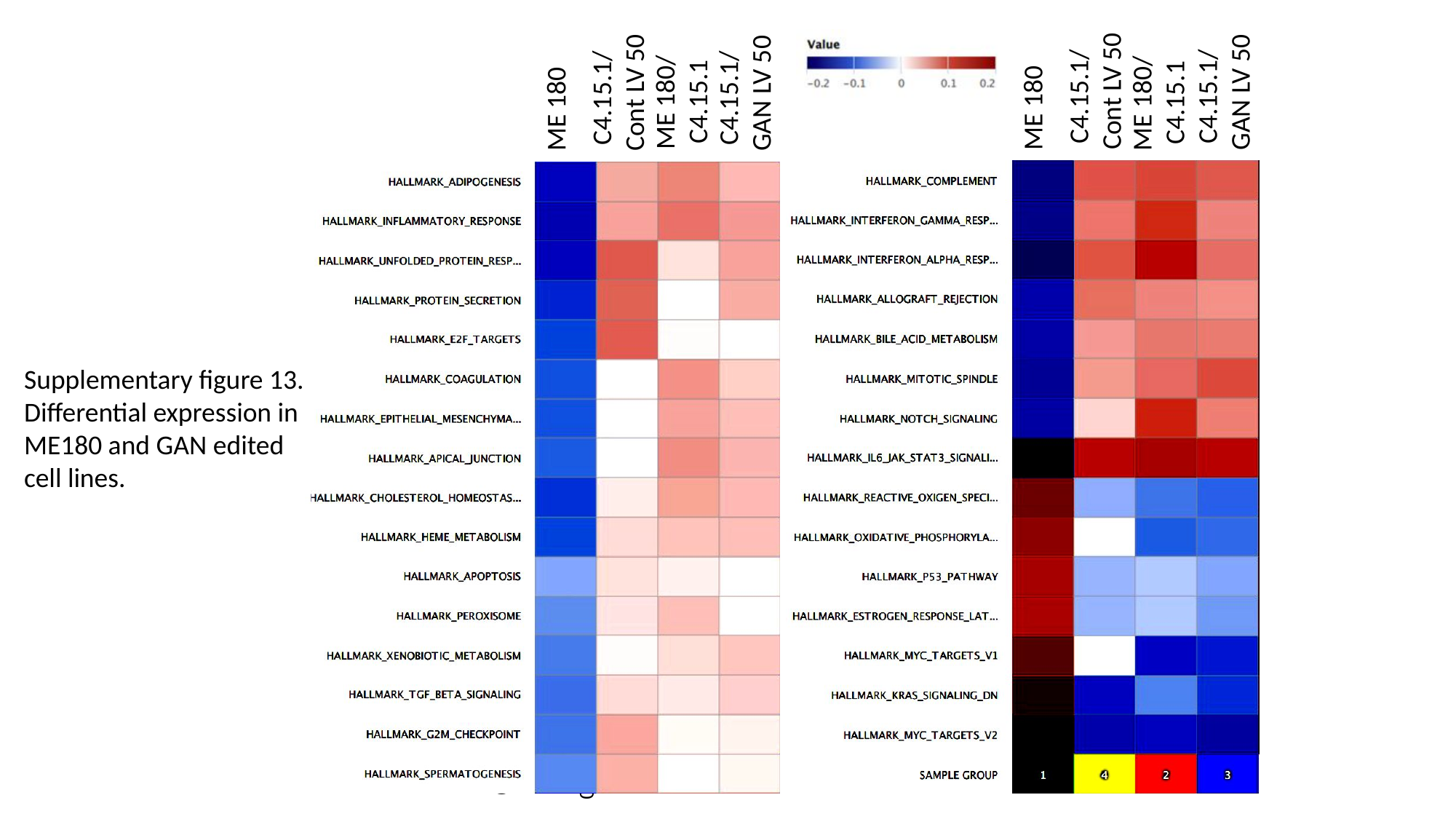

C4.15.1/
Cont LV 50
 C4.15.1/
Cont LV 50
 C4.15.1/
GAN LV 50
 C4.15.1/
GAN LV 50
ME 180/
 C4.15.1
ME 180/
 C4.15.1
ME 180
ME 180
Supplementary figure 13.
Differential expression in
ME180 and GAN edited
cell lines.
Anova
ME180
C4.15.1
LV con 50
LV GAN 50
