# Supplementary material for: Gigaxonin Suppresses Epithelial-to-Mesenchymal Transition of Human Cancer Through Downregulation of Snail
Source: Cancer Res Commun. 2024 Mar 8;4(3):706–22. doi: 10.1158/2767-9764.CRC-23-0331 (PMC10921914; doi:10.1158/2767-9764.CRC-23-0331)
Supplement: Supplementary Figure 16 — Control, GAN siRNA and GAN overexpression effect on transcription factors in ME180 and HeLa cell lines [file crc-23-0331-s26.pptx]

## Slide 1
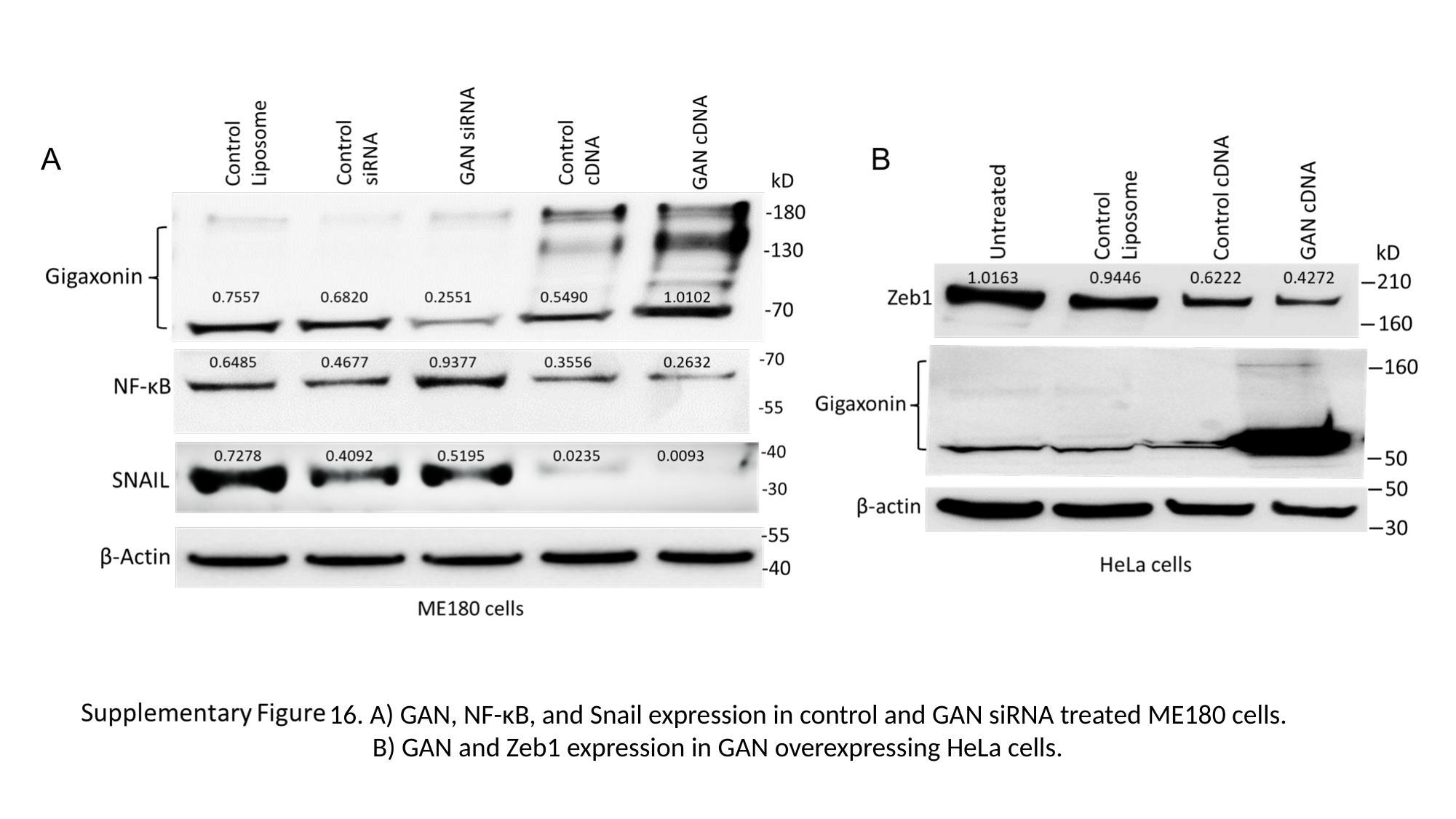

16. A) GAN, NF-κB, and Snail expression in control and GAN siRNA treated ME180 cells.
 B) GAN and Zeb1 expression in GAN overexpressing HeLa cells.
