# Supplementary material for: Gigaxonin Suppresses Epithelial-to-Mesenchymal Transition of Human Cancer Through Downregulation of Snail
Source: Cancer Res Commun. 2024 Mar 8;4(3):706–22. doi: 10.1158/2767-9764.CRC-23-0331 (PMC10921914; doi:10.1158/2767-9764.CRC-23-0331)
Supplement: Supplementary Figure 18 — E-cadherin and Snail expression in breast and prostate cancer samples [file crc-23-0331-s28.pptx]

## Slide 1
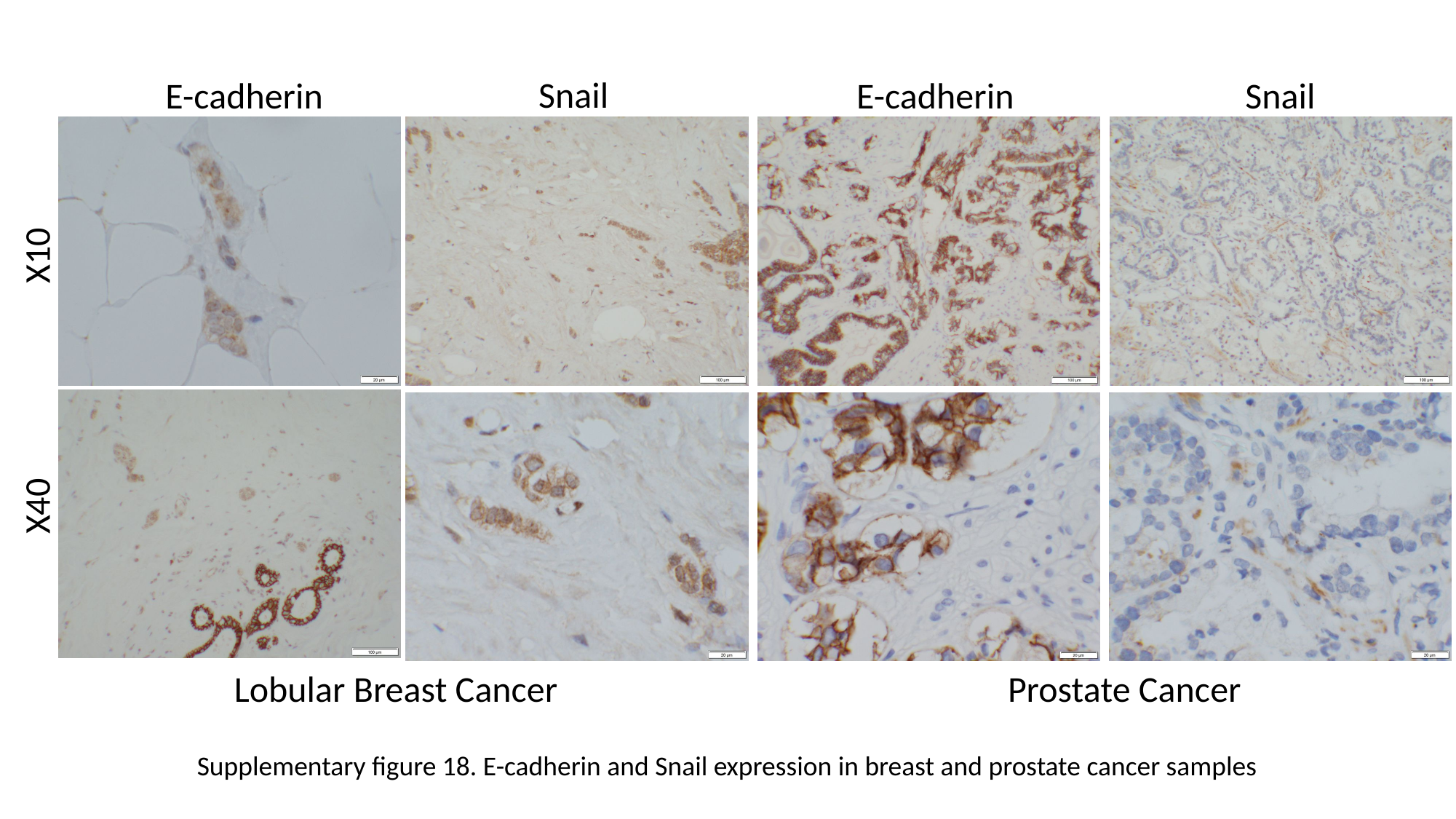

Snail
E-cadherin
E-cadherin
Snail
X10
X40
Prostate Cancer
Lobular Breast Cancer
Supplementary figure 18. E-cadherin and Snail expression in breast and prostate cancer samples
