# Supplementary material for: Gigaxonin Suppresses Epithelial-to-Mesenchymal Transition of Human Cancer Through Downregulation of Snail
Source: Cancer Res Commun. 2024 Mar 8;4(3):706–22. doi: 10.1158/2767-9764.CRC-23-0331 (PMC10921914; doi:10.1158/2767-9764.CRC-23-0331)
Supplement: Supplementary Figure 19 — Ubiquitination assay for GAN binding to Snail and NF-kB [file crc-23-0331-s29.pptx]

## Slide 1
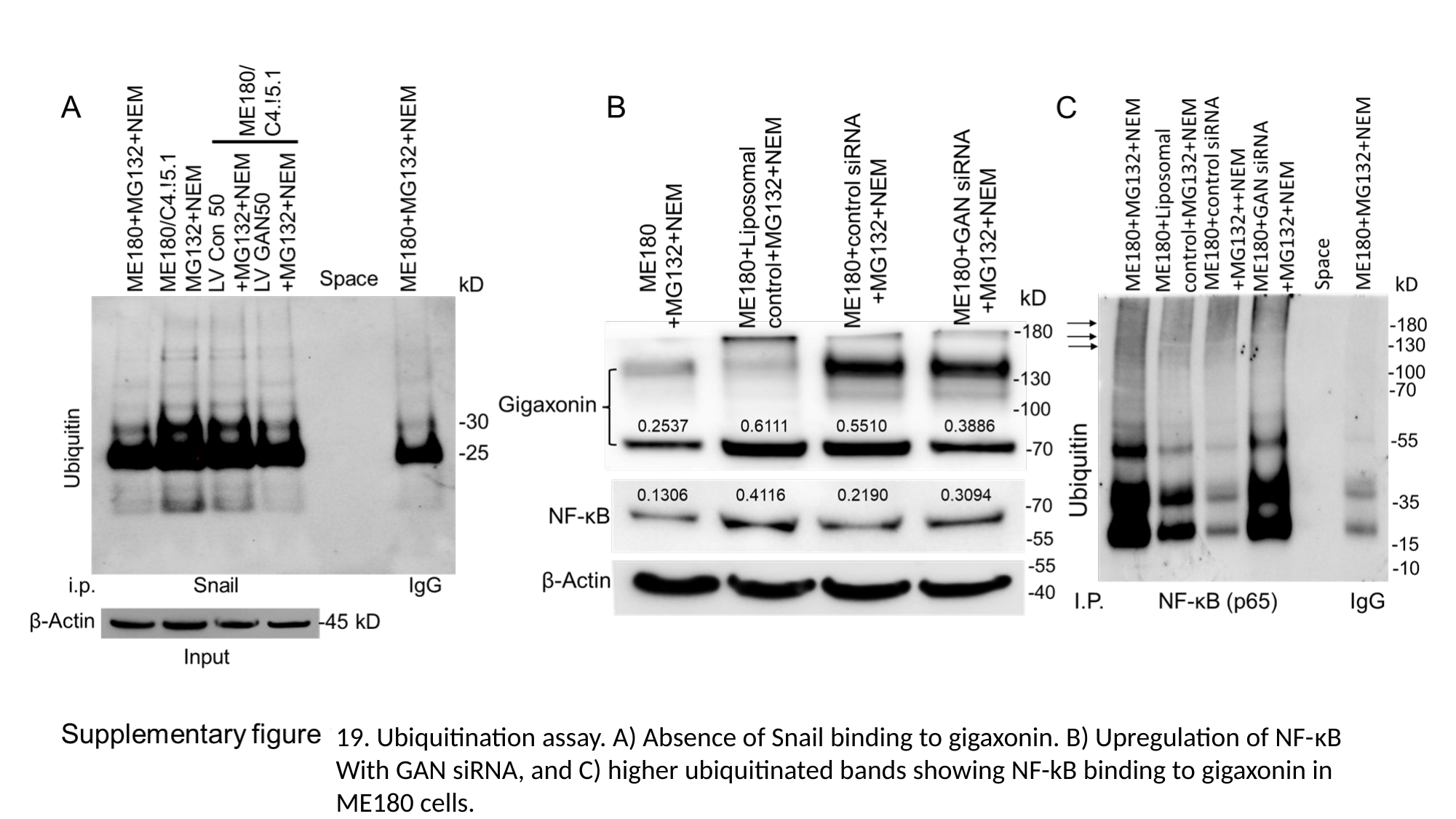

19. Ubiquitination assay. A) Absence of Snail binding to gigaxonin. B) Upregulation of NF-κB
With GAN siRNA, and C) higher ubiquitinated bands showing NF-kB binding to gigaxonin in
ME180 cells.
