# Supplementary material for: Gigaxonin Suppresses Epithelial-to-Mesenchymal Transition of Human Cancer Through Downregulation of Snail
Source: Cancer Res Commun. 2024 Mar 8;4(3):706–22. doi: 10.1158/2767-9764.CRC-23-0331 (PMC10921914; doi:10.1158/2767-9764.CRC-23-0331)
Supplement: Supplementary Figure 20 — expression of GAN, gamma H2AX, and p16 post Act D treatment of ME180 cells [file crc-23-0331-s30.pptx]

## Slide 1
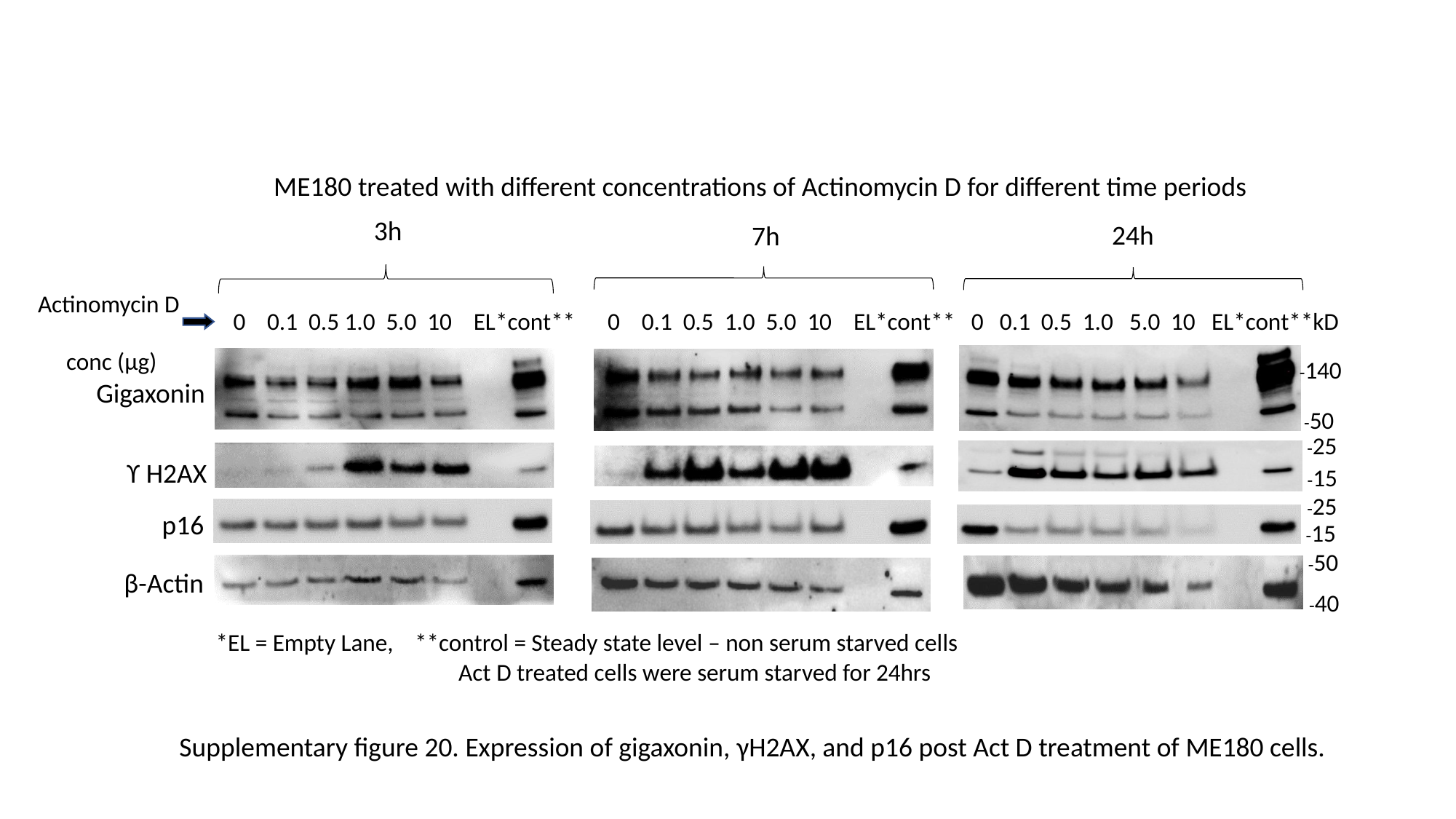

ME180 treated with different concentrations of Actinomycin D for different time periods
3h
24h
7h
Actinomycin D conc (μg)
 0 0.1 0.5 1.0 5.0 10 EL*cont** 0 0.1 0.5 1.0 5.0 10 EL*cont** 0 0.1 0.5 1.0 5.0 10 EL*cont**kD
-140
Gigaxonin
-50
-25
ϒ H2AX
-15
-25
p16
-15
-50
β-Actin
-40
*EL = Empty Lane,
**control = Steady state level – non serum starved cells
 Act D treated cells were serum starved for 24hrs
Supplementary figure 20. Expression of gigaxonin, γH2AX, and p16 post Act D treatment of ME180 cells.
