# Supplementary material for: Gigaxonin Suppresses Epithelial-to-Mesenchymal Transition of Human Cancer Through Downregulation of Snail
Source: Cancer Res Commun. 2024 Mar 8;4(3):706–22. doi: 10.1158/2767-9764.CRC-23-0331 (PMC10921914; doi:10.1158/2767-9764.CRC-23-0331)
Supplement: Supplementary Figure 21 — Survival probability of head and neck cancer [file crc-23-0331-s31.pptx]

## Slide 1
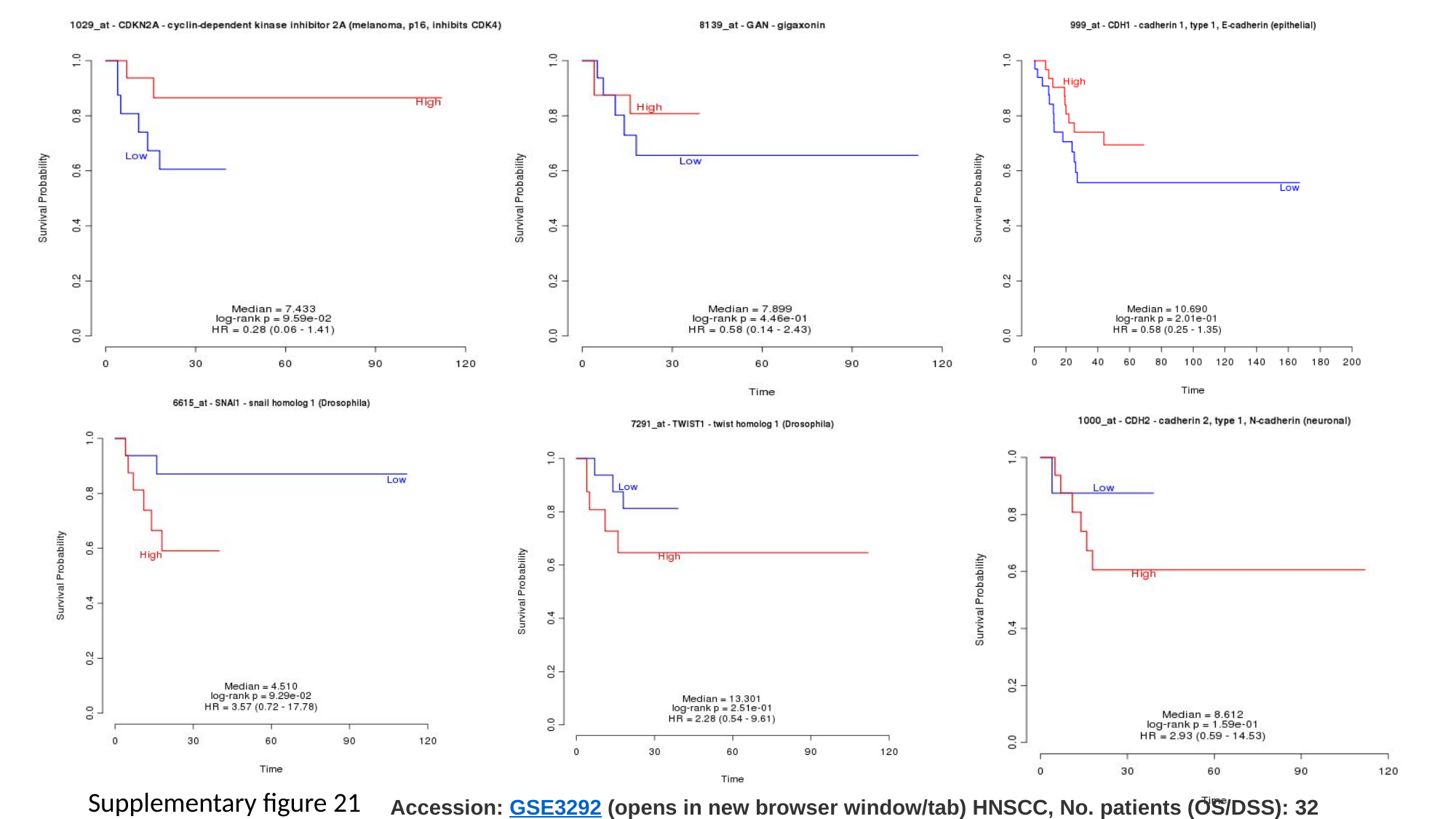

Supplementary figure 21
Accession: GSE3292 (opens in new browser window/tab) HNSCC, No. patients (OS/DSS): 32
